# Ternary Neurexin-T178-PTPR complexes represent a pre-synaptic core-module of neuronal synapse organization

**DOI:** 10.1038/s41467-026-77377-4

**Published:** 2026-09-05

**Authors:** Spyros Thivaios, Jochen Schwenk, Aline Brechet, Sami Boudkkazi, Nithya Sethumadhavan, Phil Henneken, Eriko Miura, Ayumi Hayashi, Maciej K. Kocylowski, Alexander Haupt, Debora Kaminski, Dietmar Schreiner, Agata Nowacka, Jean-Baptiste van den Broucke, Akos Kulik, Uwe Schulte, Fredrik H. Sterky, Michisuke Yuzaki, Peter Scheiffele, Bernd Fakler

**Affiliations:** 1 Institute of Physiology, Faculty of Medicine, University of Freiburg, Hermann-Herder-Str. 7, Freiburg, Germany; 2 Department of Neurophysiology, Keio University School of Medicine, Tokyo, Japan; 3 Department of Laboratory Medicine, Institute for Biomedicine, Sahlgrenska Academy, University of Gothenburg, Gothenburg, Sweden; 4 Department of Clinical Chemistry, Sahlgrenska University Hospital, Gothenburg, Sweden; 5 Biozentrum of the University of Basel, Basel, Switzerland; 6 Logopharm GmbH, Schlossstr. 14, March-Buchheim, Germany; 7 Wallenberg Centre for Molecular and Translational Medicine, University of Gothenburg, Gothenburg, Sweden; 8 Signalling Research Centres BIOSS and CIBSS, Schänzlestr. 18, Freiburg, Germany; 9 Center for Basics in NeuroModulation, Breisacherstr. 4, Freiburg, Germany

**Keywords:** Molecular neuroscience, Protein-protein interaction networks

## Abstract

Synapses, prototypic sites for neuronal communication, are key to brain function. Their organization and properties are instructed by synaptic cell adhesion molecules (sCAMs) that may operate independently or in coordination through yet unknown linker proteins. Here, we used multi-epitope affinity-purifications combined with quantitative mass spectrometry and immuno-EM to comprehensively map synaptic protein networks in the mouse brain. We identify a pre-synaptic core-module assembled from the major sCAMs, Neurexins1-3 and LAR-type receptor protein-tyrosine-phosphatases (PTPRs), and the previously uncharacterized tetraspanins T178A/B. These ternary Neurexin-T178-PTPR complexes form through their trans-membrane domains and assemble during biogenesis in the ER. Loss of T178B leads to module destabilization, accompanied by strong reduction of LAR-PTPRs and re-distribution of synaptic Neurexins. At synapses, the Neurexin-T178-PTPR module recruits stable trans-synaptic protein networks thereby interlinking machineries of the pre-synaptic active zone and establishing stable associations with post-synaptic neurotransmitter receptors. This work uncovers a widely distributed core-module for synaptic adhesion and trans-synaptic signaling in the mammalian brain.

## Abstract (web summary)

Organization and function of CNS synapses are instructed by synaptic cell adhesion molecules (sCAM). Here, authors show that the main sCAMs NRX and PTPRs are interlinked by the tetraspanins T178A,B, thus forming ternary complexes that serve as core-modules of transsynaptic networks.

## Introduction

Neuronal synapses are highly specialized units for cell-cell communication that underlie the transmission and storage of information fundamental for proper operation of the brain and its circuits. The functional properties of synapses display profound variability across the CNS and are to a significant extent instructed by synaptic cell adhesion molecules (sCAM). These sCAMs, mostly single pass (or type I) transmembrane proteins, drive formation and structural organization of neuronal synapses and regulate their stability and context-dependent dynamics^1–5^.

While individual sCAMs were found sufficient to induce neuronal synapse formation in culture systems^6–8^, their deletion by targeted gene-knockout only moderately reduced synaptogenesis in the mammalian brain^9–14^. This resilience to genetic perturbation appeared to argue for a high degree of redundancy, and raises the question of how multiple adhesion systems are interlinked by yet unknown adapters.

**Fig. 1:**
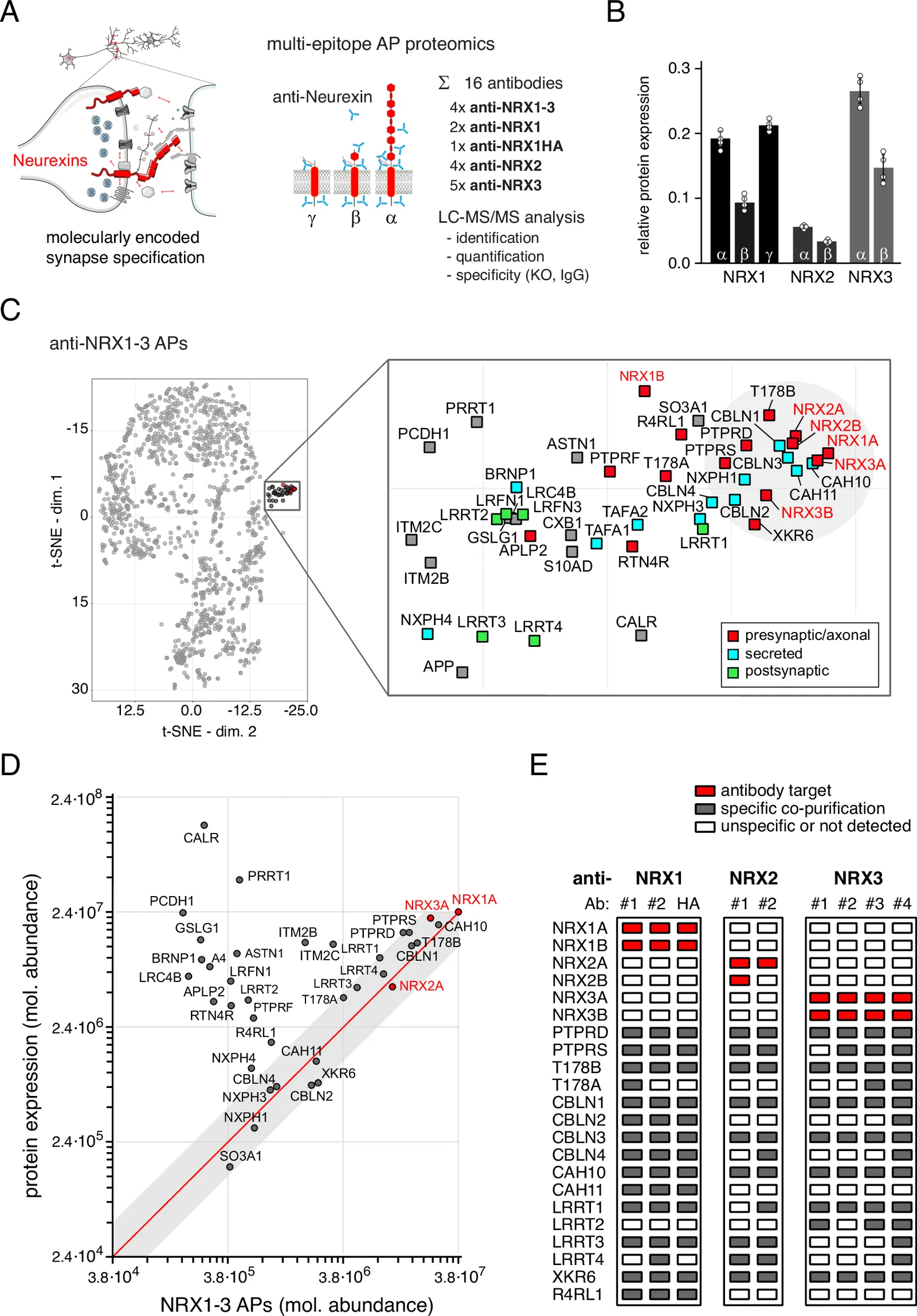
meAP-MS analysis of neurexin-associated protein assemblies. **A** Proteomic meAP-MS approach for quantitative analysis of NRX-associated trans-synaptic protein complexes. Illustration of the synapse used Servier Medical Art (https://smart.servier.com, licensed under CC BY 4.0 (https://creativecommons.org/licenses/by/4.0/)). **B** MS-derived protein abundance of all isoforms of the NRX paralogues in plasma membrane-enriched fractions from adult mouse brain (technical details in Supplementary Fig. 1). Data are mean ± SD of 4 brain samples. **C** t-SNE plot based on target normalized ratio (tnR)-values of all proteins (gray dots; Methods) identified in eight APs with the four distinct anti-NRX1-3 ABs with preimmunization IgGs and antiserum as negative controls and NRX1A as a reference for normalization. Right panel, extension of the framed area around the target proteins (NRX1-3) highlighting a cluster of consistently co-purified proteins of mainly pre-, post- and trans-synaptic origin as indicated by the color-code. **D** MS-derived molecular abundance values determined for the constituents of the NRX-cluster in (**C**) in (untreated) mouse brain membrane fractions plotted against the molecular abundance values determined in anti-NRX1-3 AP eluates. Proteins with complete binding to the NRX-target(s) are located on the diagonal in red. Area in gray defines the confidence-interval of MS-quantification. Data are mean values from four mouse brains and eluates of four independent APs with two different ABs. **E** Table summarizing a selected set of proteins specifically co-purified in APs with the indicated isoform-specific anti-NRX antibodies. Specificity was determined with target-KO (NRX1 for HA-tagged NRX1, NRX3) and IgG controls. Note that PTPRD,S and T178B are interactors of all NRX isoforms.

Among the currently known sCAMs, two families of proteins stand out based on their ubiquitous and highly abundant expression at central synapses: Neurexins (NRX) and the leukocyte common antigen-related receptor protein tyrosine phosphatases (LAR-type PTPRs). Both of these sCAM families of pre-synaptic type I transmembrane proteins comprise three distinct members, NRX1-3 and PTPRD, S and F, that display complex molecular appearances as a result of (marked) genetic variations/alternative splicing and interaction(s) with varieties of partner proteins. Thus, NRX1-3 use alternative promoters to generate long (α-neurexin) and short (β-neurexin) isoforms^15–18^; in addition, for *Nrxn1*, a very short γ-neurexin isoform is expressed from a third alternative promoter^19,20^ (Fig. 1A). NRXs contribute to pre-synaptic active zone assembly, impact Ca^2+^-dependent neurotransmitter release and instruct trans-synaptic nano-alignments in mice, flies and worms via interaction with multiple partners, including neuroligins (NLGNs), leucine-rich repeat transmembrane neuronal proteins (LRRTMs) and cerebellins (CBLNs). LAR-PTPRs exhibit evolutionarily conserved cytoplasmic interactions with key regulators of active zone assembly, most prominently complexes of SYD-1 and SYD-2/Liprin-α (LIPA) proteins^21–26^, thus tethering transmitter vesicles to the active zone (termed priming complexes^27^). Notably, recent work reported severely impaired synapse development in cerebellar circuits upon simultaneous deletion of three NRX and three LAR-PTPR proteins and led to the suggestion that their ‘combinatorial’ expression may ensure common, though mutually independent action(s)^28^. However, understanding the organizing principles and relations of the multiple sCAMs expressed at individual synapses remains a major unresolved question.

Here, we used an unbiased quantitative analysis of native protein assemblies in synaptic membranes by multi-epitope affinity-purifications (meAPs) combined with high-resolution mass spectrometry (nano-LC MS/MS) to uncover ternary complexes of NRXs, LAR-PTPRs and the tetraspanins T178A, B as a broadly expressed core-module for organization of signaling entities in the pre-synapse and for establishment of high-affinity associations with post-synaptic receptor and adhesion systems.

## Results

### Synaptic adhesion molecules are embedded into extended protein networks

Previous studies probed interactions and functions of individual sCAMs or sCAM families. To obtain a detailed map of sCAM interactomes in native tissue, we developed a comprehensive high-resolution proteomics approach combining multi-epitope affinity-purifications with quantitative mass spectrometry (meAP-MS; Methods) for an array of synaptic proteins (proteins will be referred to by the acronyms used in the UniProt/SwissProt database throughout, descriptive names or gene names may be taken from Table 1 or text). Initially, we leveraged a set of 16 AP- validated antibodies (ABs, Supplementary Tables 1, 5) targeting the primary NRXs via epitopes that are either isoform-specific (anti-NRX1/anti-NRX1HA, anti-NRX2, anti-NRX3) or present in all NRXs (anti-NRX1-3 or panNRX) and that are located at various positions along the primary sequences (Fig. 1A, left, Supplementary Table 1, Methods). The input for the meAPs were membrane-fractions from adult mouse brains (see Methods) which were treated with the detergent CL-91 established in previous work for effective solubilization of protein assemblies in synapses^29–32^. In fact, quantitative MS detected NRX1-3 in these plasma membrane-enriched fractions at distinct amounts, with NRX1 and NRX3 being the most abundant (Fig. 1B). Surprisingly, we found similar amounts for both the canonical long NRX1α and the only recently recognized short γ-isoform of NRX1, highlighting NRX1γ as a major NRX1 isoform in the mouse brain (Fig. 1B, Supplementary Fig. 1).

**Table 1:** Proteins specifically co-purified with anti-Nrx1-3 antibodies

Protein ID	Gene name	Acc.No.	Description	Mass (kDa)	Function	Refs.
NRX1A	*Nrxn1*	Q9CS84	Neurexin-1-alpha	166	synaptic organizer	^17^
NRX1B	*Nrxn1*	P0DI97	Neurexin-1-beta	50	synaptic organizer	^17^
NRX1G	*Nrxn1*	Q9CS84-6	Neurexin-1-gamma	15	synaptic organizer	^19^
NRX2A	*Nrxn2*	E9Q7X7	Neurexin-2-alpha	185	synaptic organizer	^17^
NRX2B	*Nrxn2*	E9PUN2	Neurexin-2-beta	70	synaptic organizer	^17^
NRX3A	*Nrxn3*	Q6P9K9	Neurexin-3-alpha	173	synaptic organizer	^17^
NRX3B	*Nrxn3*	Q8C985	Neurexin-3-beta	62	synaptic organizer	^17^
A4	*App*	P12023	Amyloid-beta precursor protein, A4 protein	87	axon growth	^82^
*AGRB1	*Adgrb1*	Q3UHD1	Adhesion GPCR B1	173	adhesion GPCR	^83^
*APLP2	*Aplp2*	Q06335	Amyloid beta precursor like protein 2	80	axon growth	^82^
*ASTN1	*Astn1*	Q61137	Astrotactin-1	145	surface receptor	^84^
*ASTRB	*Gramd1b*	Q80TI0	Protein Aster-B	85	transporter	^85^
*BRNP1	*Brinp1*	Q920P3	BMP/retinoic acid-inducible neural-specific protein 1	89	neuronal development	^86^
*BRNP2	*Brinp2*	Q6DFY8	BMP/retinoic acid-inducible neural-specific protein 2	89	neuronal development	^86^
C1QL3	*C1ql3*	Q9ESN4	Complement C1q-like protein 3	27	synaptic organizer	^87^
*CADM3	*Cadm3*	Q99N28	Cell adhesion molecule 3	43	cell adhesion	^88^
CAH10	*Ca10*	P61215	Carbonic anhydrase-related protein 10	38	cell adhesion	^19^
CAH11	*Ca11*	O70354	Carbonic anhydrase-related protein 11	36	cell adhesion	^19^
CALR	*Calr*	P14211	Calreticulin	48	protein folding	^89^
CBLN1	*Cbln1*	Q9R171	Cerebellin-1	21	synaptic organizer	^5^
CBLN2	*Cbln2*	Q8BGU2	Cerebellin-2	24	synaptic organizer	^90^
CBLN3	*Cbln3*	Q9JHG0	Cerebellin-3	21	synaptic organizer	^90^
CBLN4	*Cbln4*	Q8BME9	Cerebellin-4	22	synaptic organizer	^90^
*CLUS	*Clu*	Q06890	Clusterin	52	cell adhesion	^91^
*CNTN1	*Cntn1*	P12960	Contactin-1	113	cell adhesion	^92^
*GRIA1	*Gria1*	P23818	Glutamate (AMPA-type) receptor 1, GluA1	102	ion channel	^93^
*GRIA2	*Gria2*	P23819	Glutamate (AMPA-type) receptor 2, GluA2	99	ion channel	^93^
*GRIA3	*Gria3*	Q9Z2W9	Glutamate (AMPA-type) receptor 3, GluA3	101	ion channel	^93^
*GRM4	*Grm4*	Q68EF4	Metabotropic glutamate receptor 4, mGluR4	101	GPCR	^94^
*GSLG1	*Glg1*	Q61543	Golgi apparatus protein 1	134	cell adhesion	^95^
*ITM2B	*Itm2b*	O89051	Integral membrane protein 2B	30	unknown (modulator of APP processing)	^96^
*ITM2C	*Itm2c*	Q91VK4	Integral membrane protein 2C	30	unknown (modulator of APP processing)	^97^
*KCJ10	*Kcnj10*	Q9JM63	Inward rectifier K^+^ channel 10, Kir4.1	43	ion channel	^98^
*KCMA1	*Kcnma1*	Q08460	Ca^2+^-activated K^+^ channel; BK_Ca_	134	ion channel	^99^
*LRC4B	*Lrrc4b*	P0C192	Leucine-rich repeat-containing protein 4B	76	cell adhesion	^67^
*LRFN1	*Lrfn1*	Q2WF71	Leucine-rich repeat and fibronectin type III domain-containing protein 1	82	cell adhesion	^64^
*LRFN3	*Lrfn3*	Q8BLY3	Leucine-rich repeat and fibronectin type-III domain-containing protein 3	66	cell adhesion	^64^
LRRT1	*Lrrtm1*	Q8K377	Leucine-rich repeat transmembrane neuronal protein 1	59	cell adhesion	^65^
LRRT2	*Lrrtm2*	Q8BGA3	Leucine-rich repeat transmembrane neuronal protein 2	59	cell adhesion	^65^
LRRT3	*Lrrtm3*	Q8BZ81	Leucine-rich repeat transmembrane neuronal protein 3	66	cell adhesion	^66^
LRRT4	*Lrrtm4*	Q80XG9	Leucine-rich repeat transmembrane neuronal protein 4	67	cell adhesion	^63^
*MGLYR	*Gpr158*	Q8C419	Metabotropic glycine receptor, mGlyR, GPCR 158	134	GPCR	^55^
NLGN1	*Nlgn1*	Q99K10	Neuroligin-1	94	cell adhesion	^100^
NLGN2	*Nlgn2*	Q69ZK9	Neuroligin-2	91	cell adhesion	^100^
NLGN3	*Nlgn3*	Q8BYM5	Neuroligin-3	91	cell adhesion	^100^
*NSG2	*Nsg2*	P47759	Neuronal vesicle trafficking-associated protein 2	19	vesicle transport	^101^
NXPH1	*Nxph1*	Q61200	Neurexophilin-1	31	cell adhesion	^102^
NXPH3	*Nxph3*	Q91VX5	Neurexophilin-3	28	cell adhesion	^102^
NXPH4	*NXPH4*	O95158	Neurexophilin-4	33	cell adhesion	^102^
*PCDH1	*PCDH1*	Q08174	Protocadherin-1	115	axon growth	^103^
*PRRT1	*Prrt1*	O35449	Proline-rich transmembrane protein 1	31	synaptic plasticity	^104^
PTPRD	*Ptprd*	Q64487	Receptor-type tyrosine-protein phosphatase D	214	synaptic organizer	^105^
PTPRF	*Ptprf*	A2A8L5	Receptor-type tyrosine-protein phosphatase F	211	synaptic organizer	^105^
PTPRS	*Ptprs*	B0V2N1	Receptor-type tyrosine-protein phosphatase S	212	synaptic organizer	^105^
*R4RL1	*Rtn4rl1*	Q8K0S5	Reticulon-4 receptor-like 1	50	axon guidance	^106^
*RGS7	*Rgs7*	O54829	Regulator of G-protein signaling 7	55	GPCR	^55^
*RTN4R	*Rtn4r*	Q99PI8	Reticulon-4 receptor, NoGo-receptor	51	axon guidance	^106^
*SLIT1	*Slit1*	Q80TR4	Slit homolog 1 protein	167	axon guidance	^107^
*SLIT2	*Slit2*	Q9R1B9	Slit homolog 2 protein	169	axon guidance	^107^
*SLIT3	*Slit3*	Q9WVB4	Slit homolog 3 protein	168	axon guidance	^107^
*SO3A1	*Slco3a1*	Q8R3L5	Solute carrier organic anion transporter family member 3A1	77	transporter	^108^
SYT11	*Syt11*	Q9R0N3	Synaptotagmin-11	48	vesicle protein	^109^
*T132A	*Tmem132a*	Q922P8	Transmembrane protein 132A	110	unknown	^56^
*T178A	*Tmem178a*	Q9CZ16	Transmembrane protein 178A	33	unknown	
*T178B	*Tmem178b*	H3BS89	Transmembrane protein 178B	33	unknown	
TAFA1	*Tafa1*	Q7TPG8	Chemokine-like protein TAFA-1, FAM19A	15	cell adhesion	^38^
TAFA2	*Tafa2*	Q7TPG7	Chemokine-like protein TAFA-2, FAM19B	15	cell adhesion	^38^
*TRPV2	*Trpv2*	Q9WTR1	Transient receptor potential channel TRPV2	86	ion channel	^110^
*VWA8	*Vwa8*	Q8CC88	von Willebrand factor A domain-containing protein 8	213	unknown	
*XKR4	*Xkr4*	Q5GH67	XK-related protein 4	72	scramblase	^111^
*XKR6	*Xkr6*	E9Q6C8	XK-related protein 6	71	unknown	

* proteins not previously related to NRX.

**Interactome of native NRX1-3** (identified by meAP-MS with anti-NRX1-3 ABs)

All proteins identified by quantitative MS-analysis in the APs with the four different anti-NRX1-3 ABs were evaluated by determination of tnR-values (Methods, text) using target-unrelated ABs and pre-immunization IgGs as negative controls. All proteins with tnR-values >0.25 in APs with at least two of the four ABs were considered as specific interactors of NRXs; the entirety of these specific interactors is referred to as the NRX-interactome.

Accordingly, the NRX-interactome comprises additional constituents compared to the t-SNE cluster (Fig. 1C) of proteins consistently retrieved in APs with all four NRX1-3 ABs.

To uncover native NRX-associated protein complexes, the four independent anti-NRX1-3 ABs were used in a first round of APs as target-specific ABs, together with pools of pre-immunization IgGs and pre-immunization sera as negative controls. All resulting AP-elutions were analyzed by high-resolution nano-LC MS/MS and evaluated by label-free quantification based on peak-volumes (PVs) and offering a linear dynamic range of about four orders of magnitude^29,33,34^. The respective results showed effective purification of the NRX paralogues as indicated by large PVs and broad coverage of the primary sequences by MS/MS-identified peptides (Supplementary Fig. 2). In addition to the NRX targets, the APs retrieved a number of additional proteins that were evaluated for their target-specific co-purification, as well as for consistency and direct comparability across the individual APs. The latter was done by determining for any protein target-normalized ratios (tnRs^35^, Supplementary Fig. 3A) which is the relative abundance of a protein related to both, AP-background (minimal value derived from negative controls) and AP-target (maximal value; Methods, Supplementary Fig. 3A). When analyzed by t-SNE (t-distributed stochastic neighbor embedding), the resulting plot of the tnR-values obtained in APs with the four anti-NRX1-3 ABs (Supplementary Fig. 3B and Table 1) immediately identified a well-separated cluster of 45 proteins that tightly co-localized around the targets, thus indicating robust and consistent co-purification with the anti-NRX1-3 ABs in all APs (Fig. 1C and Supplementary Table 2). Closer inspection uncovered notable features of these target-clustered proteins (Fig. 1C, inset, Table 1): (1) They are components of the pre- and post-synaptic compartments, and the synaptic cleft, (2) most identified proteins are either single-pass trans-membrane or soluble/secreted proteins, and (3) several of them were established interaction partners of the NRX proteins, such as neurexophilins (NXPH1-4^36^), cerebellins (CBLN1-3^37^), carbonic anhydrase-related (CAH) proteins 10, 11^19^, chemokine-like proteins TAFA1,2^38^, amyloid-β precursor protein (A4 or APP^39^), receptor protein tyrosine phosphatase S (PTPRS^40^,) or leucine-rich repeat transmembrane neuronal (LRRT) proteins 1-4^40–42^. In addition, the cluster contained a substantial number of proteins that were previously unknown to be associated with NRXs. These proteins include the tetraspanins T178A and T178B, the putative lipid scramblase XK-related protein 6 (XKR6), reticulon-4 receptor (RTN4R, also termed Nogo receptor) and reticulon-4 receptor like 1 (R4RL1), as well as amyloid-β precursor-like protein (APLP2), ITM2B, C^32^, and the LAR-type receptor protein tyrosine phosphatases PTPRD and F. Interestingly, quantitative analysis of protein abundances in both source material (protein expression) and elutions of NRX1-3 APs showed that PTPRs D, S and the tetraspanin T178B are for most parts associated with the NRXs and that these co-assemblies contain the vast majority of expressed NRX proteins in the brain (Fig. 1D, diagonal in red). Thus, NRXs, LAR-PTPRs and T178B are not only co-expressed across the entire brain (Supplementary Fig. 4) but apparently occur in stable stoichiometric complexes, rather than as independent adhesive units. In addition, these analyses showed that more than half of the NRXs are covalently linked to CAH10 ^19^, and interact with the secreted CBLN proteins (Fig. 1D).

In a second round of APs, we next used the isoform-specific anti-NRX ABs and target-knockouts (Supplementary Fig. 5) as stringent negative controls to uncover whether the aforementioned novel interactors associate selectively with any of the NRX paralogues or whether they co-assemble with all primary NRX proteins. Importantly, we discovered that NRX1, 2, and 3 do not mutually co-purify (Fig. 1E) indicating that they form separate molecular entities. While some of the aforementioned interactors, like CAH11, R4RL1 and LRRTM2, exhibited preferences for individual NRXs, others co-assembled with all three NRX paralogues (Fig. 1E and Supplementary Table 2). Most prominently, PTPRs and T178B were effectively retrieved in all NRX APs (Supplementary Fig. 3B).

Together, these meAP-MS data demonstrated that in the adult brain the three NRX paralogues form separate trans-synaptic modules with numerous interactors. However, all three NRXs co-assemble with T178B and LAR-PTPRs into previously unrecognized stoichiometric complexes.

### Neurexins form ternary modules with T178 and LAR-type PTPRs

**Fig. 2:**
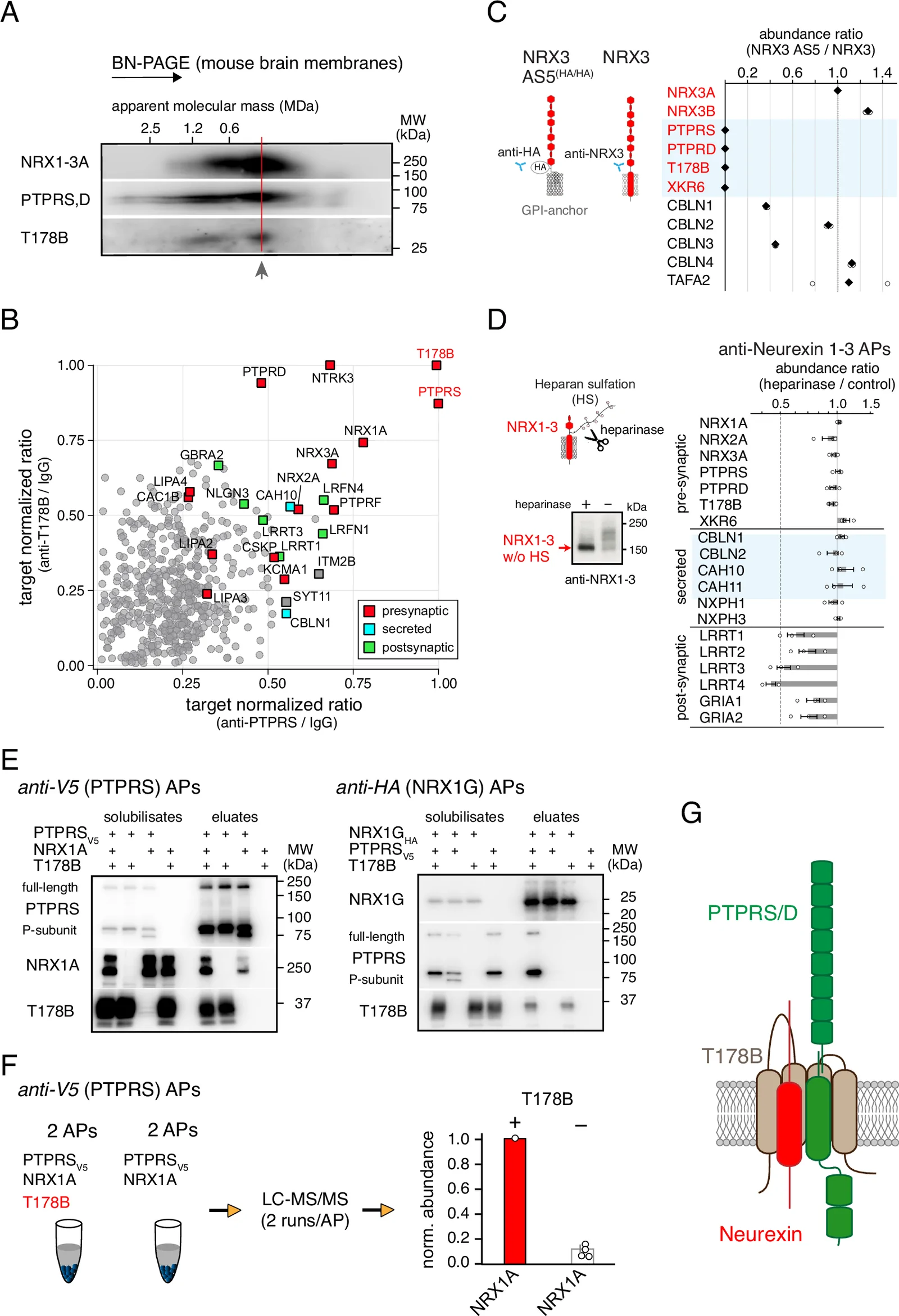
Ternary complex formation of Neurexins with LAR-type PTPRs and the tetraspanin T178B. **A** Native gel separation (BN-PAGE) of protein complexes in CL47-solubilized membrane fractions from adult mouse brain Western-probed for the indicated proteins (repeated four times). Note close co-migration of NRX1-3A, PTPRD/S and T178B as tripartite complex with an apparent molecular mass of ∼400 kDa (arrowhead). Fainter signals extending to an apparent mass of 1.2 MDa reflect their integration into larger assemblies. **B** tnR-plot comparing APs with ABs targeting T178B and PTPRS indicates robust co-purification of NRX1-3 and a variety of pre- and post-synaptic interactors. tnR-values are related to IgGs as a reference. Color-code of subcellular localization as indicated. **C** Abundance-ratios determined for proteins affinity-purified in anti-HA APs from HA-Neurexin3 AS5 (GPI-anchored variant of NRX3, inset) mice and in anti-NRX3 APs from wild-type mice. Note that GPI-anchored NRX3 complexes lack association with pre-synaptic PTPRD,S, T178B and XKR6, whereas binding of extracellular ligands was maintained. Experiments used two distinct HA and two NRX3 ABs, values are mean of two independent experiments. **D** Abundance-ratio for a subset of proteins affinity-purified with anti-NRX1-3 from CL91-solubilized brain membrane fractions with and without heparinase treatment; inset: Western-probed gel-separation indicating heparinase-mediated reduction in size of NRX3. Enzymatic activity strongly reduced co-purification of post-synaptic proteins (LRRTs, AMPAR subunits GRIA1, 2), while binding to pre-synaptic and extracellular/secreted interactors remained unchanged. Data are mean ± SEM of 3 experiments. **E** Reconstitution of ternary complexes assembled from PTPRS, NRX1A/1 G and T178B in cultured tsA cells. Specific co-purification of T178B and NRX1A with V5-tagged PTPRS in anti-V5 APs, and of T178B and PTPRS_V5_ with HA-tagged NRX1G in anti-HA APs. Presence of T178B is critical for stable complex formation with NRX1A/1G and impacts maturation of the P-subunit of PTPRS. **F** Quantification of T178B-mediated increase in interaction of NRX1A and PTPRS_V5_ in two independent anti-V5 APs. Data are normalized abundances of NRX1A determined in two anti-V5 APs with two controls (bars are mean ± SD). **G** Scheme of the ternary complex derived from the pairwise interaction assays (**C**, **E**, also Supplementary Fig. 6A) and the AP results with NRX3-GPI.

The co-assembly of NRXs with T178 and LAR PTPRs was further investigated by native gel electrophoresis (BN-PAGE) and reverse APs. BN-PAGE analysis of brain membrane fractions indicated co-migration of NRX1-3A, LAR-PTPRs and T178B at an apparent molecular mass of roughly 400 kDa (Fig. 2A, arrowhead) consistent with complex formation at an equimolar (or 1:1:1) stoichiometry based on predicted protein mass (Table 1). In addition, the BN-PAGE demonstrated assembly of the ternary complex with multiple additional partners/interactors as reflected by the extension of all three Western blot signals into the higher apparent mass range (Fig. 2A). Orthogonal (reverse) APs with ABs against both, PTPRS and T178B, robustly retrieved the NRX proteins together with multiple of their interaction partners as shown by the respective tnR-values (Fig. 2B). Accordingly, the ternary NRX-T178-LAR-PTPRs complex may be considered a ‘core-module’ of larger assemblies whose constituents were consistently and robustly co-purified in the meAP experiments (Figs. 1C, E, 2B, and Table 1).

Next, we investigated the structural determinant(s) for the assembly of the ternary complex in native tissue by comparative APs (Fig. 2C, D, insets) dissecting the role of the extracellular domain versus the transmembrane and intracellular stretches, and the significance of the heparan sulfate moieties^40,43,44^. We targeted the endogenous GPI-anchored NRX3 AS5 splice variant tagged through the knock-in of an HA-epitope (NRX3 AS5^(HA/HA)^^15^,) and compared the recovery of the complex constituents to APs of total NRX3 (Fig. 2C). MS-based quantification indicated that assembly into the ternary complex is abolished for the splice-variant lacking the transmembrane domain, while binding of the soluble interactors (CBLNs or TAFAs) to the extracellular domains of NRX3 was largely preserved in the NRX3 AS5 proteoform (Fig. 2C). In contrast, enzymatic removal of heparan sulfation prior to APs (^43^, Fig. 2C, inset) markedly decreased co-purification of post-synaptic interactors (including LRRTs and AMPA-type glutamate receptors (AMPARs)), but neither affected assembly of NRXs with T178B and PTPRs, nor interaction with the extracellular NRX ligands CBLNs or CAH10 (Fig. 2D). Moreover, assembly of the core-module did not require any additional components as indicated by the robust complex formation observed upon heterologous expression of all three constituents (Fig. 2E and Supplementary Fig. 6). Notably, T178B effectively binds to both PTPRS and NRX1 and 3, while efficient co-assembly between PTPRS and NRX1A/G or NRX3A was only observed in the presence of T178. In fact, quantitative analysis by MS indicated that interaction between NRX and PTPR observed upon their pairwise expression (Fig. 2E and Supplementary Fig. 6) was increased 10-fold by T178B (Fig. 2F). Likewise, binding of GPI-anchored NRX3A to PTPRS was weak and independent of T178B (Supplementary Fig. 6A). Thus, the transmembrane domain of NRX and the short cytoplasmic domain are obligatory for formation of the ternary core-module, while the extended extracellular portion of the NRX protein is not able to promote stable core-module assembly.

These findings led us to a working model of the ternary core-module where the transmembrane domains of NRX and PTPR are juxtaposed and surrounded by the transmembrane segments of T178B (Fig. 2G). A similar arrangement was predicted by the AlphaFold3 algorithm when fed with the identified interaction domains (Supplementary Fig. 6B). In this framework, T178B is expected to closely link the two major classes of sCAMs, and to generate a macro-molecular complex with an extended surface for protein-protein interactions both in the membrane plane, as well in the aqueous phases on either side of the membrane.

### Assembly in the ER and preferred localization of the core-module(s) to the plasma membrane

The structural arrangement of the ternary core-module and the association of its constituents with multiple partner proteins prompted the questions on where within the cellular membrane frameworks co-assembly of the core-module(s) and their partners occurs and what may happen upon targeted removal of the interlinking T178B protein.

**Fig. 3:**
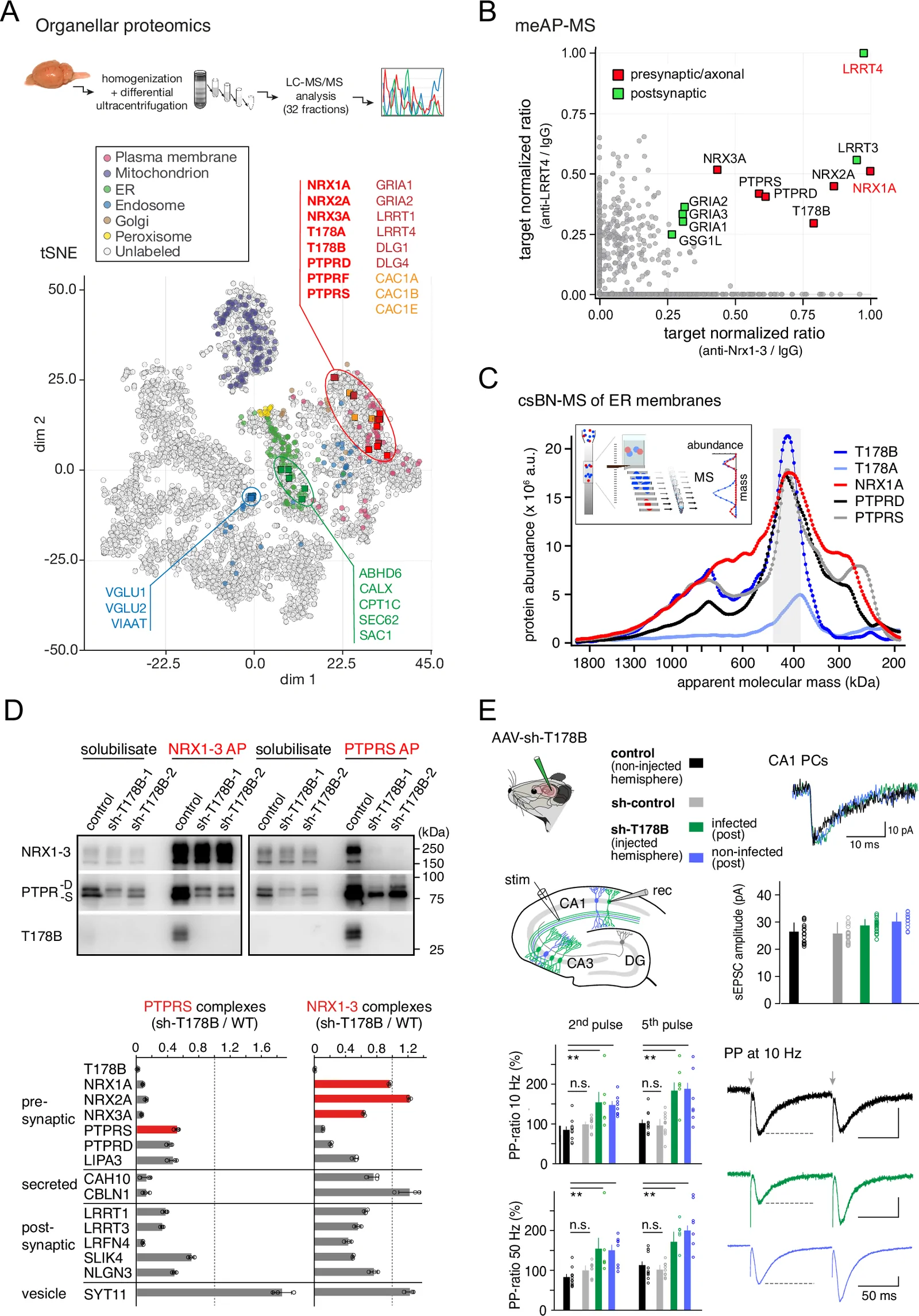
Distribution and biogenesis of core-module complexes and alteration of assembly and pre-synaptic functions upon knock-down of T178B. **A** Organellar proteomics of brain homogenates. Upper panel, key steps of the approach (see Methods, Supplementary Fig. 7). Lower panel, t-SNE plot of all individual MS-identified and quantified proteins (circles); markers for distinct organellar compartments^46^ are color-coded as indicated. Circled areas indicate close co-localization of selected proteins in the plasma membrane (red), synaptic vesicles (blue) and endoplasmic reticulum (green). Note that all subunits of the ternary core-module show preferential localization at the synaptic plasma membrane. **B** tnR-plot of APs with ABs targeting post-synaptic LRRT4 and pre-synaptic NRX1-3 highlights common co-purification of PTPRs D, S and T178B and subunits of AMPA-receptors. tnR-values were determined with IgGs as a reference.** C** Abundance-mass profiles obtained for the indicated proteins by csBN-MS in CL-47 solubilized ER-enriched membrane fractions from adult mouse brain. Inset: Scheme of the csBN-MS approach used^34,49^. Note close co-migration of NRX1A, PTPRD,S and T178A, B indicating their co-assembly into complexes of variable apparent mass. Values of protein abundance indicate close to stoichiometric association (see text). **D** Alterations in PTPR and NRX complexes after knockdown of T178B. Upper panel, Input and eluates of APs with anti-PTPRS and anti-NRX1-3 APs from CL91-solubilized membrane fractions of cultured neurons transduced with two different AAV-sh-T178B viruses or control virus (WT) Western-probed with ABs specific for NRX1-3, PTPRS, D or T178B. Lower panel, Abundance-ratios determined for proteins specifically purified in three anti-PTPRS and anti-NRX1-3 APs from WT and AAV-sh-T178B-2 transduced cultures by quantitative MS. Data are mean ± SD of three experiments, values for the AP targets are marked in red. **E** Alterations in pre-synaptic function induced by AAV-sh-T178B in hippocampal neurons of adult mice four weeks after stereotactic injection (inset, upper left). Upper right panel, Bar graph summarizing amplitudes of AMPAR-mediated sEPSCs and representative EPSC traces measured in CA1 pyramidal cells (CA1 PCs) in the un-injected hemisphere (control, WT, black) and in infected (green) and non-infected (blue) PCs of the injected hemisphere (inset, lower left). Data are mean ± SEM of 17 (WT), 20 (infected) and 9 (non-infected) PCs in sh-T178B-infected hemispheres (*p* values < 0.01 (0.007 ≤ *p* ≤ 0.009) between control and sh-injected hemisphere, two-tailed Mann–Whitney *U* test) and of 27 PCs (gray) in sh-control-infected hemispheres. Lower panel, Bar graphs summarizing paired-pulse (PP) ratios determined in CA1 PCs with stimulation frequencies of 10 and 50 Hz and representative AMPAR-mediated current traces recorded at 10 Hz, color-coding of expression manipulation as in the upper panel. Data are mean ± SEM of 11 (WT), 6 (infected) and 8 (non-infected) PCs in sh-T178B-infected hemispheres (*p* values < 0.01 (0.001 ≤ *p* ≤ 0.005) between control and sh-injected hemisphere, two-tailed Mann–Whitney *U* test) and of 15 PCs in sh-control-infected hemispheres. Note the marked increase in PP-ratio of AMPAR-currents triggered by sh-T178B infected pre-synapses in both infected and non-infected CA1 PCs.

For this purpose, we performed a series of experiments starting out with organellar proteomics on 32 fractions derived from whole brain homogenates (^45^, Fig. 3A, upper panel, Supplementary Fig. 7) for probing the preferred sub-cellular localization of the individual core-module constituents. Analysis of the MS-derived molecular abundance values determined for all proteins in these fractions by t-SNE showed that all NRX paralogues and LAR-PTPRs are colocalized with the T178 proteins in a cluster delineated by established marker proteins of the plasma membrane (^45,46^, Fig. 3A). Similarly, a series of synaptic proteins such as AMPAR subunits, LRRTs, DLGs (also known as PSD proteins) or the family 2 voltage-gated calcium (Ca^2+^) channels (CAC1A (Cav2.1), CAC1B (Cav2.2), CAC1E (Cav2.3)) were found in this cluster, which itself appears clearly separated from other membrane compartments including ER, endosomes, Golgi apparatus or mitochondria (Fig. 3A). This strongly preferred sub-cellular localization to the plasma membrane was further confirmed by meAP-MS experiments targeting the abundant post-synaptic LRRT4 protein that effectively retrieved the core-module(s) together with pore and auxiliary subunits of AMPARs (^31,47^, Fig. 3B). Moreover, immunostaining of endogenous T178B detected via a knocked-in HA-epitope tag (Methods) independently confirmed localization of this tetraspanin to the synaptic compartment and co-localization with NRXs (Supplementary Fig. 8).

The observed localization of the vast majority of the core-module constituents (NRXs, LAR-PTPRs and T178) to the plasma membrane does, however not exclude their co-assembly at earlier stages of membrane protein biogenesis. We, therefore, investigated their molecular appearance in ER-enriched membrane fractions^45^ by complexome-profiling via csBN-MS, a technique that combines native gel electrophoresis with quantitative MS (Fig. 3C, inset^34,48,49^). The abundance-mass profiles obtained with this technique demonstrated effective co-assembly of NRX1A, T178B and PTPRS, D in the ER, most prominently reflected by the profile-peaks at an apparent mass of around 400 kDa (Fig. 3C, shaded), comparable to the result obtained with the plasma membrane-enriched fractions before (Fig. 2A). The abundance-peak of T178A at a slightly smaller mass (∼360 kDa) together with the broadening (‘shoulder’) of the peaks in the LAR-PTPRs and NRX1A profiles argues for preferred co-assembly of this tetraspanin with variants of the PTPRs and NRXs. In addition, the ER-profiles also showed formation of higher-mass assemblies (up to 1.2 MDa) indicating association of the core-module with further interactors. We explored the subunits of these assemblies by additional APs with anti-NRX1-3 and anti-PTPRS ABs and identified association with liprin-α2 (LIPA2), Cav2.2 (CAC1B), CAH10 and CBLN1 amongst other proteins (Supplementary Table 3). Thus, our csBN-MS data identified formation of the ternary core-module during biogenesis in the ER and suggested its delivery to the plasma membrane as a pre-formed entity together with co-assembled partners.

### Re-organization of NRXs in the pre-synapse upon knock-down of T178B

Next, we explored the consequences of targeted knockdown of the NRX-PTPR-linking T178B protein by virally-driven expression of two different shRNAs (sh-T178B-1, −2) in both neuronal cultures and hippocampal neurons in mice.

In cultured neurons, AAV-delivered sh-T178B decreased the amount of the T178B protein present in (un-solubilized) membrane fractions by more than 95% as determined by quantitative MS (Supplementary Fig. 9A). More detailed analysis showed that this decrease was target-selective leaving the amounts of T178A, as well as of the vast majority of proteins including the NRX paralogues in this fraction either unaltered or changed by ∼10% or less (Supplementary Fig. 9A, B (red lines)). In contrast, the knockdown of T178B resulted in a reduction of both PTPRD and S by ∼30-40% (Supplementary Fig. 9B), closely matching the reduction induced by targeted knock-out of both T178A,B (Supplementary Fig. 9C).

These changes in expressed protein amounts translated into profound alterations of the core-module and its interactions, assessed by quantitative meAP-MS experiments with anti-PTPRS and anti-NRX1-3 ABs (Fig. 3D). Accordingly, the loss of T178B led to disruption of the NRX-PTPR assembly, exceeding by far the reduced interaction expected from the decrease in PTPR amounts (Supplementary Fig. 9D). The remaining NRXs and PTPRs were left either as separate proteins or, as a minor portion of bi-molecular NRX-PTPR assemblies. In addition, the NRXs lost 30–60% of their post-synaptic interactors, such as LRRTs or NLGNs, as well as PTPR-linked pre-synaptic partners, such as liprins (LIPA3) or proteins LRFN (leucine-rich repeat and fibronectin type-III domain containing). Finally, PTPRS was largely deprived of its NRX-recruited interactors, including the CBLNs (Fig. 3D).

The significance of the knockdown-induced changes was further investigated in the hippocampus of adult mice where AAV-sh-T178B or AAV-sh-control was delivered into the hippocampus (CA3 region, Supplementary Fig. 10*)* of one hemisphere by stereotactic injection, while the other was left untouched for the use as wildtype control (WT, Methods). As illustrated in Fig. 3E, whole-cell recordings performed in acute slices detected AMPAR-mediated excitatory post-synaptic currents (EPSCs) in response to spontaneous action potentials in pyramidal neurons of the CA1 region (CA1 PCs) in both hemispheres with similar time course and amplitude (Fig. 3E, upper panel). In contrast, EPSCs evoked by electrical stimulation of the Schaffer collaterals (Fig. 3E, inset), the axons of CA3 PCs serving as pre-synaptic input to the CA1 PCs, uncovered marked and specific differences between the hemispheres and between sh-178B and sh-control injections. While control CA1 PCs responded with constant EPSCs in paired-pulse (PP) recordings (PP-ratio ∼100%) at stimulation frequencies of 10 and 50 Hz, PCs in the injected hemispheres displayed a marked stimulation-dependent increase in their EPSC amplitudes (PP-ratios of 150–200%; Fig. 3E, lower panel) for sh-T178B, but not for sh-control (Fig. 3E). This increase in PP-ratio was induced by the knock-down of T178B in the pre-synaptic terminals, but was independent from knock-down in the post-synaptic neuron, pointing towards altered pre-synaptic function(s) in the absence of T178B (Fig. 3E, lower panel, Supplementary Fig. 10). The impact/effect of T178B on pre-synaptic function was further corroborated in medial perforant path (MPP) synapses onto dentate gyrus granule cells (GC). Selective pre-synaptic removal of T178A, B resulted in decreased PP-ratios and higher release probability without alterations in the AMPA-/NMDA-receptor ratio (Supplementary Fig. 11).

Together, these results on selective removal/reduction of T178B indicated the significance of this protein for the formation of the ternary NRX-T178-PTPR complexes and demonstrated the importance of their structural integrity for normal synaptic transmission. Notwithstanding, they also showed that the remaining entities enabled operation of pre-synapses, albeit with characteristics that were different from normal, presumably as a consequence of altered protein arrangements/assemblies.

**Fig. 4:**
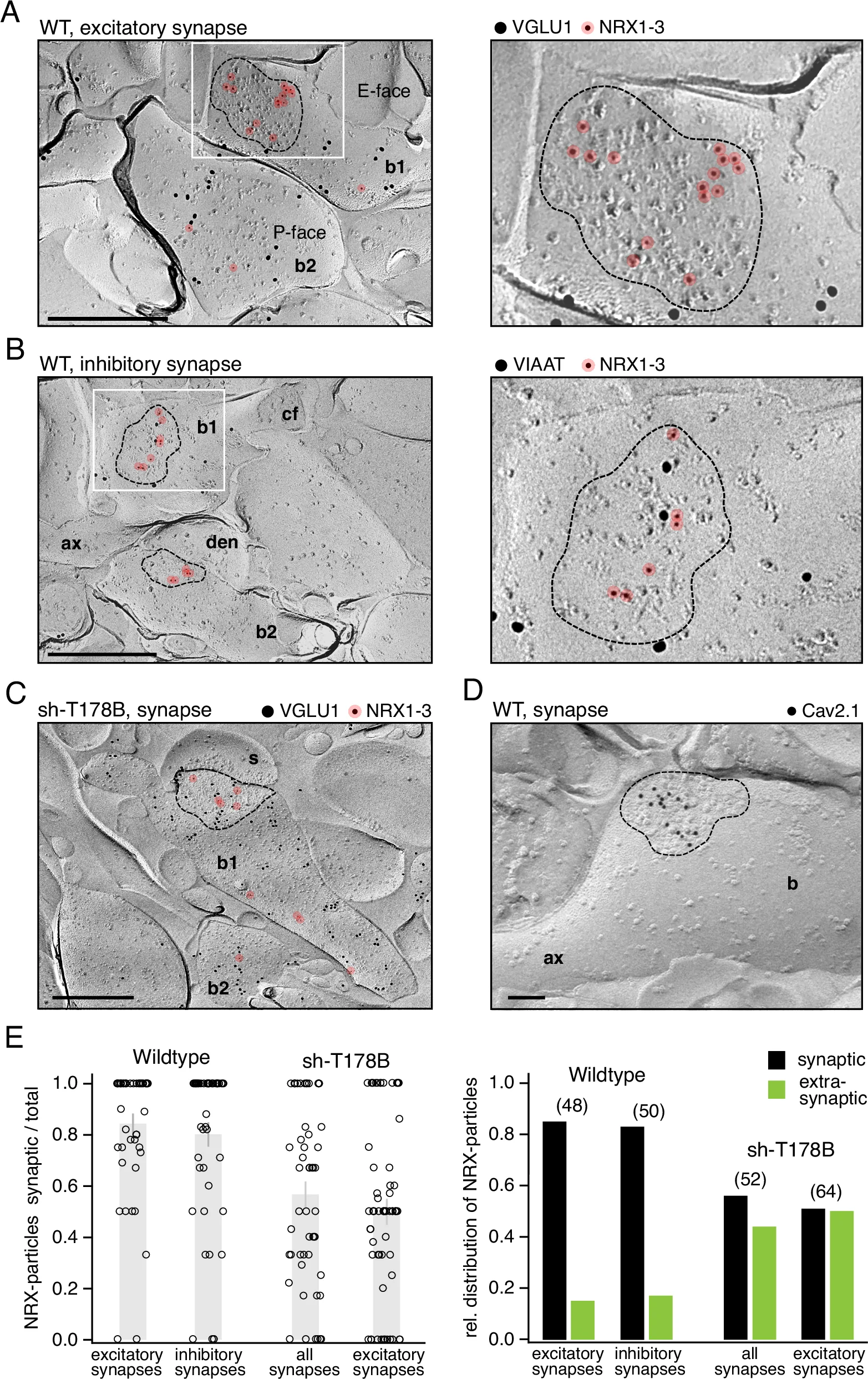
Localization of NRX1-3 to pre-synaptic active zones and re-distribution following knockdown of T178B. **A**, **B** Left panel, electron micrographs illustrating predominant localization of immuno-gold particles labeling NRX1-3 (6 nm particles, red overlay) to the active zone (delineated by broken line) and occasionally to the extra-synaptic membrane of excitatory and inhibitory synaptic boutons (b) in the hippocampal CA1 region of wildtype mice. Immuno-particles for the vesicular transporters of glutamate (VGLU1, 18 nm particles) and GABA (VIAAT, 18 nm particles) were used as markers for excitatory and inhibitory terminals, respectively. Right panel, framed area on the left at extended scale. In **B** NRX1-3 particles were additionally observed in the active zone of a VIAAT-negative putative excitatory bouton (b2). **C** Immuno-gold particles for NRX1-3 (12 nm, red overlay) localized to both the active zone (delineated by broken line, making synaptic contact with a post-synaptic spine (s)) and to extra-synaptic membrane areas of pre-synaptic boutons (b1, b2). **D** Clustered organization of Cav2.1 channels (12 nm particles) in the active zone of a pre-synaptic bouton. **E** Quantitative assessment of the distribution of NRX1-3-particles to synaptic and extra-synaptic membranes of excitatory and inhibitory terminals in hippocampal CA1 regions of WT and sh-T178B-injected mice (‘all synapses’ refers to the combined VGLU1-positive and -negative synapses). Data obtained from 48 (excitatory, WT), 50 (inhibitory, WT), 50 (all synapses, sh-T178B) and 64 (excitatory, sh-T178B) boutons are illustrated individually (left panel, empty circles, gray bars indicate mean ± SD of the individual terminals) or as pooled data obtained in all terminals (right panel). Abbreviations: ax, preterminal axon; cf, cross-fractured face of bouton; P-face, protoplasmic face; E-face, exoplasmic face. Scale bars are 500 nm.

This hypothesized re-organization of the core-module(s) upon knockdown of T178B was further pursued by analyzing the distribution of the NRXs in freeze-fracture replicas of the hippocampal CA1 region of WT and sh-T178B-injected mice by electron microscopy combined with immuno-gold-labeling with an NRX1-3 AB (Supplementary Table 5) that was carefully evaluated for target-specificity and target-efficiency by AP-MS^29,45,50,51^. Quantitative assessment of gold-particles showed that NRX1-3 exhibit a strong preference for (localization to) the active zone, in line with a previous report^52^. This preference was observed for both excitatory and inhibitory synapses identified by parallel labeling for the vesicular transporters of glutamate (VGLU1) and GABA (VIAAT), respectively (Fig. 4A, B, E). Noteworthy, the gold-particles demonstrated tight embedding of the NRXs into the pre-synaptic protein network(s) reflected by the densely packed intra-membrane particles (IMPs), very similar to Cav2.1 (P/Q-type) channels (Fig. 4D). Recapitulation of these experiments following virally-driven knockdown of T178B in the hippocampus largely abolished the preference of NRXs for the synaptic active zones, resulting in almost equal distribution of the NRX-particles between active zone (synaptic) and extra-synaptic localizations (Fig. 4C, E). This re-localization of NRX-particles was observed in virtually all synapses (VGLU1-positive (excitatory) and VGLU1-negative (putatively inhibitory) synapses (all synapses, Fig. 4E)).

Together, these immuno-EM results demonstrated preferred localization of NRXs to the active zone of synaptic boutons as previously observed^52^ and suggested that this preference is due to co-assembly with T178B.

### Integration of the Neurexin-T178-PTPR module into pre- and trans-synaptic protein networks

**Fig. 5:**
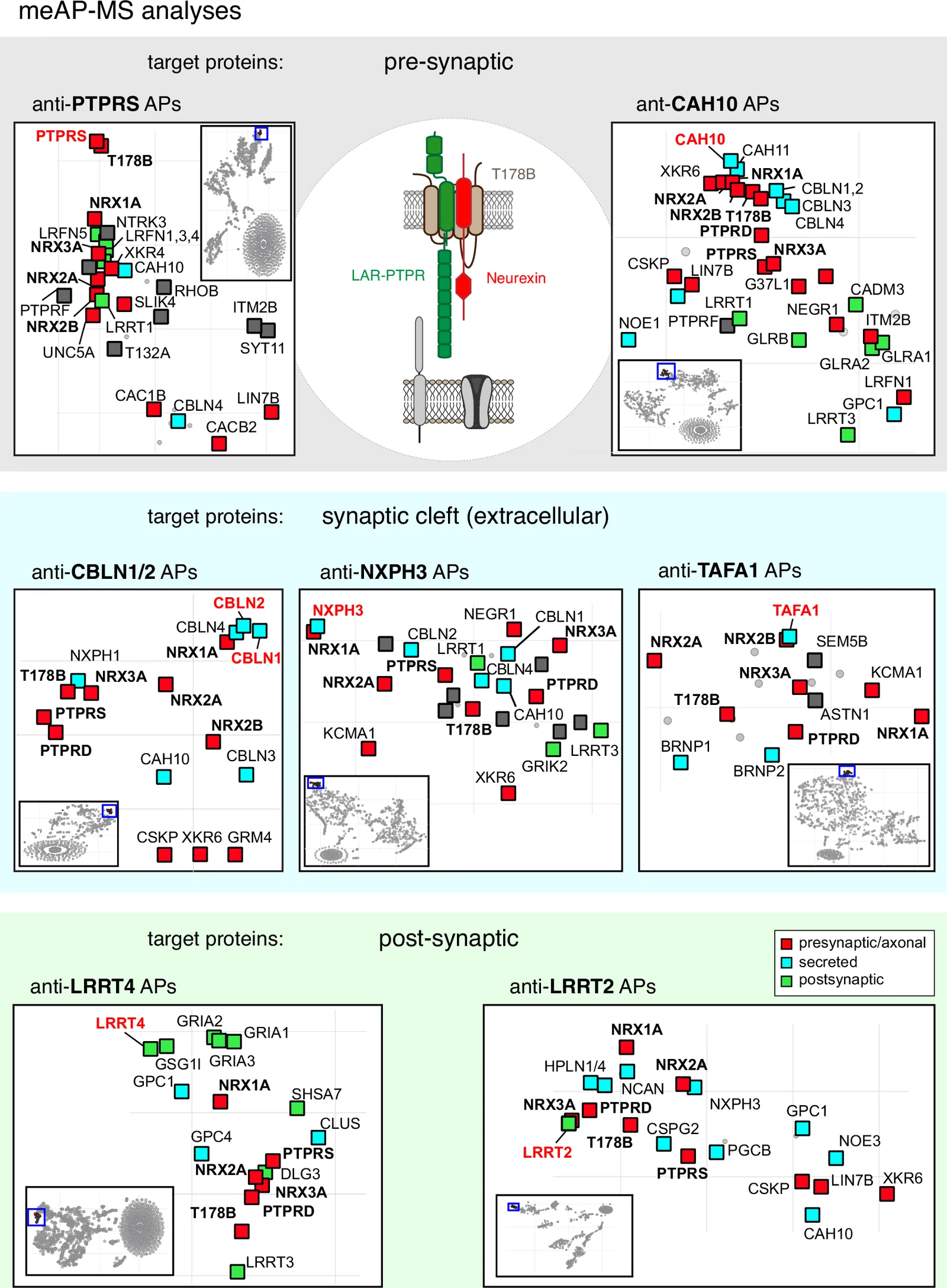
Comprehensive meAP-MS analyses uncover stable formation of trans-synaptic network(s). t-SNE plots of proteins resolved by their tnR-values in APs with multiple ABs specifically targeting the indicated NRX interactors in the pre-synapse (PTPRS, CAH10), the synaptic cleft (CBLN1/2, NXPH3, TAFA1) and the post-synapse (LRRT4). For LRRT2 APs, recombinant LRRT2 was used as a bait. Insets illustrate entire maps of the t-SNEs with all MS-detected proteins (for extension see Supplementary Table 4), enlarged sections (framed in blue) focus on the cluster of proteins in close proximity to the respective target protein. Note that PTPRD/S, T178B and NRXs appear as central elements in all clusters, while the set of proteins specifically and consistently co-purified with the respective target protein is variable. tnR values were determined with IgGs as controls. CBLNs were purified with anti-HA ABs from transgenic HA-Cbln1 and HA-Cbln2 mouse brains, with wild-type brains serving as negative control.

To test whether the ternary NRX-T178-PTPR module selectively engages in specific trans-synaptic interactions or whether it is broadly recruited into assemblies with diverse synaptic partners, we performed a series of reverse meAP-MS-experiments targeting an array of NRX-interactome constituents (Fig. 1, and Table 1). In total, 30 different constituents were selected as targets prioritized to cover (1) pre-synaptic and post-synaptic membranes or the synaptic cleft and (2) the diverse physiological functions annotated in literature and public databases (Fig. 5, upper panel, Table 1). Experimentally, all 64 reverse target meAPs (plus 27 control APs) were performed and rigorously evaluated as described before; in particular, specificity of co-purification (in APs with 1-4 ABs per target) was determined from MS-based tnR-values with target-KOs and/or pre-immunization IgGs as negative controls and an empirically-determined tnR of 0.25 (Supplementary Fig. 3A) as a specificity threshold^35,53^. The complete sets of proteins determined as specific interaction partners of a given target, together with all details related to the respective meAP experiments are summarized in Supplementary Table 4.

Figure 5 illustrates the t-SNE plots of proteins positioned by their tnR-values obtained in multiple APs with six of the 30 selected targets (Fig. 5, central inset): in the pre-synapse (PTPRS and CAH10, a protein covalently bound to NRX1-3^19^), in the synaptic cleft (CBLN1/2, neurexophilin 3 (NXPH3) and TAFA1) and in the post-synaptic membrane (LRRTs 2, 4). Similar to the anti-NRX1-3 APs (Fig. 1C), these t-SNE plots of reverse APs readily delineated well-defined clusters of proteins identified as specific and consistent target-interactors by their close co-localization with the respective target (Fig. 5, insets, blue frame(s)). This finding immediately indicated that all the NRX-associated target proteins were affinity-isolated as constituents of larger assemblies built on robust detergent-resistant protein-protein interactions. Notably, all these assemblies AP-isolated with diverse NRX ligands contained the NRX-T178-PTPR core-module (Fig. 5), highlighting its common incorporation into the examined synaptic protein complexes. Beyond the ternary core-module, the t-SNE-delineated assemblies contained two groups of additional constituents. One group of proteins, such as CBLNs, CAH10/11, or XKR6, was shared among several, but not all, of the isolated assemblies, suggesting their more common integration into NRX-based building block(s). The other group was uniquely detected with individual targets indicating co-assembly with the NRX-based building block(s) in a target-dependent manner. These selectively associated proteins represented a variety of distinct functional classes including proteins involved in cell adhesion, neuronal/axonal growth and development, voltage- and ligand-gated ion channels, G-protein coupled receptors (GPCRs), lipid-modulation, vesicle-fusion/processing or extracellular matrix (ECM).

As an additional finding of major importance, the t-SNE plots showed that pre-synaptic targets (PTPRS and CAH10-NRXs) robustly co-purified integral post-synaptic membrane proteins (including LRRT1,3,4, and the AMPAR-subunits GRIA1-3, GSG1l, SHSA7, and NOE1,3^31^), and vice versa. Accordingly, the NRX-T178-PTPR core-modules establish proteinaceous trans-synaptic bridges that are sufficiently stable for effective quantitative affinity-isolation and that firmly link pre- and post-synaptic membrane proteins (Fig. 5).

Together, these reverse meAPs strongly suggested that the NRX-T178-PTPR module(s) are a hub element of extended protein networks that expand within and across the synaptic membranes and that are built from a conserved core and a molecularly variable periphery.

### The synaptic connectome of the Neurexin-T178-PTPR core module

For more insight into the structure and complexity of these networks, we next explored their strings and (sub)-clusters by detailed analyses of the core module-associated interactomes (Table 1, Supplementary Table 4) and by using the quantitative data on protein abundances for automated cluster-analysis with the UMAP-algorithm^54^ (Methods, Supplementary Fig. 12).

**Fig. 6:**
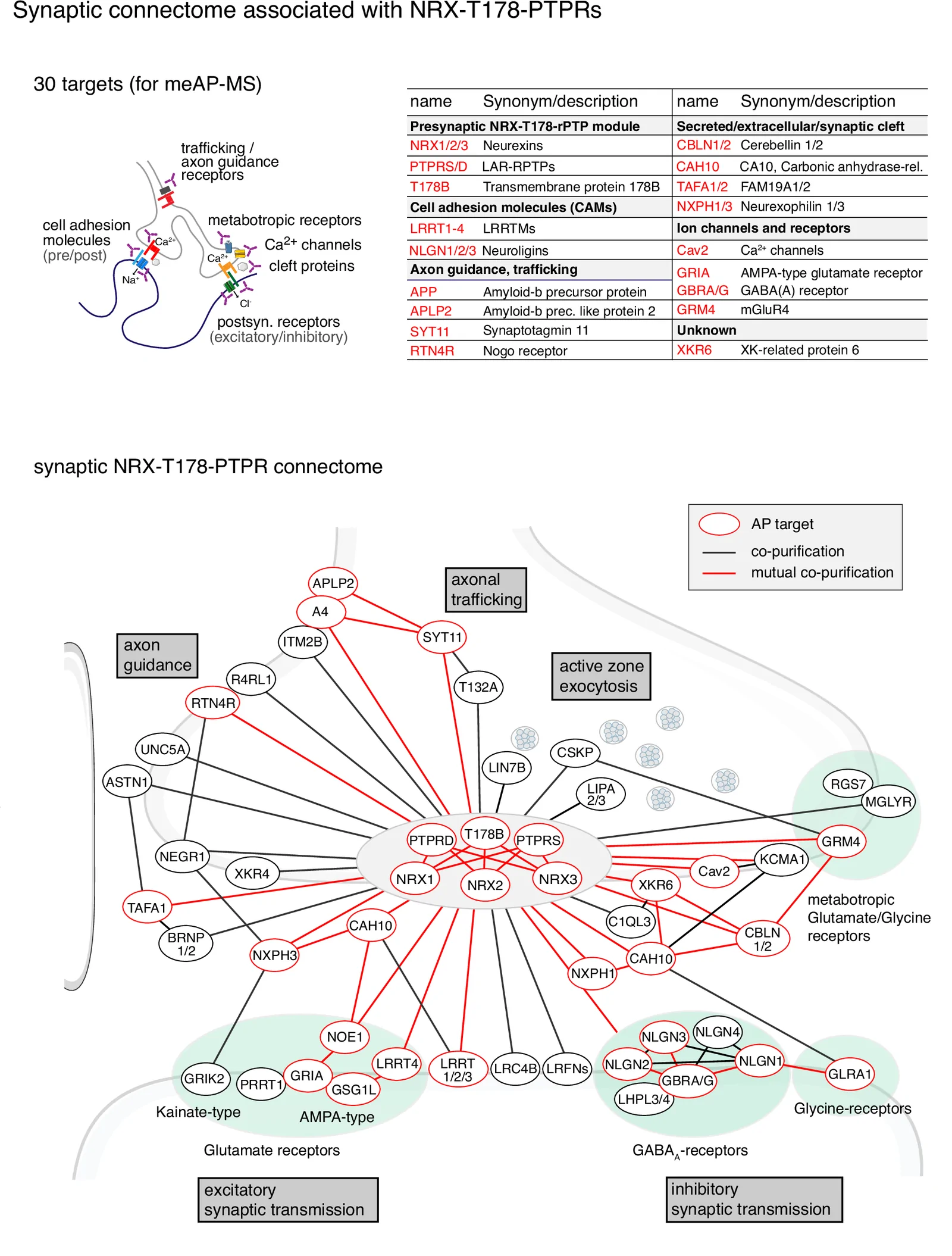
Architecture of NRX-associated protein networks projected onto the synaptic compartment. Graphical illustration of the architecture of NRX-T178-PTPR-associated networks built on robust and specific (co)-purifications in APs against the indicated 30 targets (upper panel) projected onto the synaptic compartment(s). Proteins which were specifically co-purified in individual APs with the respective target proteins (red circles) from brain membrane fractions were connected with lines in black, mutual co-purifications are indicated by red lines. Subunits/components of excitatory glutamate receptors, inhibitory GABA_A_- or glycine receptors and pre-synaptic GPCRs are highlighted by filled circles in green; overlapping circles indicate documented co-assembly of the respective proteins. Note the tight and multi-dented linkage/connection between the pre-synaptic NRX-T178-PTPR module and the post-synapse, as well as with the synaptic cleft. Illustration of the synapse used Servier Medical Art (https://smart.servier.com, licensed under CC BY 4.0 (https://creativecommons.org/licenses/by/4.0/)). An interactive version of Fig. 6 visualizing all connectivities determined for any selected network constituent and providing additional details on AP-data and linkages to the UniProt/SwissProt database is available online (https://phys2.github.io/synaptic-connectome).

Figure 6 projects the resulting multi-protein network onto the synaptic topology and depicts its architecture built on the protein-protein interactions documented by specific and effective co-purifications between individual or groups of constituents (lines in Fig. 6). Thus, the NRX-T178-PTPR module with its variable subunit composition forms the network core which, as an entity, promotes co-assembly with a multitude of constituents in all three sub-compartments of the synapse (Fig. 6 and Supplementary Fig. 12). In the pre-synapse, the core-module robustly co-assembles with the key elements of the active zone (including LIPA2/3, voltage-gated Ca^2+^ channels of the Cav2-family, RIM-binding proteins 1, 2 (RIMB1,2) and BK_Ca_-type K^+^ channels (KCMA1), Supplementary Table 4) and thus recruits the distinct components of the release-machinery into close spatial proximity^27^. Conversely, the core-module, alone or together with secreted constituents (CAH10, NXPH3), associates with excitatory and inhibitory transmitter-receptors or receptor complexes (AMPARs or kainate receptors, and GABA_A_-receptors (GABA_A_Rs) or glycine receptors) in the post-synaptic membrane, thus juxtaposing them to the pre-synaptic release sites. Interestingly, network integration of the two major neurotransmitter-receptors, AMPARs and GABA_A_Rs, is profoundly different: While AMPARs are linked to the core-module through several different strings (NOE1-NRX-CAH10, GSG1l-LRRT4), GABA_A_Rs predominantly rely on the NRX-NLGN binding (Fig. 6).

In addition to these network constituents operating in synaptic transmission, the NRX-T178-PTPR module co-assembles with a variety of constituents that may either serve more specific functions (not present in any type of excitatory/inhibitory synapse) or currently lack functional annotations. These include the metabotropic glutamate receptor GRM4, and the metabotropic glycine receptor (MGLYR/GPR158)-RGS7 complex^55^, as well as several surface receptors previously implicated in neuronal development (and axonal pathfinding) such as RTN4R, R4RL1, ASTN1, NEGR1, the A4/APP-APLP2-ITM2B complex^32^ or T132A, a presumed regulator of integrins and the integrin pathway^56^. Finally, the XKR proteins 4 and 6, either verified (XKR4) or possible (XKR6) lipid scramblases, should be mentioned based on the presumed significance of lipid signaling in (pre-)synaptic physiology^57^. The significance of these more specific network constituents for synaptic organization and function remains to be resolved using the NRX-T178-PTPR module-instructed networks as a guide; for more detailed and ready accessibility, the network illustrated in Fig. 6 is, therefore, also provided as an interactive figure version online (https://phys2.github.io/synaptic-connectome).

## Discussion

Individual CNS synapses contain an array of sCAMs, but it remained enigmatic whether different classes of sCAMs have individual, additive contributions to synapse formation or whether they are tied into functional platforms. In this work, we uncovered that the two major classes of sCAMs with synapse-organizing function assemble into a pre-synaptic core-module interlinked by the T178 proteins. Importantly, this NRX-T178-PTPR module acts as the central hub of solid trans-synaptic protein networks that recruit various classes of signaling entities in the pre-synapse and form tight associations with post-synaptic receptors and adhesion systems.

### Molecular appearance of native synaptic protein networks - technical aspects of the proteomic analyses

For thorough and unbiased analysis of the molecular appearance of synaptic protein complexes in the adult rodent brain, we applied a largely refined workflow of meAP-MS that promotes comprehensiveness and specificity of the pursued interactomes (and interaction partners) by means of its key features^31,32,35,53,58^: (1) use of solubilization conditions that preserve native higher molecular mass complexes of the target (with careful assessment of solubilization via BN-PAGE and MS-analysis^30,31,34,35,45,53,59^, (2) application of multiple verified ABs with distinct epitopes on each target (Fig. 1, Supplementary Tables 1, 5), (3) MS-based protein quantification with a linear range of up to four orders of magnitude and unbiased identification of all proteins, (4) the use of stringent negative controls (knockout input, target-unrelated ABs) and (5) consistency among ABs for determination of interactome constituents (visualized by t-SNE plots of tnR-values, Supplementary Fig. 3)^35^. Related to criteria (4) and (5), it must be noted that our meAP-MS approach aims at high reliability and a minimal number of false-positive interactors, deliberately operating at the cost of false negatives.

As an immediate outcome, this approach identified stoichiometric co-assembly of NRXs1-3 with all three LAR-type PTPRs and the previously un-characterized tetraspanins T178A and T178B (Figs. 1–3). In the ternary complexes that form during early biogenesis in the ER (Fig. 3C, see also below), the two tetraspanins act as structural brackets stabilizing the otherwise rather loose bimolecular interactions between NRX and LAR-PTPRs (Fig. 2). Heparan sulfate moieties, previously implied in cis NRX-PTPR interaction(s)^40,44^, did not impact formation/stability of the NRX-T178-PTPR complexes as evidenced by AP-MS experiments following heparinase treatment (Fig. 2), but profoundly influenced their association with post-synaptic partners in line with previous work^43^.

As a second main outcome, our approach uncovered the NRX-T178-PTPR module(s) as the central hub of trans-synaptic protein networks with exquisite stability and extension as defining features. Thus, the core-module-recruited networks were resistant to detergent-treatment, appeared as large multi-component entities (Figs. 1, 2, 5) and could be effectively (large abundance values of the constituents) and consistently isolated from membrane preparations with multiple ABs targeting a variety of different constituents (visualized in t-SNE plots, Figs. 1, 5, 6). Notably, the isolated network entities uncovered solid trans-synaptic bondings between the pre-synaptic NRX-T178-PTPR module and several integral membrane proteins in the post-synapse. Finally, the architecture of the identified trans-synaptic synaptic network(s) was derived from all entities isolated in extensive meAPs and was built on connectivities between individual constituents exclusively defined by specific, often mutual, co-purification(s) as a proxy for robust protein-protein interactions (Fig. 6).

### Pre-synaptic protein networks–dependence of NRXs-localization on T178B

The embedding/integration of the NRX-T178-PTPR module into protein networks of the pre-synapse was immediately visualized by immuno-EM experiments on freeze fracture replicas of the hippocampal formation: The NRX proteins were found with strong preference (of > 80%) in the active zone areas in the midst of high densities of IMPs representing transmembrane proteins (Fig. 4). This localization preference was independent of the type of synapse, although IMP densities are different for excitatory and inhibitory synapses. Most notably, knockdown of T178B resulted in a redistribution of synaptic NRX proteins in either type of synapse (VGLU1-positive and VGLU1-negative, Fig. 4C, E). This re-distribution of the NRXs into areas with low IMP numbers is meaningful in two ways: First, it parallels the loss of NRX-interaction partners observed in meAP-MS experiments (Fig. 3D), and, second, it provides a plausible explanation for the altered characteristics in synaptic transmission evidenced by the altered paired-pulse ratios at CA3-to-CA1 and MPP-to-GC synapses (Fig. 3E and Supplementary Fig. 11). The distinct effects on release probability upon removal of T178A, B suggest synapse-type specific differences similar to what was observed upon knock-out of presenilin^60,61^.

In addition, the immuno-EM experiments, together with biochemistry/meAPs (Fig. 3D) and patch-clamp recordings (Fig. 3E and Supplementary Fig. 11) clearly demonstrated that reduced amounts of the T178-bracket modifies, but does not abolish synapse-development/formation. The preservation of basic synaptic function(s) is similar to what has been reported for various individual or triple knockouts of NRXs or LAR-PTPRs^9–14^, and is likely due to the substantial amounts of NRXs remaining in the active zone (Fig. 4). Rather, the core-module is of fundamental importance for the organization of pre-synaptic functions and trans-synaptic alignments (see below). In this respect, it will be important to further investigate the precise localization (and function) of the core-module constituents once the respective tools are in hand, in particular specifically and effectively labeling ABs for PTPRs and the two T178 isoforms, as well as the tagged variants of the latter that are currently in progress.

### Significance of the NRX-T178-PTPR module for synapse operation

The NRX-T178-PTPR module-associated synaptic network is made up of an extensive number of constituents located in all three synaptic compartments. Both stability and extension of this network impact the operation of the synapses in several ways, beyond the significance of individual interactions.

Most importantly, the trans-synaptic network provides a solid spatial organization platform where co-operating proteins are placed in close proximity and are thus enabled to functionally interact in a reliable and temporally defined manner. This is directly evident for the co-assembly of key active zone elements (incl. Cav2, and LIPAs) with the NRX-T178-PTPR core in the pre-synaptic membrane and the core-module mediated recruitment of several neurotransmitter receptor complexes in the post-synaptic membrane. The resulting nano-column arrangement^6,62^ juxtaposes release-site(s) and effectors of neurotransmitters and, thus, guarantees fast and reliable synaptic signal transduction. Remarkably, for AMPARs the trans-synaptic connectivity is realized by several molecularly distinct network strings (Fig. 6), pointing to different types of synapses or different states of development and/or maturation. Similar synergism resulting from spatial proximity may be expected for other signaling/regulatory elements identified as co-assembled constituents of the NRX-T178-PTPR network, including the GPCRs GRM4 and MGLYR, the ion channel KCMA1 or the synaptic organizers LRRTs or LRFNs^63–67^, but also the considerable number of proteins that have not yet been related to the two major cell adhesion molecules NRXs and LAR-PTPRs or that lack annotation of resolved primary functions (Fig. 6, Table 1, Supplementary Table 4). This includes the putative scramblase XKR6, as well as proteins T132A, ASTN1, BRNP1, LRC4B or R4RL1.

### Significance of the NRX-T178-PTPR module for synapse assembly

The ternary NRX-T178-PTPR complex identified in this work uncovers an unexpected link between two major pre-synaptic hubs that were considered independent synaptic building blocks in the past^3,14,17,68–70^. This has consequences for interpreting the molecular logic of neuronal synapse assembly. First, the stoichiometric bundling of NRXs and PTPRs substantially expands the molecular interaction codes at the synapse by positioning two structurally distinct classes of adhesion cues into one ternary module. Second, the multivalent extracellular and intracellular interactions with this core-module provide a mechanistic explanation for the extraordinary resilience of the synapse formation process in mammals to genetic perturbation. Notably, individual and even combined deletions of sCAM proteins examined in past studies only partially impair intracellular and extracellular protein interactions with the core-module. Moreover, we discovered that NRX1γ, a short NRX isoform not deleted in the previously reported pan-Neurexin knock-out models^28^, is one of the most abundant neurexin isoforms in the mouse brain. Through the T178 tetraspanin link, this short protein is still sufficient to build NRX-T178-PTPR modules and nucleate pre- and trans-synaptic assemblies.

In aggregate, this work highlights unanticipated avenues for exploring the structural and functional underpinnings of trans-synaptic nanoscale organization and molecular recognition events at neuronal synapses. Moreover, the data provided here serve as a detailed roadmap for analyzing the role(s) of the network as an assembly platform (and the resulting spatial co-assembly/localization), as well as for studying the significance of the newly identified constituents with known and yet unknown function in synapse assembly, operation and dynamics.

## Methods

### Ethics statement

All experiments were approved by the relevant authorities, Regierungspräsidium Freiburg, Germany, under the document TV-G-24/23.

### Generation of knockout/knockin animals

HA-NRX1 knock-in mice^19,71^, NRX3 knock-out (see Supplementary Fig. 5), NRX3 AS5 HA/HA^15^, HA-Nlgn1 knock-in mice^72^ and HA-Cbln1^73^ were generated as described previously. NRX3 knock-outs were generated by inserting a gene-trap after the first exon common to both α- and β-isoforms, using homology-arms PCR-amplified from C57BL/6 genomic BAC clones. The targeting vector, containing an SDA (self-deletion anchor)-flanked neomycin resistance cassette, and a DTA (diphtheria toxin fragment A)-cassette for negative selection, was electroporated into C57BL/6 ES cells (Cyagen). G418-resistant clones were isolated, verified by PCR and Southern Blot and injected into C57Bl/6 albino embryos (Cyagen). Following germline transmission and deletion of the neomycin cassette, heterozygotes were intercrossed to generate homozygous Nrxn3^gt/gt^ offspring and wildtype littermates. HA-Cbln2 knock-in mice were generated following the approach described in ref. ^73^. Briefly, superovulated B6D2F1 female mice (8 weeks old) were mated with B6D2F1 males and zygotes were collected from the oviducts at embryonic day 0.5 and incubated with SpCas9 protein (250 ng/µl; PNA Bio, CA, USA), Cbln2-crRNA (5pmol/µl,5’-CGUAAGGGCUCAGAACGACAGUUUUAGAGCUAUGCUGUUUUG-3’),trans-activating RNA (5pmol/µl; Fasmac, Kanagawa, Japan) and 131-bases single-strand donor DNA oligo(500 ng/µl;5’-TGGCCCTGCTGTTGCTGCTGCTG-CCCGCCTGCTGCCCCGTAAGGGCTCAGTACCCATACGACGTGCCAGACTACGCTAACGACACGGAGCCCATCGTGCTAGAGGGCAAGTGCCTGGTAGTGTGCGATTCC-3’; Integrated DNA Technologies, IA, USA) at 37°C for 15 min. Electroporation was performed using platinum block electrodes (150 mA, 2 pulses, 1msecON+100msecOFF, GE-101, BEX, Tokyo, Japan) connected to CUY21EDIT II (BEX). Zygotes were incubated to the two-cell stage and transferred into oviducts of presudo-pregnant ICR females. To investigate CRISPR/Cas9-mediated mutation in the Cbln2 gene, the genomic DNA was prepared from mouse tales and the genomic regions flanking the gRNA target were amplified by PCR using specific primers: Fwd (5’-ACTTGTTCCCCTTCTACCTGCC-3’) and Rev (5’-GTACGGTTGCTCA-TCTCTGACG-3’). Knockouts for CA10 (*Car10*) were generated by breeding *Car11*^*em1Sud*^ *Car10*^*em1Sud*^*/J* mice (Jackson Laboratories #031998) to C57Bl/6 and subsequently intercrossing *Car10*^*em1Sud*^ heterozygotes to obtain homozygous offspring and wildtype littermates.

Tmem178a/b double conditional mutant mice (*Tmem178a*^*fl/fl*^*; Tmem178b*^*fl/fl*^, shortly T178A,B flox/flox or T178A,B dKO) on a mixed background (129S6/SvEv, C57BL/6, and CD1) were obtained from JAX (RRID:IMSR_JAX:036906; Tmem178b^tm1c(KOMP)Wtsi^ Tmem178^tm1.1Sud^/J). Mice were rederived by embryo transfer and intercrossed to generate homozygous double conditional mutant mice.

T178B-HA knock-in mice were generated by electroporation as described previously^72^ with minor modifications. C57BL/6 J fertilized eggs were incubated with Alt-RTMHiFi SpCas9 Nuclease V3 (250 ng/ml; Integrated DNA Technologies, IA, USA), crRNA (5 pmol/μl, 5’- CCU AAA UCC AGA CCA GAG AAG UUU UAG AGC UAU GCU GUU UUG −3′), trans-activating RNA (5 pmol/µl; Fasmac, Kanagawa, Japan) and a single-stranded oligodeoxynucleotide (500 ng/μl; 5’- CCT CTG TCC AAC CTG TCC CGA GGA CCA ACT GCC CTA AAT CCA GAC CAG AGG GAT CCT ACC CAT ACG ACG TGC CAG ACT ACG CTA ATG GGA CAG TGT GCT AAA AGA CAA ACA AAC CCA TAC CTA TAT ATA TAT A −3’; Integrated DNA Technologies), which harbored a sequence encoding a hemagglutinin (HA) epitope tag and a BamHI site (GGATCC) flanked by homologous arms corresponding to exon 4 of *Tmem18B*, at 37 °C for 15 min before electroporation. Electroporation was performed using platinum block electrodes (100 mA, [3 msec On + 97 msec Off] for 6 pulses; GE-101, BEX, Tokyo, Japan) connected to CUY21EDIT II (BEX). Zygotes were incubated at 37 °C in 5% CO_2_ until they reached the two-cell stage and transferred into oviducts of pseudo-pregnant ICR females. Mice were housed with a 12:12 h light-dark cycle with food and water available ad libitum. The sex for immunohistochemistry was not distinguished.

### Molecular Biology

The cDNAs used were all verified by sequencing. T178B carrying a Myc-Flag Tag at the C-terminus was obtained from Origene (Klon RR216273) and subcloned into pcDNA3.1. PTPRS (GenBank: BC083188.1) was modified with a V5-tag at the C-terminus. HA-tagged NRX1A subcloned into pCAG was obtained from Addgene (#58622). HA-tagged constructs for expression of NRX3A and NRX3A AS5 were as described in^15^.

### Generation of AAV

Adeno-associated virus (AAV) for expression of shRNAs and/or GFP were generated as described^29^. Briefly, oligonucleotides targeting mouse T178B (sh#1: GCCAGGAAGCACAGGGACA; sh#2: ACTTTGAGCTATCACGTTA) and a control oligonucleotide not targeting any gene transcript^29^ (sh-control: 5′-TCGCTTGGGCG-AGAGTAAG) were synthesized as sense-antisense-hairpins and subcloned into transfer vector pAAV-H1/Ubi-eGFP. pAAV-Ubi-GFP was used for sole GFP expression. For AAV production, tsA-201 cells were transfected with transfer, helper and packaging plasmids (AAV-DJ/8, Cell Biolabs, #VPK-400-DJ-8) and cultivated for 5 days. Virus particles were extracted, concentrated by polyethylene glycol 8000 (PEG8000) precipitations and subsequently purified by iodixanol gradient ultracentrifugation. Finally, pure and high-titer aliquots of virus suspensions were obtained by ultrafiltration.

### Biochemistry and Cell Biology

#### Membrane preparation from mouse brain

Whole brains were dissected from adult C57BL/6 WT mice (Charles River) or genetically modified mice. Brains were cut into pieces and homogenized in 10 mM Tris/HCl buffer (pH 7.5) supplemented with 320 mM sucrose, 1.5 mM MgCl_2,_ 1 mM EGTA, 1 mM Iodoacetamide and protease inhibitors (Aprotinin, Leupeptin, Pepstatin A, PMSF) using a loose pestle in a 15 ml dounce tissue grinder. Supernatants of centrifugations (4 min, 1000 × *g*) were collected in tubes for ultracentrifugation. Homogenization procedure was repeated with the remaining tissue remnants in order to increase the yield. Combined supernatants were ultracentrifuged (200,000 ×* g*, for 20 min). The resulting pellet were homogenized with dounce tissue grinder (tight pestle) in hypotonic lysis buffer (10 mM Tris/HCl (pH 7,4) and protease inhibitors) and incubated on ice for 20 minutes. After ultracentrifugation (200,000 × *g*, for 30 min) membrane pellets were resuspended in 8 ml of 0.5 M sucrose / 20 mM Tris/HCl (pH 7.4) and homogenized. Suspension were filled in thin layer ultracentrifugation tubes and underlaid with 12 ml 0.5 M sucrose / 20 mM Tris/HCl (pH 7.4) and 18 ml 1.3 M sucrose / 20 mM Tris/HCl (pH 7.4) and subjected to ultracentrifugation (180,000 × *g*, for 30 min, SW-32, Beckman). The membrane phase between the two sucrose layers was harvested and diluted with 20 mM Tris/HCl (pH 7.4) to wash out sucrose. Ultracentrifugation (200,000 × g, for 30 min) was used to pellet the membranes which were then resuspended in a small volume of 20 mM Tris/HCl (pH 7.4) to reach concentrations about 10 mg/ml. Protein concentration was determined by Bradford assay, and samples were aliquoted and shock-frozen in liquid nitrogen. For proteomic inspection of protein abundances in the membrane fraction from adult mouse brains. Equal amounts of proteins obtained from 4 individually prepared wild-type brains were denatured by adding 10 µl Laemmli-buffer, separated on SDS-PAGE and silver stained. Bands were cut at 45 kDa and 140 kDa, and the three gel pieces were subjected to tryptic digest for nano-LC MS/MS (Fig. 1B, D).

#### Preparation of ER-enriched mouse brain fraction

The isolation of ER-enriched membrane fractions from mouse brain was essentially done as described in^45^. For complexome profiling (csBN-MS) was adjusted as follows. Whole brains of C57BL/6 WT mice were dissected and homogenized in 0.25 M SHE buffer (10 mM HEPES (pH 7.5), 1 mM EDTA, 2 mM PMSF, protease inhibitors see above) first using a loose pestle in a 15 mL dounce tissue grinder and then processed in a cell cracker (8.002 mm ball, 2 passages, 2 rounds per turn). Homogenates were centrifuged at 600 × *g* for 5 min. The supernatant was collected, and the pellet was washed with homogenization buffer. Combined supernatants were centrifuged again (600 × *g* for 10 min). The resulting supernatant was then subjected to ultracentrifugation (200,000 ×* g*, for 30 min). The pellet (P200) was homogenized in hypotonic buffer (5 mM HEPES (pH 7.5) containing 1 mM PMSF and protease inhibitors), left to incubate for 1 h on ice with agitation and subsequently centrifuged at 12,000 × *g*, for 15 min. The supernatant was collected and placed on top of a two layer density gradient (8.3% (0.25 M) and 23% buffered sucrose). After ultracentrifugation (135,000 ×* g* for 1 h), the interface between 8,3% and 23% sucrose layers was harvested, diluted in 20 mM Tris/HCl (pH 7.5), and pelleted (200,000 × *g*, for 25 min). The ER-enriched membrane vesicles were resuspended in 20 mM Tris/HCl (pH 7.5). Protein concentrations were determined by Bradford assay.

#### Covalent coupling of antibodies

For APs, selected ABs were covalently coupled to magnetic Dynabeads ™ Protein A or Dynabeads ™ Protein G. Selected ABs were diluted in PBST (1 μg of AB with 10 μl filtered PBST) and incubated with Dynabeads™ Protein A or G, for 1 h (4 μl Dynabeads per μg of coupled AB). Subsequently, ABs coupled to beads were crosslinked for 30 min by addition of 20 mM Dimethyl pimelimidate dihydrochloride in pre-filtered 100 mM sodium borate, 0.05% Tween (pH 9). Reactions were blocked by adding 200 mM ethanolamine, 0.05% Tween (pH 8). Non-crosslinked ABs were washed off by incubation for 2 × 5 minutes with 100 mM Glycine in 0.05% Tween (pH 3). After 3 x washing steps with PBST coupled ABs were used for APs or stored for short periods at 4 °C.

#### Antibody based affinity-purifications

Protein complexes in plasma membrane and ER enriched fractions from mouse brains were solubilised with ComplexioLyte 91 (CL-91) or ComplexioLyte 47 (CL-47, Logopharm GmbH) supplemented with 1 mM CaCl_2_ (and protease inhibitors (Aprotinin, Leupeptin, Pepstatin A, PMSF)). After clearing by ultracentrifugation (10 min, 125,000 × *g*) solubilisates (1–1.5 mg) were incubated for 2 hours with 10-15 µg ABs pre-coupled to protein A Dynabeads. APs and respective control experiments used to calculate the specificity of interactions (see tnR-ratio calculation below), were performed with identical settings. Target APs were replicated by using different batches of membrane fractions generated from WT mouse brains (as described above). AP experiments with genetically modified brain tissue as source material (NLGN1-HA, CBLN1-HA, CBLN2-HA, Nrx1-HA, NRX3-KO) were done in parallel with controls obtained from age-matched WT littermates. Details of the experimental series are described in the Figure legends and are given in Tables S1–4. The following antibodies were used for APs: *anti-Nrx1-3* (Millipore, #ABN161-I), *anti-Nrx1-3* (AB generation by LifeTein, #RB2987, rabbit, epitope: mouse NRX3A aa 1523-1571); *anti-Nrx1-3* (Synaptic Systems, #175003), *anti-Nrx1-3* (AB generation by AbFrontier, #3-4-rb1, rabbit, epitope: mouse NRX3A aa 1471-1491), *anti-Nrx1* (Synaptic Systems, #175103), *anti-Nrx1* (RnD Systems, #AF4524), *anti-Nrx2* (AB generation by LifeTein, #RB4227, rabbit, epitope: mouse NRX2A aa 266-282), *anti-Nrx2* (AB generation by LifeTein, #RB4226, rabbit, epitope: mouse NRX2A aa 1459-1476), *anti-Nrx2* (AB generation by LifeTein, rabbit, #RB4225, epitope: mouse NRX2A aa 1459-1476), *anti-Nrx2* (Abcam, #ab34245), *anti-Nrx3* (AB generation by AbFrontier, rabbit, #3-1-rb1, epitope: mouse NRX3A aa 1349-1368), *anti-Nrx3* (Synaptic Systems, #175303), *anti-Nrx3* (AB generation by AbFrontier, rabbit, #3-2-rb1, epitope: mouse NRX3A aa 1377-1397), *anti-Nrx3* (RnD Systems, #AF5269), *anti-Nrx3* (AB generation by AbFrontier, rabbit, #3-2-rb2, epitope: mouse NRX3A aa 1377-1397), anti-T178B (AB generation by LifeTein, rabbit, #RB4235, epitope: mouse T178B aa 273-294), anti-T178B (AB generation by AmsBio, rabbit, #RB3967, epitope: mouse T178B aa 273-294), anti-HA (Roche, #11867423001), anti-HA (Thermo Fisher, #26183), anti-PTPRS (RnD Systems, #AF3430), anti-PTPRS (MediMabs, #MM-0020-P), anti-PTPRS (Invitrogen, #PA5-47487), anti-CAH10 (RnD Systems, #MAB2189), anti-CAH10 (raised against recombinant CAH10 (^74^, rabbit, #Hadlai), anti-CAH10 (raised against recombinant CAH10 ^74^, rabbit, #Habeba), anti-CAC1A (FrontierInstitute, #GP-Af810), anti-CAC1E (Alomone, #ACC-006), anti-TAFA1 (RnD Systems, #AF5154), anti-TAFA2 (RnD Systems, #AF4179), anti-NXPH1 (RnD Systems, #AF4208), anti-NXPH3 (RnD Systems, #AF5098), anti-LRRT4 (Neuromab, #75-261), anti-LRRT4 (AbFrontier, rabbit, #C1, raised against mouse LRRT4 aa 548-568), anti-LRRT4 (RnD Systems, #AF5377), anti-A4 (Abgent, #ABIN1741750), anti-A4 (Sigma, #A8717), anti-GBRA1 (Synaptic Systems, #224203), anti-GBRG2 (Synaptic Systems, #224003), anti-RTN4R (RnD Systems, #AF1440), anti-LRRT1 (MerckMillipore, #ABN642), anti-LRRT2 (NeuroMab, #75-364), anti-GRM4 (Abcam, #ab53088), anti-GRM4 (Abcam, #ab184302), anti-GRM4 (Bioworld, #BS1144), anti-NLGN2 (Synaptic Systems, #129511), anti-NLGN3 (NeuroMab, #75-158), anti-Syt11 (Synaptic Systems, #270003), anti-XKR6 (AB generation by AmsBio, rabbit, #RB3961, epitope: mouse XKR6 aa 85-102), anti-XKR6 (AB generation by AmsBio, rabbit, #RB3962, epitope: mouse XKR6 aa 85-102), anti-XKR6 (Signalway Antibody, #42864), IgG (Millipore, #12-370), rabbit pre-immune-sera (LifeTein). Similarly, 15 µg recombinant LRRT2 proteins (RnD Systems, #5589-LR-050) were used as an affinity matrix for protein complex isolations after pre-coupling to protein A Dynabeads.

#### 2D Blue Native/SDS-PAGE analysis of complexes

Protein complexes in mouse brain plasma membrane-enriched fractions^32,45,53^ were solubilized with CL-47 (1 mg membrane protein / 1 ml CL-47 supplemented with 1 mM PMSF, 1 mM Iodoacetamide and a mixture of protease inhibitors (Leupeptin, Pepstatin A, Aprotinin). After clearing by ultracentrifugation (125,000xg, 10 min.), solubilisates were supplemented with 0.05% Coomassie® Brilliant Blue G-250 dye (Carl Roth) and 8% Glycerol. Protein complexes were separated on a 3-15% (w/v) acrylamide linear gradient gel (format 1.6 × 140 × 80 mm), made with 50 mM BisTris (pH 7,5), 50 mM α-capronicacid and 0,01% GDN (Anatrace, #GDN101). Solubilisates (1 ml) were loaded on 3 cm pockets and separated by electrophoresis (180 Volt, 32 mA, 45 min, 600 V, 6 hours) with 15 mM BisTris / 50 mM Tricine / 0.02% Coomassie G250 as cathode and 15 mM BisTris (pH 7.4) as anode buffer. Gel lanes were excised, cut in strips of 0.8 cm, incubated at 37 °C for 20 min in 0.1% SDS (pH 8.8), 100 mM DTT and horizontally placed on top of 10% SDS-PAGE gels. After electrophoretic separation and transfer on a PVDF membrane, proteins were stained with Ruby Blot stain to visualize abundant protein complexes, which were used as internal molecular weight standard for estimation of the apparent molecular sizes in the first dimension. PVDF membranes were cut horizontally at 50 kDa and 125 kDa. The low molecular weight section was immunodecorated with anti-T178B (LifeTein, #RB4235), the 50-125 kDa section with anti-PTPR (Neuromab, #75-194) and the high-molecular area with anti-NRX1-3 (LifeTein, #RB2987). Antibodies were diluted 1:1000 in PBST + 3% BSA. Anti-HRP-conjugated secondary ABs (Abcam) in combination with ECL Prime (GE Healthcare) were used for visualization. Uncropped scans of Western Blots are shown in the Source Data File.

#### Enzymatic removal of heparan sulfates (HS)

Impact of HS on NRX-interactions was assessed by APs after pre-treatment with a heparinase cocktail. For this purpose, plasma-membrane enriched fractions from mouse brain were incubated for 1 h with a mix of Heparinases (2 Units/ml mix of Heparinase I (Sigma-Aldrich, H2519), Heparinase II (Sigma-Aldrich, H6512) and Heparinase III (Sigma-Aldrich, H8891)) diluted in reaction buffer (20 mM Tris/HCl (pH 7.4), 100 mM NaCl, 2 mM CaCl_2_ and a mix of protease inhibitors (Aprotinin, Leupeptin, Pepstatin A). As control, membranes processed in parallel without additions of enzymes were used. After enzymatic treatment, membranes were pelleted by ultracentrifugation (100,000 × *g* for 10 min, 4 °C) and supernatants were removed. Subsequently, membranes were solubilized with CL-91 and processed as described above for APs with a mixture of anti-Nrx1-3 antibodies (anti-Nrx1, #ABN161, Millipore; anti-Nrx1, Synaptic Systems, #175103) and subjected to MS-analysis.

#### Complex reconstitution in tsA cells

tsA201 cells were used for transient co-expression of V5-tagged PTPRS, T178B-Myc-FLAG, NRX1G-HA, NRX1A-HA and NRX3A-HA. Cells were cultivated in high glucose Dulbecco’s Minimal Eagles Medium (DMEM), supplemented with 0.45% glucose, 10% (v/v) FCS, 1% (v/v) Penicillin/Streptomycin and 1% (v/v) HEPES. Two days after PEI (Polysciences)-mediated transfection, cells were washed with PBS and released by cell scrapers in 20 mM Tris/HCl (pH 7,4), 150 mM NaCl, 1 mM EDTA, 1 mM PMSF and protease inhibitors. Cells were centrifuged (150,000xg, 10 min) and lysed in 20 mM Tris/HCl (pH 7,4) by ultrasonication (5s-pulses, output 50%). Membrane vesicles were pelleted by ultracentrifugation (150,000 × *g*, 10 min) and resuspended to homogeneity in 20 mM Tris/HCl (pH 7.4). Protein concentrations of membrane suspensions were determined by Bradford assay and aliquots shock-frozen in liquid nitrogen and stored at −80 °C. Experiments were performed in parallel. For each AP, 0.5 mg membrane fractions were incubated for 30 min in CL-91 (supplemented with 1 mM PMSF, 1 mM Iodoacetamide). After clearing by ultracentrifugation (125,000xg, 10 min), solubilisates were incubated for 2 hours with 3 µg of coupled anti-V5 (V5, BioRad, #MCA1360), anti-HA (Thermo Scientific, #26183), or 40 µl anti-FLAG affinity resin (Millipore, #A2220). Affinity-matrices were briefly washed twice with detergent buffer before elution of bound proteins in 10 µl 1xSDS-buffer without DTT. Aliquots of solubilisates and eluates were run on 10% or 12% SDS-PAGE and proteins transferred onto PVDF membranes. Immunodecorations were done with anti-HA (Thermo Scientific, #26183), anti-V5 (BioRad, #MCA1360) and anti-FLAG (Thermo Scientific, #MA1-91878) antibodies diluted 1:1000 in PBST + 3% BSA. Anti-HRP-conjugated secondary ABs (Abcam) in combination with ECL Prime (GE Healthcare) were used for visualization. Uncropped scans of Western Blots are shown in the Source Data File.

### Mass Spectrometry

#### LC-MS/MS analysis

Proteins were in-gel digested with sequencing grade modified trypsin (Promega GmbH, Walldorf, Germany) alone, or in combination with α-lytic endopeptidase (Sigma, #A6362, for characterization of the N-terminus of T178B). Vacuum-dried peptides were dissolved in 13 µl or 20 µl of 0.5% (v/v) trifluoroacetic acid. Appropriate amounts were loaded onto trap columns (C18 PepMap100, 5 μm particles, Thermo Fisher Scientific GmbH, Dreieich, Germany) with 0.05% trifluoroacetic acid and separated on C18 reversed phase columns (SilicaTip emitters, 75 μm i.d., 8 μm tip, New Objective, Inc, Littleton, USA, manually packed 11 to 12 cm (Orbitrap Elite mass spectrometer) or 21 to 22 cm (QExactive HF-X mass spectrometer) with ReproSil-Pur ODS-3, 3 μm particles, Dr. A. Maisch HPLC GmbH, Ammerbuch-Entringen, Germany; flow rate: 300 nL/min) using UltiMate 3000 RSLCnano HPLC systems (Thermo Fisher Scientific GmbH, Dreieich, Germany). Gradients were built with eluent ˈAˈ (0.5% (v/v) acetic acid in water) and eluent ˈBˈ (0.5% (v/v) acetic acid in 80% (v/v) acetonitrile / 20% (v/v) water): 5 min 3% ˈBˈ, 60 min from 3% ˈBˈ to 30% ˈBˈ, 15 min from 30% ˈBˈ to 99% ˈBˈ, 5 min 99% ˈBˈ, 5 min from 99% ˈBˈ to 3% ˈBˈ, 15 min 3% ˈBˈ (Orbitrap Elite mass spectrometer) or up to 5 min 3% ˈBˈ, 120 min from 3% ˈBˈ to 30% ˈBˈ, 20 min from 30% ˈBˈ to 40% ˈBˈ, 10 min from 40% ˈBˈ to 50% ˈBˈ, 5 min from 50% ˈBˈ to 99% ˈBˈ, 5 min 99% ˈBˈ, 5 min from 99% ˈBˈ to 3% ˈBˈ, 10 min 3% ˈBˈ (QExactive HF-X mass spectrometer). Eluting peptides were electro-sprayed at 2.3 kV (positive polarity) via Nanospray Flex ion sources into an Orbitrap Elite mass spectrometer (CID fragmentation of the 10 most abundant at least doubly charged new precursors per scan cycle) or into a QExactive HF-X mass spectrometer (HCD fragmentation of the 25 most abundant doubly, triply, or quadruply charged new precursors per scan cycle; Figs. 1B, 3B) (all Thermo Fisher Scientific GmbH, Dreieich, Germany) and analyzed with the following major settings: scan range 370 to 1,700 m/z, full MS resolution 240,000, dd-MS2 resolution ˈnormalˈ (ion trap) or 15,000, respectively, maximum dd-MS2 injection time 200 ms or 100 ms, respectively, intensity threshold 2,000 or 40,000, respectively, dynamic exclusion 30 s or 60 s, respectively, isolation width 1.0 m/z. LC-MS/MS RAW files were converted into peak lists (Mascot generic format, mgf) with ProteoWizard msConvert (https://proteowizard.sourceforge.io/). All peak lists were searched twice with Mascot Server 2.6.2 (Matrix Science Ltd, London, UK) against a database containing all mouse, rat, and human entries of the UniProtKB/Swiss-Prot database (release 2024_04). Initially, broad mass tolerances were used. Based on the search results, peak lists were linear shift mass recalibrated using in-house developed software and searched again with narrow mass tolerances for high-resolution peaks (peptide mass tolerance ± 5 ppm; fragment mass tolerance 0.8 Da for Orbitrap Elite peak lists and ± 20 mmu for Q Exactive HF-X peak lists). One missed trypsin cleavage and common variable modifications were accepted. Default significance threshold (*p* < 0.05) and an expected value cut-off of 0.5 were used for displaying search results.

#### MS quantification of proteins

Proteins were evaluated by a label-free quantification procedure^35^. MaxQuant v1.6.3 (http://www.maxquant.org) was used for mass calibration and to extract from FT full scans peptide signal intensities (peak volumes, PVs). The elution times of peptide PVs were aligned (pairwise, Loess regression) and assigned to peptides by matching m/z and elution times acquired by MS/MS identification (tolerances 2-3 ppm / ± 1 min) as described (Bildl et al., 2012). The obtained PV tables (protein-specific peptide signal intensities in all runs) were then further processed to eliminate the influence of PV outliers, false assignments and gaps by exploring the consistency of PV relations within proteins (i.e., protein-specific PV ratios between and within runs). Orthogonal combinations of these relations provided ‘expected PV values’ (EPVs) for each interconnected PV value that served as a measure of accuracy and a weighting factor. In addition, time- and run-dependent detectability thresholds were estimated for each cell in the PV table based on the distribution of measured PVs (3rd percentile within a 3 min elution time window). Qualified PV data in each protein PV matrix was then aggregated to obtain global protein references termed ‘protein reference ridges’ (i.e., vectors that represent the maximum protein coverage of MS/MS-identified and quantified peptides with their ionization efficiencies). In a final step, proteins were quantified by weighted fitting of their measured peptide PVs to their respective reference ridge. In case no (consistent) peptide PVs were identified, an apparent protein detection limit was determined using the detectability thresholds of the three best ionizing peptides fitted to the protein reference ridge. Abundance_norm_spec values (as a measure of molecular abundance) were calculated as described^33^ from the fitted protein ridges. This procedure is comparable to MaxQuant LFQ (^75^; all data is fitted without weighting), but quantification is more robust and accurate, in particular for sparse PV data.

#### Evaluation of proteomic results

Determination of specificity in APs was based on target-normalized abundance ratios (tnRs) of proteins in target APs versus control APs (calculated as described in^35^) together with information on molecular abundance and detection thresholds of proteins. These information were collectively inspected using the BELKI software suite (https://github.com/phys2/belki). For large data sets tnR-values were visualized by t-distributed stochastic neighbor embedding *(*t-SNE, with perplexity of 50, Figs. 1C, 5); comparison of two APs was illustrated as 2D-scatter plot of tnR-values (Fig. 2B). Specific co-purification was defined by two criteria: First, tnR values of ≥0.25 were set as specificity-threshold^35^, unless high-quality ABs or stringent negative controls (target knock-out) allowed reduce of the threshold to 0.2. Second, consistency (of specific co-purification) among APs was applied (Table 1, Supplementary Tables 2–5, Fig. 6), i.e., co-purification of a given protein was dubbed specific if its tnR-value(s) exceeded the specificity-threshold in APs with more than two (or the majority of) target ABs or in replicate experiments. Details about the experiments (ABs, controls), tnR-pairs and specificity-thresholds for each experimental series are indicated in Supplementary Tables 2-5. For evaluation of NRX isoform expression in membrane fractions from adult WT mouse brains, proteins were first separated on SDS-PAGE and sections covering molecular weight ranges from 0-45 kDa, 45-140 kDa, and >140 kDa were cut and individually analyzed by MS (Supplementary Fig. 1). Signals obtained in high molecular weight sections were assigned to NRX1/2/3 alpha. Sequences unique for beta isoforms were exclusively found between 45-140 kDa, and, therefore, the respective fractions were used to determine abundances of NRX1/2/3 beta. Signals obtained in low molecular weight fractions matched with NRX1γ. Abundance_norm_ values were used to calculate relative abundances (Fig. 1B).

For evaluation of whole membrane fractions from wild-type mouse brains or neuronal cultures, total protein amounts were equilibrated to correct for systematic deviations due to technical variations (e.g., sample loading, in-gel digest etc.); normalization factors were smaller than 1.3. Mean values of abundance_norm_spec values determined in *n* = 4 individual mouse brain membrane fractions were calculated and plotted relative to mean abundance_norm_spec values obtained in eluates of four anti-NRX1-3 APs (2 experiments with anti-NRX1-3 (Millipore, #ABN161-I) and (LifeTein, #RB2987) respectively) (Fig. 1D). For direct comparison, all four AP data sets were first normalized to target values.

Similarly, abundance_norm_spec values were used to judge the impact of GPI-modification in NRX3A on protein-protein interactions (Fig. 2C), to reveal changes on NRX1-3 complexes by enzymatic removal of HS-moieties (Fig. 2D), and to monitor alterations in NRX1-3/PTPRS complex formation after sh-RNA mediated knockdown of T178B (Fig. 3D). Abundance ratios providing average fluctuations in association with specific target proteins. Experiments were done in triplicates and values are given as mean ± SEM.

Organellar proteomic analysis used the data published in^45^ re-processed as follows. MS/MS data was searched against the UniProtKB/Swiss-Prot MOUSE ReferenceProteome database (release 2025_11) with the common modifications described above. Quantitative evaluation of the proteins identified with at least three specific peptides was carried out as detailed above. Relative abundance profiles of proteins together with information from Itzhak et al^46^. on general subcellular markers were visualized in BELKI (t-stochastic neighborhood embedding (t-SNE) with perplexity of 40).

For UMAP-analysis of the NRX-T178-PTPR associated networks (Supplementary Fig. 12), abundance_norm_Spec values of 172 proteins, identified as specific interactors in target APs (30 targets outlined in Fig. 6 (except of GRIA) and replicate experiments, indicated in Supplementary Tables 2-5), were used to uncover data structures pointing towards clusters or preferred assemblies. The UMAP^76^ method was used to reduce the dimensionality of the normalized data, leveraging the software provided by McInnes et al. (https://doi.org/10.21105/joss.00861), which is based in the programming language Python^77^. The Euclidean distance was chosen as the metric for clustering the data. The parameter constraining the size of the local neighborhood of a data instance (n_neighbors) was 10. Additionally, the Python library pandas (10.5281/zenodo.3509134) was used for data handling. Visualization of reduced data was done using the Bokeh library (http://www.bokeh.pydata.org.)

#### ER complexome profiling

ER-enriched membrane fractions^45^ were prepared from freshly isolated mouse brains and gently solubilized with CL-47 (salt replaced by 750 mM aminocaproic acid). After ultracentrifugation, 1 mg of solubilized protein was concentrated by ultracentrifugation into a 20%/50% sucrose cushion supplied with 0.125% Coomassie G250 Blue. The sample was then subjected to high-resolution cryo-slicing blue native gel mass spectrometry (csBN-MS) as described recently^34^. Briefly, the BN gel (1-18%) lane was sliced into 359 samples (0.3 mm intervals), each digested with trypsin, and the obtained peptides (dissolved in 0.5% (v/v) trifluoroacetic acid) were measured on a QExactive mass spectrometer coupled to an UltiMate 3000 RSLCnano HPLC system (Thermo Scientific, Germany). After absorption on a C18 PepMap100 precolumn (300 µm i.d. × 5 mm; particle size 5 µm; 0.05% (v/v) trifluoroacetic acid; 5 min 20 µL/min), peptides were eluted with an aqueous-organic gradient (eluent A: 0.5% (v/v) acetic acid; eluent B: 0.5% (v/v) acetic acid in 80% (v/v) acetonitrile) as follows: 5 min 3% B, 120 min from 3% B to 30% B, 20 min from 30% B to 50% B, 10 min from 50% B to 99% B, 5 min 99% B, 5 min from 99% B to 3% B, 10 min 3% B (flow rate 300 nL/min). Separation column was a SilicaTip™ emitter (i.d. 75 µm; tip 8 µm; New Objective, USA) packed manually (23 cm) with ReproSil-Pur 120 ODS-3 (C18; particle size 3 µm; Dr. Maisch HPLC, Germany) from which eluting peptides were directly electrosprayed (2.3 kV; transfer capillary temperature 300 °C) in positive ion mode. MS acquisition parameters were: maximum MS/MS injection time = 400 ms; dynamic exclusion duration = 60 s; minimum signal/intensity threshold = 40,000 (counts), top 15 precursors fragmented; isolation width = 1.4 m/z. Mass spectrometric raw data was processed as described above: mass offset-corrected peak lists were searched with 5 ppm mass tolerance against all mouse, rat, and human entries of the UniProtKB/Swiss-Prot database (release 20181205). Acetyl (Protein N-term), Carbamidomethyl (C), Gln->pyro-Glu (N-term Q), Glu->pyro-Glu (N-term E), Oxidation (M), Phospho (S, T, Y), and Propionamide (C) were chosen as variable modifications (fragment mass tolerance: 20 mmu), maximum one missed tryptic cleavage allowed; expect value cut-off for peptide assignment was set to 0.5. Proteins either representing exogenous contaminations (e.g., keratins, trypsin, IgG chains) or identified by only one specific peptide were not considered. Ion intensity information (peak volumes, PVs) extracted by MaxQuant and assigned to peptides after retention time-alignment of runs (RT tolerance 1 min, m/z tolerance 5 ppm) was used to compute protein abundance profiles as detailed in (Schulte et al., 2023). Slice numbers were converted to apparent molecular mass by fitting of log(MW) of marker complexes versus their observed peak maxima in abundance-mass profiles using a sigmoidal function. Abundance-mass profiles were finally smoothed using a gaussian filter (width set to 1.4).

### Neuronal culture and sh-mediated knockdown

Brains were dissected from E18 rat embryos. Hippocampi and cortices of both hemispheres were dissected and transferred into HBSS buffer. Tissue pieces were trypsinized and briefly washed with HBSS buffer after stopping the enzymatic treatment by adding FCS. Cells were dissociated by trituration with a fire polished Pasteur pipettes. Vital cells were plated at high density (500,000 cells/well) for biochemical experiments or medium density (50,000 cells/well) for immunofluorescence experiments on poly-D-Lysine coated wells. For immunostainings cells were cultivated on coated glass coverslips. Neurobasal medium conditioned by cultivated glia cells was added at the start of the culture and freshly every week. Growing of mitotic cells was blocked by supplementing the medium with 5 µM AraC.

Knockdown of T178B by sh-T178B was induced by adding AAV DJ8 particles carrying sequences for H1 promoter-regulated transcription of sh-T178B (sh#1: GCCAGGAAGCACAGGGACA; sh#2: ACTTTGAGCTATCACGTTA) and GFP, whose expression is controlled by an ubiquitin promoter. Control viruses were driving expression of sole GFP. Successful infection and vitality of cultured neurons were controlled regularly; cultures were maintained 2.5 up to 3 weeks to ensure synapse maturation and a quantitative elimination of T178B. Similarly, mouse dissociated cultures of neocortical cells were prepared from P0 mouse pups of T178A,B flox/flox. Neocortices were dissected in HBSS buffer and dissociated by addition of papain (130 units, Worthington #LK003176) for 30 minutes at 37 °C. 4.5 − 5 × 106 cells were plated per 10 cm dish coated with poly-D-lysine and maintained in neurobasal medium (Gibco #21103-049) containing 2% B27 supplement (Gibco #17504-044), 1% Glutamax (Gibco #35050-038) and 1% penicillin/streptomycin (Sigma #P4333). At DIV3 neurons were infected with AAV2/9 virus carrying hSyn-Cre-2A-GFP or hSyn-GFP and cultivated until DIV17. Cells were released by cell scrapers, centrifuged and cell pellets snap frozen in liquid nitrogen.

Neurons were then used for biochemical experiments. They were homogenized in 10 mM HEPES, 320 mM sucrose (pH 7.4) and protease inhibitors by up and down pipetting. After ultracentrifugation (125,000xg, 20 min.) the cell pellets were homogenized in 20 mM Tris/HCl (pH 7.4) and protein concentrations were determined by Bradford. Equal amounts (10 µg) of proteins from each sample were denatured by adding 10 µl Laemmli-buffer, separated on SDS-PAGE and silver stained. Bands were cut in low and high molecular weight pieces and subjected to tryptic digest for nano-LC MS/MS (Supplementary Fig. 10). For analysis (Fig. 3D) of protein assemblies, neurons were solubilized in CL-91 buffer and NRX and PTPRS complexes were affinity-isolated and processed for MS-analysis as described above. In addition, small aliquots of solubilisates and eluates were run on 10% SDS-PAGE and proteins transferred onto PVDF membranes. Blots were cut at 50 and 125 kDa and then separately immunodecorated with anti-T178B (LifeTein, #RB4235), anti-PTPR (Neuromab, #75-194) and anti-NRX1 (Millipore, ABN161-I). Antibodies were diluted 1:1000 in PBST + 3% BSA. Anti-HRP-conjugated secondary antibodies (Abcam) in combination with ECL Prime (GE Healthcare) were used for visualization. Uncropped scans of Western Blots are shown in the Source Data File.

### Electron microscopy

Immuno-gold labeling of SDS-digested freeze-fracture replicas (SDS-FRL) was carried out as previously described^51^. Briefly, coronal hippocampal sections (120 µm) were cut from brains of two adult male WT mice and an adult male sh-T178B injected mouse which had been transcardially perfused with 0.9% NaCl followed by a fixative containing 1% paraformaldehyde (PFA) and 15% saturated picric acid in 0.1 M phosphate buffer (PB; pH 7.4). Slices were cryoprotected with 30% glycerol in 0.1 M PB overnight (O/N) at 4 °C, then blocks containing the strata oriens, pyramidale and radiatum of CA1 microdissected and frozen with high pressure freezer (HPM 100, Leica, Austria). Frozen samples were fractured at −140 °C, then coated by deposition of carbon (5 nm), platinum/carbon (2 nm) and carbon (18 nm) in a freeze-fracture replica machine (ACE 900, Leica). Replicas were subsequently digested for 18 hours (hrs) at 80 °C in a solution containing 2.5% SDS and 20% sucrose diluted in 15 mM Tris buffer (TB; pH 8.3). Replicas were washed in a washing buffer (0.05% bovine serum albumin (BSA, Roth, Germany) and 0.1% Tween-20, in 50 mM Tris-buffered saline /TBS/), blocked in a solution containing 5% BSA and 0.1% Tween-20 made up in TBS for 1 hr at room temperature (RT). Subsequently, replicas, obtained from WT animals, were incubated with one of the following mixtures of primary ABs: (i) vesicular glutamate transporter 1 (VGLU1, goat (Go), 0.5 µg/ml, Frontier Institute, Hokkaido^78^;) and NRX1-3 (LifeTein, #RB2987, rabbit (Rb), 3 μg/ml, see above), (ii) vesicular GABA transporter (VIAAT, Guinea pig (Gp), 4.5 µg/ml, Frontier Institute, Hokkaido^50^;) and NRX1-3 made up in 50 mM TBS containing 1% BSA and 0.1% Tween-20 (antibody solution) O/N at 15 °C. After washing replicas were reacted with a mixture of 6 nm gold-coupled donkey anti-rabbit IgG and either (i) 18 nm gold-coupled donkey anti-goat IgG or (ii) 18 nm gold-coupled anti-guinea pig IgG secondary antibodies (1:30; Jackson ImmunoResearch Europe, Cambridgeshire, UK) diluted in antibody solution O/N at 15 °C. NRX1-3 single labeling was performed on replicas obtained from T178B knock-down mice. Antibody reaction was completed with 12 nm gold-coupled donkey anti-rabbit IgG secondary antibody (1:30; Jackson ImmunoResearch). All ABs used targeted intracellular epitopes and, therefore, result in labeling of the protoplasmic face (P-face), but not exoplasmic face (E-face). Replicas were subsequently rinsed in TBS, ultrapure water and then mounted on 100-mesh grids.

For quantitative analysis, VGLU1- and VIAAT-positive, as well as single NRX1-3 labeled boutons were imaged at 10,000x magnification under an electron microscope (Jeol JEM 2100 Plus, Jeol, Japan). To differentiate between synaptic and extrasynaptic membrane segments of boutons, active zones of the terminals were identified based on the typical morphology consisting of concave shape of the P-face and the higher density of intramembrane particles (IMPs) compared to the surrounding extrasynaptic membrane areas^79^. Only active zones with clear morphological features were selected for the analysis. Finally, the relative distribution of NRX1-3 particles was calculated in synaptic and extrasynaptic areas of the boutons.

### Immunofluorescence

Mice were transcardially perfused with 4% paraformaldehyde (PFA) in 0.1 M phosphate buffer. Sagittal sections (50 μm thickness) were prepared using a microslicer (DTK-1000, Dosaka EM, Kyoto, Japan). For staining of synaptic proteins, sections were treated with 1 mg/ml pepsin in 0.2 N HCl/PBS for 5 min at 37 °C. Sections were incubated with 10% normal donkey serum, followed by primary ABs overnight, and then Alexa 405, 488, 647(Molecular probe, Thermo Fisher), and Alexa 555 (Abcam, Cambridge, UK)-conjugated species-specific secondary ABs for 2 h. Fluorescence images were taken by confocal microscopy (FV-100, Evident, Tokyo, Japan). The following ABs were used: anti-HA (rat, Roche, # 11867423001, RRID: AB_390918), anti-pan Neurexin (rabbit, Nittobo Medical Co., Ltd, # MSFR104640, RRID: AB_2571817), anti-PSD95 (guinea pig, Nittobo Medical Co., Ltd, MSFR105190, RRID: AB_2571612), anti-guinea pig IgG-Alexa Fluor 488 (donkey, Jackson ImmunoResearch Labs, # 706-545-148, RRID: AB_2340472), anti-rat IgG-Alexa Fluor 555 (donkey, Thermo Fisher Scientific, # A78945, RRID: AB_2910652), anti-rabbit IgG-Cy5 (donkey, Jackson ImmunoResearch Labs, # 711-175-152, RRID: AB_2340607).

### Transcript expression pattern

Transcript levels of individual genes in defined cell-types were calculated using the online software DropViz^80^. For this purpose, all subclusters assigned to a particular cell-type, according to the common name function of DropViz, were combined to a meta-group using the cluster comparison feature of DropViz with default parameter settings. The RNA count of a selected gene per 100,000 unique molecular identifiers in the meta-group was then given as target sum per 100k in a downloadable table containing differentially expressed genes.

### Electrophysiology

#### In-vivo stereotactic Injection

The distinct AAVs were injected into C57BL/6 mice 20–23 days after birth (P20-P23). Animals were anesthetized by injection of a ketamine/dorbene mixture and mounted in a Kopf stereotaxic frame (Tujunga). Virus-containing solution (0.5–1 μl) was injected at a single site into the hippocampus (targeting the CA3 region) by means of a UMP3 controller (WPI, Sarasota) and a nanofil syringe/needle (WPI, Sarasota). Following surgery, pups recovered rapidly by antagonist injection and were returned to their home cage. Recordings were performed 4-5 weeks following virus injection. Animal procedures were in accordance with national and institutional guidelines and approved by the Animal Care Committee Freiburg according to the Tierschutzgesetz (AZ G-21/40).

#### Slice preparation

Transverse 300-μm-thick hippocampal slices were cut from brains of 1- to 2-month-old WT mice, as described^81^. Hippocampal slices were cut in ice-cold, sucrose-containing physiological saline using a commercial vibratome (VT1200S, Leica Microsystems). Slices were incubated at 35 °C, transferred into a recording chamber, and super-fused with physiological saline at room temperature.

#### Patch-clamp recordings in brain slices

Patch pipettes were pulled from borosilicate glass (Hilgenberg; outer diameter, 2 mm; wall thickness, 0.5 mm for somatic recordings). When filled with internal solution, they had resistances of ∼2–4 MΩ post-synaptic pipettes. Patch pipettes were positioned using Kleindiek micromanipulator (Kleindiek Nanotechnik, Reutlingen). A Multiclamp 700B amplifier (Molecular Devices, Sunnydale) was used for recordings. Pipette capacitance of both electrodes was compensated to 70%-90%. Voltage and current signals were filtered at 10 kHz with the built-in low-pass Bessel filter and digitized at 20 kHz using a Digidata 1440 A (Molecular Devices, Sunnydale). pClamp10 software (Molecular Devices, Sunnydale) was used for stimulation and data acquisition. Holding potential of CA1 pyramidal neuronal was −70 mV (to record AMPAR-mediated EPSCs). Synaptic responses were evoked by stimulation at 0.1 Hz with a monopolar glass electrode filled with ACSF in the stratum radiatum of CA1 for CA1 pyramidal neurons. To ensure stable recording, membrane holding current, input resistance, and pipette series resistance were monitored throughout the recording. Statistical significance of differences was assessed by non-parametric Mann–Whitney *U* test (as indicated in the legend of Fig. 3).

#### Solutions

For dissection and storage of slices, a sucrose-containing physiological saline containing 87 mM NaCl, 25 mM NaHCO_3_, 25 mM D-glucose, 75 mM sucrose, 2.5 mM KCl, 1.25 mM NaH_2_PO_4_, 0.5 mM CaCl_2_, and 7 mM MgCl_2_ was used. Slices were superfused with physiological extracellular solution that contained 125 mM NaCl, 25 mM NaHCO_3_, 2.5 mM KCl, 1.25 mM NaH_2_PO_4_, 1 mM MgCl_2_, 2 mM CaCl_2_, and 25 mM glucose (equilibrated with a 95% O_2_/5% CO_2_ gas mixture). Pipettes were filled with a K-methylsulfonate intracellular solution containing 120 mM KMeHSO_3_, 20 mM KCl, 2 mM MgCl_2_, 2 mM Na_2_ATP, 10 mM HEPES, and 0.1 mM EGTA. Membrane potentials are given without correction for liquid junction potentials. Values given throughout the manuscript indicate mean ± SEM or SD. Significance of differences was assessed by a nonparametric Mann–Whitney test.

### Quantification and data analysis

All statistical details are indicated in the figure legends. Data are given as mean ± SEM or SD (as indicated) throughout the manuscript and are analyzed as detailed in the Method section. Significance was assessed by the non-parametric Mann–Whitney *U* test, significance levels are indicated (***, **, * for *p* values < 0.001, 0.01, 0.05, respectively; n.s. is not significant).

### Reporting summary

Further information on research design is available in the Nature Portfolio Reporting Summary linked to this article.

## Supplementary information

Supplementory Information

Reporting Summary

Transparent Peer Review file

## Source data

Source Data

## Data Availability

Mass spectrometry data from AP-MS analyses, membrane proteomics and organellar proteomics have been deposited in PRIDE (PXD054017 and 10.6019/PXD054017). The meAP-MS data reconstituting the network illustrated in Fig. 6 are provided as an interactive version (https://phys2.github.io/synaptic-connectome). All other data reported in this work are available from the corresponding authors upon request. Source data are provided with this paper.
