# Supplementary material for: Ternary Neurexin-T178-PTPR complexes represent a pre-synaptic core-module of neuronal synapse organization
Source: Nat Commun. 2026 Sep 5;17:9509. doi: 10.1038/s41467-026-77377-4 (PMC13546301; doi:10.1038/s41467-026-77377-4)
Supplement: Supplementary file 1 [file 41467_2026_77377_MOESM1_ESM.pdf]

Supplementary Information

Ternary Neurexin-T178-PTPR complexes represent a presynaptic core-module of neuronal synapse organization

Spyros Thivaios, Jochen Schwenk, Aline Brechet, Sami Boudkkazi, Nithya Sethumadhavan, Phil Henneken, Eriko Miur[2], Ayumi Hayashi, Maciej K. Kocylowski, Alexander Haupt, Debora Kaminski, Dietmar Schreiner, Agata Nowacka, Jean-Baptiste van den Broucke, Akos Kulik, Uwe Schulte, Fredrik H. Sterky, Michisuke Yuzaki, Peter Scheiffele and Bernd Fakler

- Supplementary Figures 1 - 12
- Supplemental Methods
- Supplementary Tables 1 - 5

Supplementary Figures

Supplementary Figure 1 (information related to Figure 1)

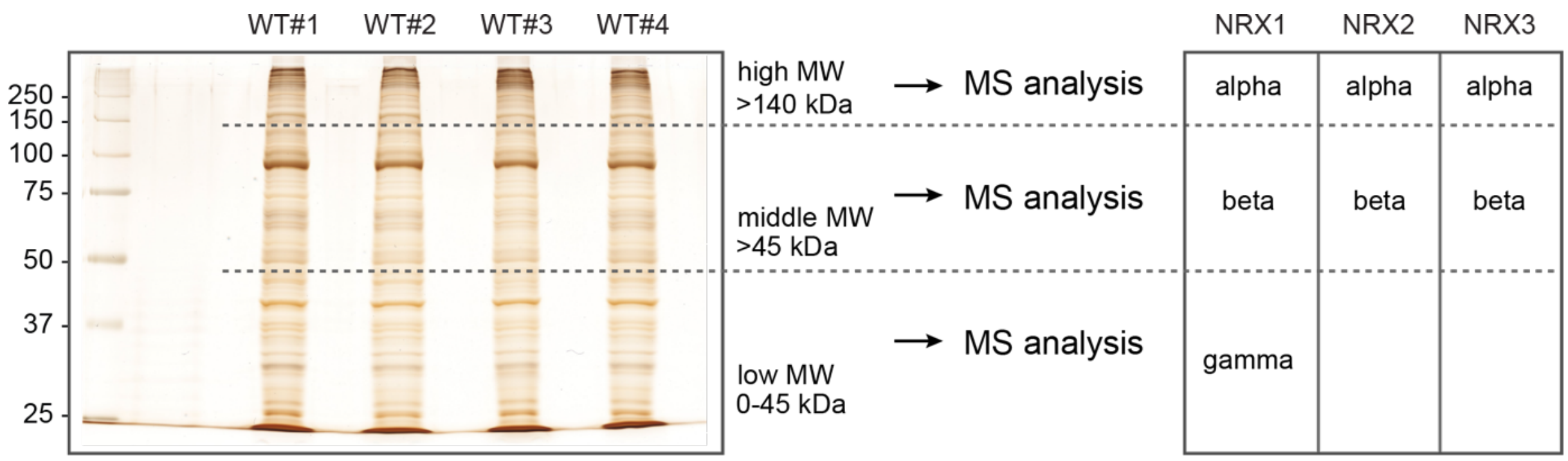

Supplementary Figure 1

Protein abundances of NRX isoforms

Relative abundance of NRX isoforms (alpha, beta, gamma) was determined by quantitative MS in un-solubilized membrane fractions prepared from adult mouse brains. Proteins were separated by denaturing SDS-PAGE, the gel was cut at 45 and 140 kDa and protein abundance was determined in individual sections. All peptides matching the NRX1γ sequence in the 0-45 kDa sections were used to calculate the abundance of this NRX proteoform. Similarly, protein abundances of NRX1β and NRX1α were determined from the middle and high MW sections, respectively.

Supplementary Figure 2

Coverage of primary sequences of NRX isoforms and of T178B detected in pan-NRX1-3 APs

Peptides identified by mass spectrometry are in red; those accessible to, but not identified in MS/MS analyses are in black, and peptides hardly or not accessible to our MS/MS analyses (peptide mass >3 kDa or < 738 Da) are given in grey. Absolute coverage is ‘identified sequences / protein sequence’, relative coverage is ‘identified sequences / MS-accessible protein sequence’.

NRX1A_MOUSE - Neurexin-1

Coverage is **71.0%** absolute, **92.4%** relative

```
0001 MGTALVQRGG CCLLCLSLLL LGCWAELGSG LEFPGAEGQW TRFPKWNACC
0051 ESEMSFQLKT RSARGLVLYF DDEGFCDFLE LILTRGGRLQ LSFSIFCAEP
0101 ATLLADTPVN DGAWHSVRIR RQFRNTTLYI DRAEAKWVEV KSKRRDMTVF
0151 SGLFVGGLPP ELRAAALKLT LASVREREPF KGWIRDVRVN SSQALPVDGG
0201 EVKLDDEPPN SGGGSPCEAG EEGEGGVCLN GGVCSVVDDQ AVCDCSRTGF
0251 RGKDCSQEDN NVEGLAHLMM GDQGKSKGKE EYIATFKGSE YFCYDLSQNP
0301 IQSSSDEITL SFKTLQRNGL MLHTGKSADY VNLALKNGAV SLVINLGSGA
0351 FEALVEPVNG KFNDNAWHDV KVTRNLRQHS GIGHAMVNKL HCSVTISVDG
0401 ILTTTGYTQE DYTMLGSDDF FYVGGSPSTA DLPGSPVSNN FMGCLKEVVY
0451 KNNDVRLELS RLAKQGDPKM KIHGVVAFKC ENVATLDPIT FETPESFISL
0501 PKWNAKKTGS ISFDFRTTEP NGLILFSHGK PRHQKDAKHP QMIKVDFFAI
0551 EMLDGHLYLL LDMGSGTIKI KALQKKVNDG EWYHVDFQRD GRSGTISVNT
0601 LRTPYTAPGE SEILDLDDEL YLGGLPENKA GLVFPTEVWT ALLNYGYVGC
0651 IRDLFIDGQS KDIRQMAEIQ STAGVKPSCS KETAKPCLSN PCKNNGMCRD
0701 GWNRYVCDCS GTGYLGRSCE REATVLSYDG SMFMKIQLPV VMHTEAEDVS
0751 LRFRSQRAYG ILMATTSRDS ADTLRLELDA GRVKLTVNLD CIRINCNSSK
0801 GPETLFAGYN LNDNEWHTVR VVRRGKSLKL TVDDQQAMTG QMAGDHTRLE
0851 FHNIETGIIT ERRYLSSVPS NFIGHLQSLT FNGMAYIDLC KNGDIDYCEL
0901 NARFGFRNII ADPVTFKTKS SYVALATLQA YTSMHLFFQF KTTSLDGLIL
0951 YNSGDGNDFI VVELVKGYLH YVFDLGNGAN LIKGSSNKPL NDNQWHNVMI
1001 SRDTSNLHTV KIDTKITTQI TAGARNLDLK SDLYIGGVAK ETYKSLPKLV
1051 HAKEGFQGCL ASVDLNGRLP DLISDALFCN GQIERGCEGP STTCQEDSCS
1101 NQGVCLQQWD GFSCDCSMTS FSGPLCNDPG TTYIFSKGGG QITYKWPPND
1151 RPSTRADRLA IGFSTVQKEA VLVRVDSSSG LGDYLELHIH QGKIGVKFNV
1201 GTDDIAIEES NAIINDGKYH VVRFTRSGGN ATLQVDSWPV IERYPAGNND
1251 NERLAIARQR IPYRLGRVVD EWLLDKGRQL TIFNSQATII IGGKEQGQPF
1301 QGQLSGLYYN GLKVLNMAAE NDANIAIVGN VRLVGEVPSS MTTESTATAM
1351 QSEMSTSIME TTTTLATSTA RRGKPPTKEP ISQTTDDILV ASAECPSDDE
1401 DIDPCEPSSG GLANPTRVGG REPYPGSAEV IRESSSTTGM VVGIVAAAAL
1451 CILILLYAMY KYRNRDEGSY HVDESRNYIS NSAQSNGAVV KEKQPSSAKS
1501 ANKNKKNKDK EYYV
```

NRX1B_MOUSE - Neurexin-1-beta

Coverage is **65.8%** absolute, **89.3%** relative

```
0001 MYQRMLRCGA DLGSPGGGSG GGAGGRLALI WIVPLTLSGL LGVAWGASSL
0051 GAHHIHHFHG SSKHHSVPIA IYRSPASLRG GHAGTTYIFS KGGGQITYKW
0101 PPNDRPSTRA DRLAIGFSTV QKEAVLVRVD SSSGLGDYLE LHIHQGKIGV
0151 KFNVGTDDIA IEESNAIIND GKYHVVRFTR SGGNATLQVD SWPVIERYPA
0201 GNNDNERLAI ARQRIPYRLG RVVDEWLLDK GRQLTIFNSQ ATIIIGGKEQ
0251 GQPFQGQLSG LYYNGLKVLN MAAENDANIA IVGNVRLVGE VPSSMTTEST
0301 ATAMQSEMST SIMETTTTLA TSTARRGKPP TKEPISQTTD DILVASAECP
0351 SDDEDIDPCE PSSGGLANPT RVGGREPYPG SAEVIRESSS TTGMVVGIVA
0401 AAALCILILL YAMYKYRNRD EGSYHVDESR NYISNSAQSN GAVVKEKQPS
0451 SAKSANKNKK NKDKEYYV
```

NRX1G_MOUSE - Neurexin-1-gamma

Coverage is **57.5%** absolute, **100%** relative

```
0001 MDMRWHCENS QTTDDILVAS AECPSDDEDI DPCEPSSANP TRVGGREPYP
0051 GSAEVIRESS STTGMVVGIV AAAALCILIL LYAMYKYRNR DEGSYHVDES
0101 RNYISNSAQS NGAVVKEKQP SSAKSANKNK KNKDKEYYV
```

NRX2A_MOUSE - Neurexin-2

Coverage is **62.2%** absolute, **84.4%** relative.

```
0001 MALGSRWQPP PQLPPLLLLL ALAAGVRGLE FGGGPGQWAR YARWAGAAST
0051 GELSFSLRTN ATRALLLYLD DGGDCDFLEL LLVDGRLRLR FTLSCAEPAT
0101 LQLDTPVADD RWHMVLLTRD ARRTALAVDG EARAAEVRSK RREMQVASDL
0151 FVGGIPPDVR LSALTLSTVK YEPPFRGLLA NLKLGERPPA LLGSQGLRGA
0201 AADPLCAPAR NPCANGGLCT VLAPGEVGCD CSHTGFGGKF CSEEEHPMEG
0251 PAHLTLNSEV GSLLFSEGGA GRGGAGDVHQ PTKGKEEFVA TFKGNEFFCY
0301 DLSHNPIQSS TDEITLAFRT LQRNGLMLHT GKSADYVNLS LKSGAVWLVI
0351 NLGSGAFEAL VEPVNGKFND NAWHDVRVTR NLRQHAGIGH AMVNKLHYLV
0401 TISVDGILTT TGYTQEDYTM LGSDDFFYIG GSPNTADLPG SPVSNNFMGC
0451 LKDVVYKNND FKLELSRLAK EGDPKMKLQG DLSFRCEDVA ALDPVTFESP
0501 EAFVALPRWS AKRTGSISLD FRTTEPNGLL LFSQGRRAGA GVGSHSSTQR
0551 ADYFAMELLD GYLYLLLDMG SGGIKLRASS RKVNDGEWCH VDFQRDGRKG
0601 SISVNSRSTP FLATGESEVL DLESELYLGG LPEGGRVDLP LPPEVWTAAL
0651 RAGYVGCVRD LFIDGRSRDL RGLAEAQGAV GVAPFCSRET LKQCASAPCR
0701 NGGICREGWN RFVCDCIGTG FLGRVCEREA TVLSYDGSMY MKIMLPTAMH
0751 TEAEDVSLRF MSQRAYGLMM ATTSRESADT LRLELDGGQM RLTVNLDCLR
0801 VGCAPSKGPE TLFAGHKLND NEWHTVRVVR RGKSLQLSVD NVTVEGQMAG
0851 AHTRLEFHNI ETGIMTERRF ISVVPSNFIG HLSGLVFNGQ PYMDQCKDGD
0901 ITYCELNARF GLRAIVADPV TFKSRSSYLA LATLQAYASM HLFFQFKTTA
0951 PDGLLLFNSG NGNDFIVIEL VKGYIHYVFD LGNGPSLMKG NSDKPVNDNQ
1001 WHNVVVSRDP GNVHTLKIDS RTVTQHSNGA RNLDLKGELY IGGLSKNMFS
1051 NLPKLVASRD GFQGCLASVD LNGRLPDLIA DALHRIGQVE RGCDGPSTTC
1101 TEESCANQGV CLQQWDGFTC DCTMTSYGGP VCNDPGTTYI FGKGGALITY
1151 TWPPNDRPST RMDRLAVGFS THQRSAVLVR VDSASGLGDY LQLHIDQGTV
1201 GVIFNVGTDD ITIDEPNAIV SDGKYHVVRF TRSGGNATLQ VDSWPVNERY
1251 PAGNFDNERL AIARQRIPYR LGRVVDEWLL DKGRQLTIFN SQAAIKIGGR
1301 DQGRPFQGQV SGLYYNGLKV LALAAESDPN VRTEGHLRLV GEGPSVLLSA
1351 ETTATTLLAD MATTIMETTT TMATTTTRRG RSPTMRDSTT QNTDDLLVAS
1401 AECPSDDEDL EECEPSTGGE LILPIITEDS LDPPPVATRS PFVPPPPTFY
1451 PFLTGVGATQ DTLPPPAARR PSSGGPCQAE RDDSDCEEPV EASGFASGEV
1501 FDSSLPPTDD EDFYTTFPLV TDRTTLLSPR KPRPNLRTDG ATGAPGVLFA
1551 PSAPAPNLPA GKMNHRDPLQ PLLENPPLGP GVPTAFEPRR PPPLRPGVTS
1601 APGFPRLPTA NPTGPGERGP PGAVEVIRES SSTTGMVVGI VAAAALCILI
1651 LLYAMYKYRN RDEGSYQVDQ SRNYISNSAQ SNGAVVKEKA PAAPKTPSKA
1701 KKNKDKEYYV
```

NRX2B_HUMAN - Neurexin-2-beta

Coverage is **33.6%** absolute, **54.6%** relative.

```
0001 MPPGGSGPGG CPRRPPALAG PLPPPPPPPP PPLLPLLPLL LLLLLGAAEG
0051 ARVSSSLSTT HHVHHFHSKH GTVPIAINRM PFLTRGGHAG TTYIFGKGGA
0101 LITYTWPPND RPSTRMDRLA VGFSTHQRSA VLVRVDSASG LGDYLQLHID
0151 QGTVGVIFNV GTDDITIDEP NAIVSDGKYH VVRFTRSGGN ATLQVDSWPV
0201 NERYPAGNFD NERLAIARQR IPYRLGRVVD EWLLDKGRQL TIFNSQAAIK
0251 IGGRDQGRPF QGQVSGLYYN GLKVLALAAE SDPNVRTEGH LRLVGEGPSV
0301 LLSAETTATT LLADMATTIM ETTTTMATTT TRRGRSPTLR DSTTQNTDDL
0351 LVASAECPSD DEDLEECEPS TGGELILPII TEDSLDPPPV ATRSPFVPPP
0401 PTFYPFLTGV GATQDTLPPP AARRPPSGGP CQAERDDSDC EEPIEASGFA
0451 SGEVFDSSLP PTDDEDFYTT FPLVTDRTTL LSPRKPAPRP NLRTDGATGA
0501 PGVLFAPSAP APNLPAGKMN HRDPLQPLLE NPPLGPGAPT SFEPRRPPPL
0551 RPGVTSAPGF PHLPTANPTG PGERGPPGAV EVIRESSSTT GMVVGIVAAA
0601 ALCILILLYA MYKYRNRDEG SYQVDQSRNY ISNSAQSNGA VVKEKAPAAP
0651 KTPSKAKKNK DKEYYV
```

NRX3A_MOUSE - Neurexin-3

Coverage is **57.1%** absolute, **75.3%** relative.

```
0001 MSFTLHSVFF TLKVSIFLGS LVGLCLGLEF MGLPNQWARY LRWDASTRSD
0051 LSFQFKTNVS TGLLLYLDDG GVCDFLCLSL VDGRVQLRFS MDCAETTVLS
0101 NKQVNDSSWH FLMVSRDRVR TGLVIDGEGQ SGELRPQRPY MDVVSDLFLG
0151 GVPADIRPSA LTLDGVQSMP GFKGLMLDLK YGNSEPRLLG SQSVQLEAEG
0201 PCGERPCENG GICFLLDGHP TCDCSTTGYG GTLCSEDVSQ GPGLSHLMMS
0251 EQAREENVAT FRGSEYLCYD LSQNPIQSSS DEITLSFKTW QRNGLILHTG
0301 KSADYVNLAL KDGAVSLVIN LGSGAFEAIV EPVNGKFNDN AWHDVKVTRN
0351 LRQVTISVDG ILTTTGYTQE DYTMLGSDDF FYVGGSPSTA DLPGSPVSNN
0401 FMGCLKEVVY KNNDIRLELS RLARIGDTKM KIYGEVVFKC ENVATLDPIN
0451 FETPEAYISL PKWNTKRMGS ISFDFRTTEP NGLILFTHGK PQERKDVRSQ
0501 KNTKVDFFAV ELLDGNLYLL LDMGSGTIKV KATQKKANDG EWYHVDIQRD
0551 GRSGTISVNS RRTPFTASGE SEILDLEGDM YLGGLPENRA GLILPTELWT
0601 AMLNYGYVGC IRDLFIDGRS KNIRQLAEMQ NAAGVKSSCS RMSAKQCDSY
0651 PCKNNAVCKD GWNRFICDCT GTGYWGRTCE REASILSYDG SMYMKVIMPM
0701 VMHTEAEDVS FRFMSQRAYG LLVATTSRDS ADTLRLELDG GRVKLMVNLD
0751 CIRINCNSSK GPETLYAGQK LNDNEWHTVR VVRRGKSLKL TVDDDVAEGT
0801 MVGDHTRLEF HNIETGIMTE KRYISVVPSS FIGHLQSLMF NGLLYIDLCK
0851 NGDIDYCELK ARFGLRNIIA DPVTFKTKSS YLTLATLQAY TSMHLFFQFK
0901 TTSADGFILF NSGDGNDFIA VELVKGYIHY VFDLGNGPNV IKGNSDRPLN
0951 DNQWHNVVIT RDSSNTHSLK VDTKVVTQVI NGAKNLDLKG DLYMAGLAQG
1001 MYSNLPKLVA SRDGFQGCLA SVDLNGRLPD LINDALHRSG QIERGCEGPS
1051 TTCQEDSCAN QGVCMQQWEG FTCDCSMTSY SGNQCNDPGA TYIFGKSGGL
1101 ILYTWPANDR PSTRSDRLAV GFSTTVKDGI LVRIDSAPGL GDFLQLHIEQ
1151 GKIGVVFNIG TVDISIKEER TPVNDGKYHV VRFTRNGGNA TLQVDNWPVN
1201 EHYPTGNTDN ERLQMVKQKI PFKYNRPVEE WLQEKGRQLT IFNTQAQIAI
1251 GGKDKGRLFQ GQLSGLYYDG LKVLNMAAEN NPNIKINGSV RLVGEVPSVS
```

1301 GTTQTTSMPP EMSTTVMETT TTMATTTTRK NRSTASIQPT SDDLVSSAEC
1351 SSDDEDFVEC EPSTDKSLST SIFEGGYKAH APKWESKDFR PNKVSETSRT
1401 TTTSLSPELI RFTASSSSGM VPKLPAGKMN NRDLKPQPDI VLLPLPTAYE
1451 LDSTKLKSPL ITSPMFRNVP TANPTEPGIR RVPGASEVIR ESSSTTGMVV
1501 GIVAAAALCI LILLYAMYKY RNRDEGSYQV DETRNYISNS AQSNGTLMKE
1551 KQASSKSGHK KQKNKDKEYY V

NRX3B_MOUSE - Neurexin-3-beta

Coverage is **56.1%** absolute, **78.7%** relative.

0001 MHLRIHPRRS PPRRPAWTLG IWSLFWGCIV SSVWSSSNVA SSSSSSPGSH
0051 SQQEHHFHGS KHHSVPISIY RSPVSLRGGH AGATYIFGKS GGLILYTWPA
0101 NDRPSTRSDR LAVGFSTTVK DGILVRIDSA PGLGDFLQLH IEQGKIGVVF
0151 NIGTVDISIK EERTPVNDGK YHVVRFTRNG GNATLQVDNW PVNEHYPTGN
0201 TDNERLQMVK QKIPFKYNRP VEEWLQEKGR QLTIFNTQAQ IAIGGKDKGR
0251 LFQGQLSGLY YDGLKVLNMA AENNPNIKIN GSVRLVGEVP SVSGTTQTTS
0301 MPPEMSTTVM ETTTTMATTT TRKNRSTASI QPTSDDLVSS AECSSDDEDF
0351 VECEPSTGRS DKSLSTSIFE GGYKAHAPKW ESKDFRPNKV SETSRTTTTS
0401 LSPELIRFTA SSSSGMVPKL PAGKMNNRDL KPQPDIVLLP LPTAYELDST
0451 KLKSPLITSP MFRNVPTANP TEPGIRRVPG ASEVIRESSS TTGMVVGIVA
0501 AAALCILILL YAMYKYRNRD EGSYQVDETR NYISNSAQSN GTLMKEKQAS
0551 SKSGHKKQKN KDKEYYV

T178B_MOUSE (tr|A0A5F8MQ13) – Transmembrane protein 178B

Coverage is **37.8%** absolute, **91.0%** relative

0001 MAAGRLLLYT GLSLALCALG MLAVAICSDH WYETDARKHR DRCKAFNTRR
0051 VDPGFIYNNN NNLPLRASRS RLDRWEGKLL RARNRRQLFA MSPADECSRQ
0101 YNSTNMGLWR KCHRQGFDPE IAALIRKGEI ERCTYIKYHY SSATIPRNLT
0151 FNITKTIRQD EWHALHLRRM TAGFMGMAVA IILFGWIIGV LGCCWDRGLM
0201 QYVAGLLFLM GGTFCIISLC TCVAGINFEL SRYPRYLYGL PDDISHGYGW
0251 SMFCAWGGLG LTLISGFFCT LAPSVQPVPR TNCPKSRPEN GTVC

Supplementary Figure 3 (information related to Figure 1 and others)

A

target-normalized ratio (tnR)

$$tnR = \frac{Log_{10}\left(ratio_{protein} / ratio_{mean,\ background}\right)}{Log_{10}\left(ratio_{target} / ratio_{mean,\ background}\right)}$$

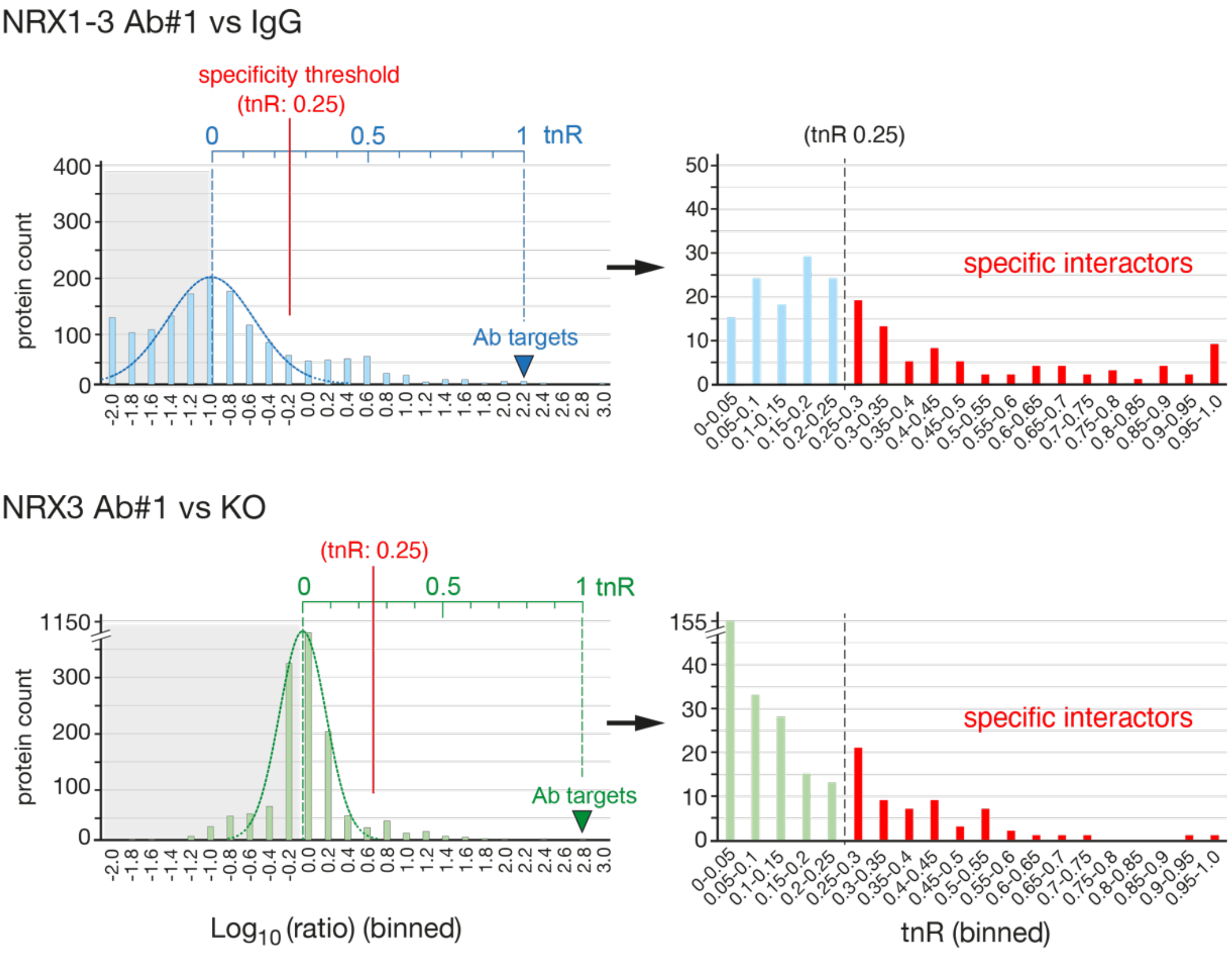

B

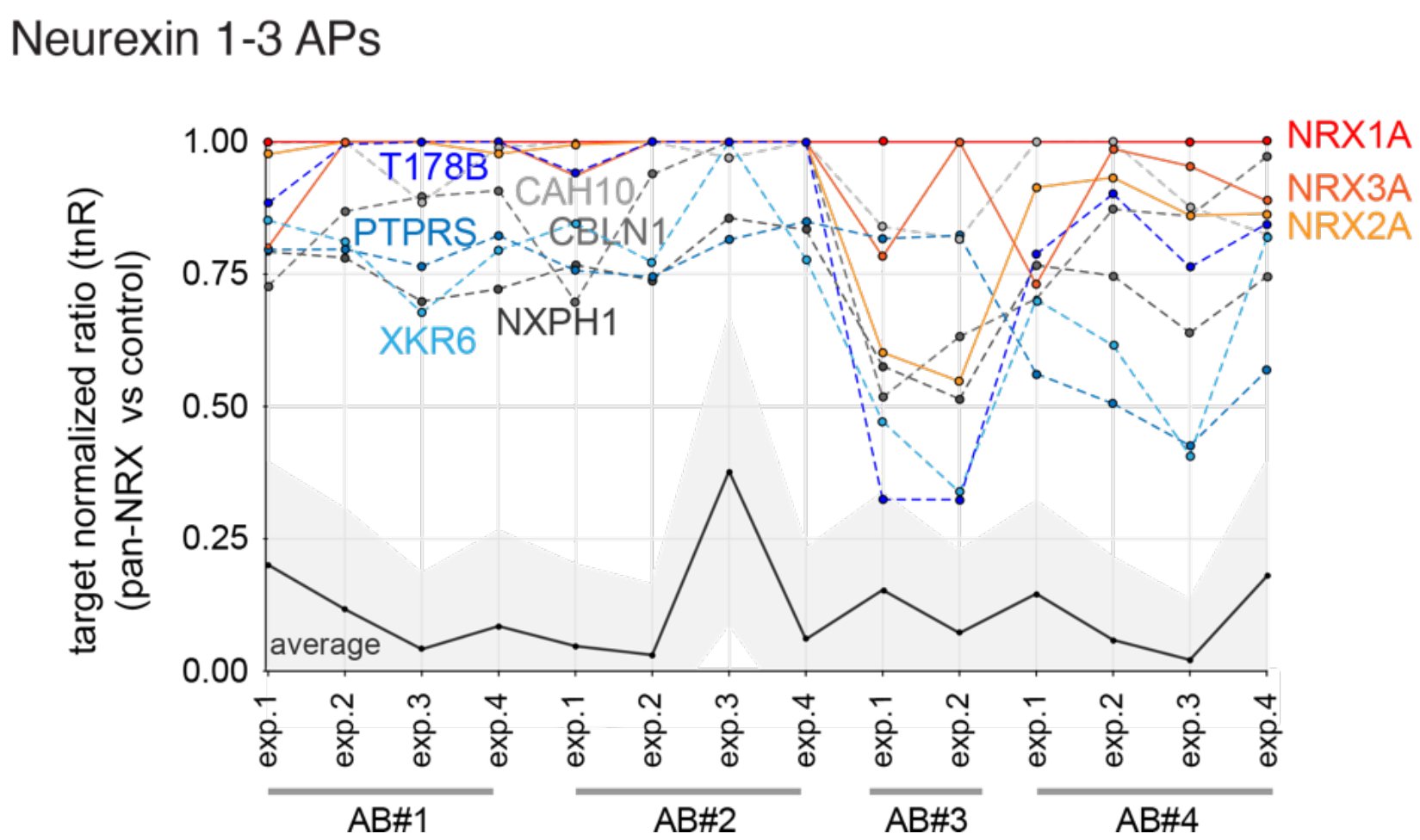

Supplementary Figure 3

Evaluation of NRX APs by target-normalized ratios (tnR)

(A), Top panel, equation for calculating target-normalized ratio values (tnRs, [35]). Middle, example histograms depicting distribution of enrichment factors (i.e. abundance ratios of proteins in an anti-NRX1-3 AP versus an IgG control, log10-

transformed) before (left) and after (right) re-scaling to tnRs with min = 0 derived from the gaussian distribution (dashed curve) of background proteins and max = 1 defined by the enrichment of the primary AP targets (NRX1A and NRX3A). Binning of tnR values as indicated (proteins with tnR = 0 were left out in the right plot for clarity). An empirical specificity threshold of tnR = 0.25 was applied resulting in 83 candidate interaction partners (highlighted red). Lower panel, histograms and tnR-transformation as above for an anti-NRX3A AP from WT versus NRX3A-KO as specificity control yielding 63 candidate interactors. Note that, although target knockouts are generally superior specificity controls (for APs), numbers and distributions of background and co-enriched proteins are qualitatively similar for the NRX ABs used here.

(B), Target-normalized ratio (tnR)-values of selected proteins specifically (co)-purified in 12 anti-NRX1-3 APs with four different anti-NRX1-3 ABs (see also Figure 1C). tnR-values >0.25 indicate specific co-purification (with the target). Average indicates average-tnRs calculated from all MS-identified proteins in the respective AP-eluate, grey area represents SDs of these average-tnRs.

Supplementary Figure 4 (information related to Figure 1 and others)

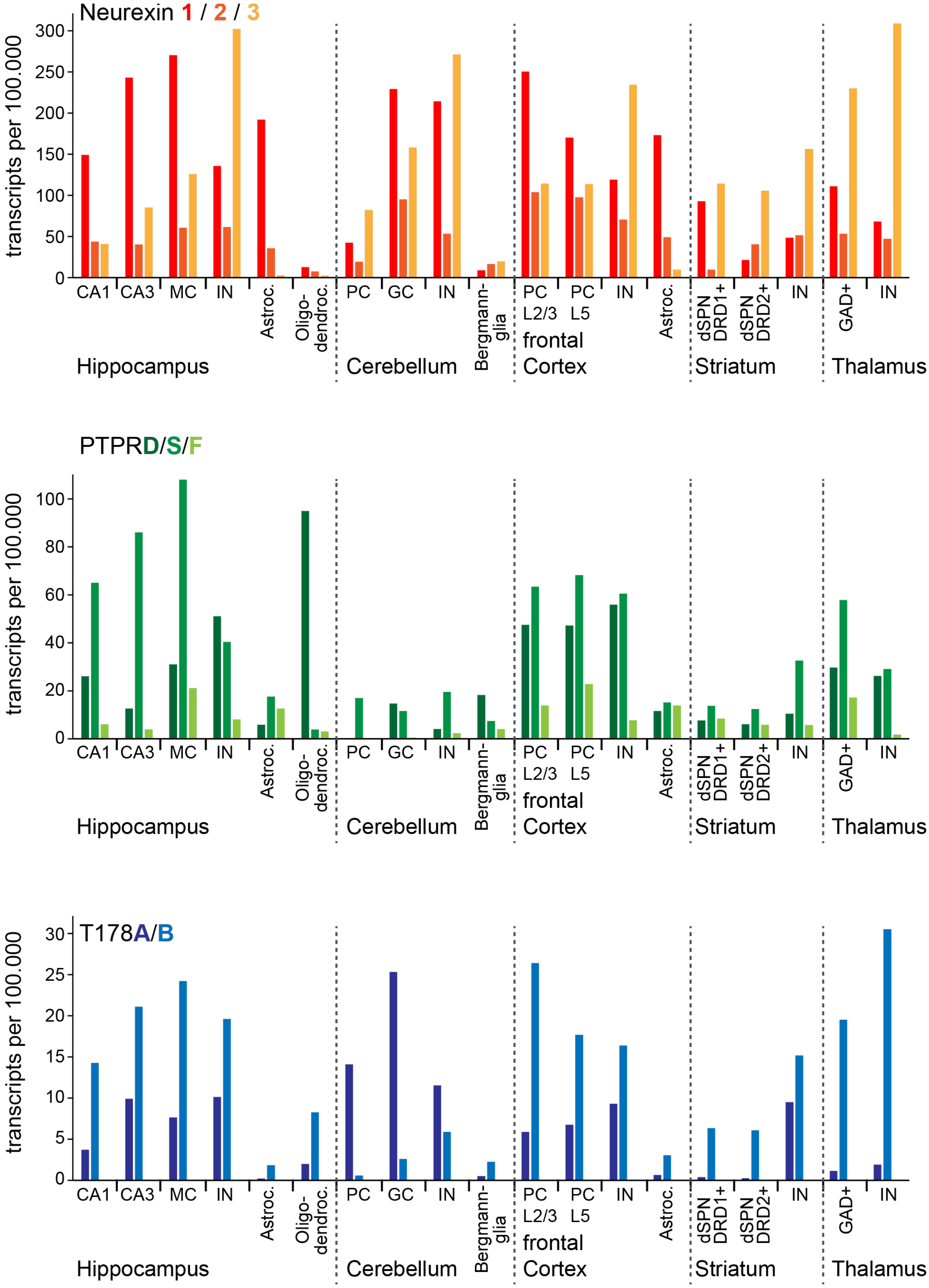

Supplementary Figure 4

Expression pattern of NRX1-3, T178A/B and PTPRD/S/F in mouse brain

Transcriptome data were extracted from the DropViz data base [81]. Number of RNA transcripts per 100,000 unique molecular identifier(s) in the indicated brain regions

and meta-groups according to DropViz single-cell transcriptomics data. Abbreviations are, as follows: CA1, CA1 pyramidal cells; CA3, CA3 pyramidal cells; DG, dentate gyrus; MC, mossy cells; IN, interneurons; GC, granule cells; PC, pyramidal cells; DRD1/2, dopamine receptors D1/2; GAD, glutamic acid decarboxylase.
Note ubiquitous expression all of core-module constituents in both neurons and glia and distinct patterns for T178A (predominantly found in cerebellum).

Supplementary Figure 5 (information related to Figure 2)

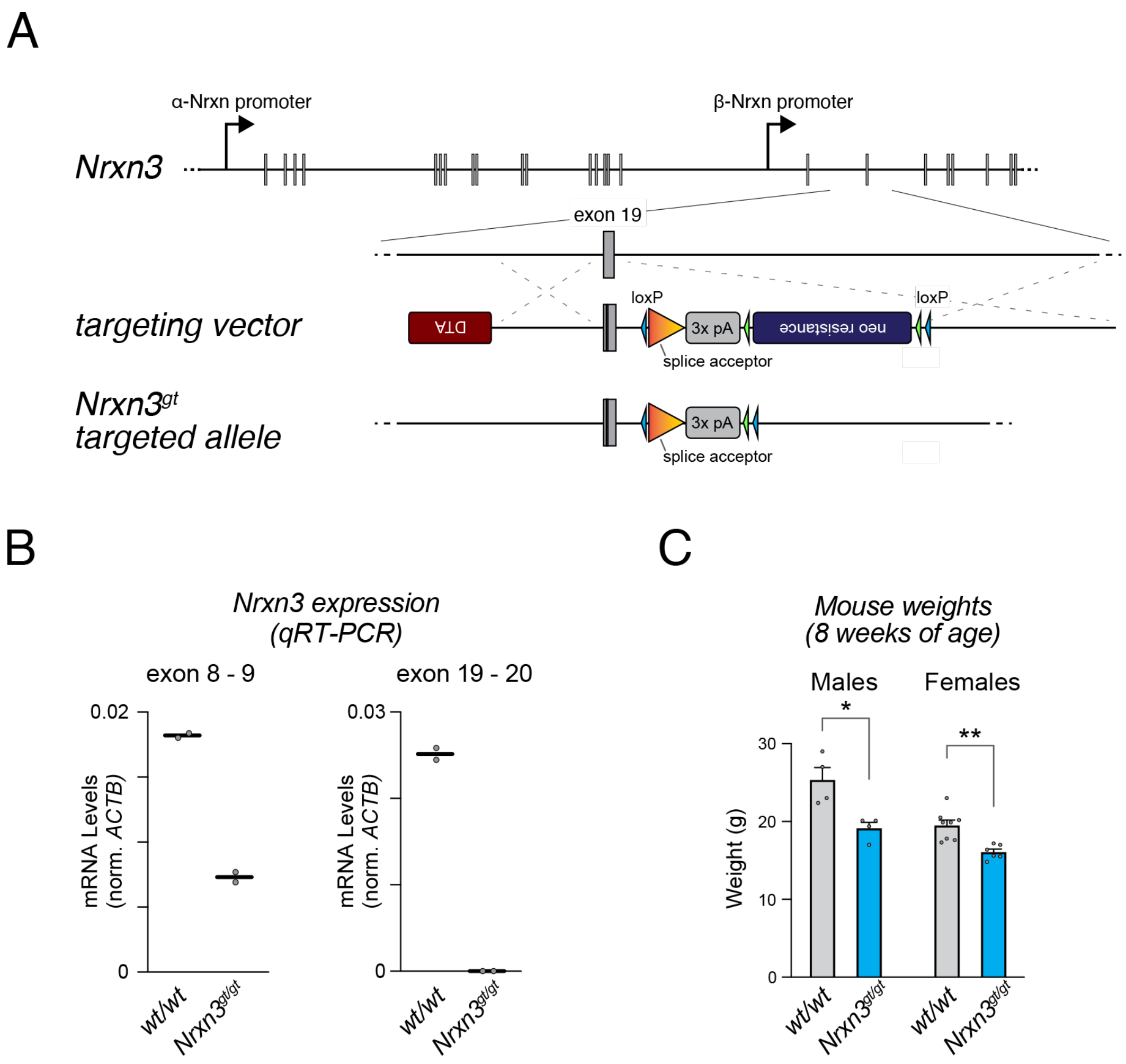

Supplementary Figure 5

Design and validation of NRX3 KO mice

(A), Design of the targeting vector for gene-trap mutagenesis of NRX3 in mice. The gene-trap construct was introduced after the first exon common to both α- and β-isoforms. (B), Gene-trap insertion result in loss of mRNA. Levels of Nrxn3 transcripts before and after the targeted intron, respectively, monitored by qRT-PCR. **c**, Mouse weight at 8 weeks of age. *, $p < 0.05$ and **, $p < 0.01$ by two-tailed students t-test.

Supplementary Figure 6 (information related to Figure 2)

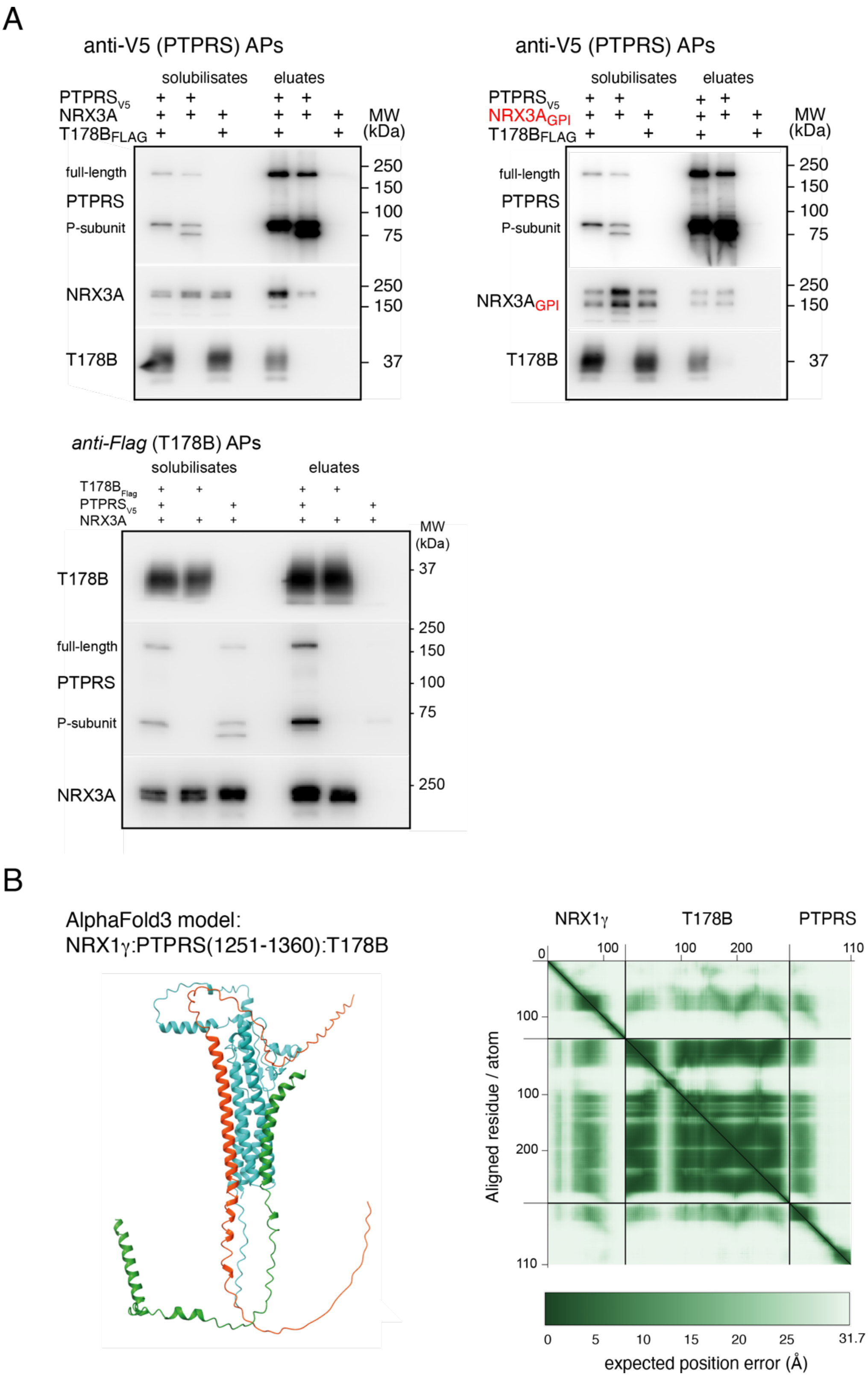

Supplementary Figure 6

Reconstitution of NRX3A, T178B and PTPRS complexes

(A), Upper panel, Western-probed SDS-PAGE separations of input and eluates of APs with anti-V5 ABs from tsA201 cells transiently expressing V5-tagged PTPRs,

FLAG-tagged T178B and either NRX3A or a splice variant of NRX3A lacking the transmembrane domain and attached to the membrane via a GPI-anchor. Note that the presence of T178B appears critical for stable complex formation between PTPRS and NRX3A (similar as NRX1A, **Figure 2E**). In contrast, binding of NRX3A-GPI to PTPRS is weak and independent of the presence of T178B. Lower panel, Western-probed SDS-PAGE separations of input and eluates of anti-FLAG APs from tsA201 cells transiently expressing FLAG-tagged T178B, V5-tagged PTPRS and NRX3A. Note (i) the critical importance of T178B for stable complex formation of PTPRS with NRX3A, and (ii) robust interaction of T178B and NRX3A.

(B), Prediction of 3D-structural arrangement of the membrane-close region of the core-module composed of NRX1γ (mouse, aa 1-139, full length), PTPRS (mouse, aa 1251-1360) and T178B (human, 1-294, full length) by AlphaFold3. Predicted aligned errors (PAE) provide estimates for the confidence of the correct position of two residues in the structure. Low PAE values indicating higher-confidence levels (colored green) were only observed in the transmembrane regions. The predicted template modeling (pTM) score is 0.52 and ipTM score 0.26.

Supplementary Figure 7 (information related to Figure 3)

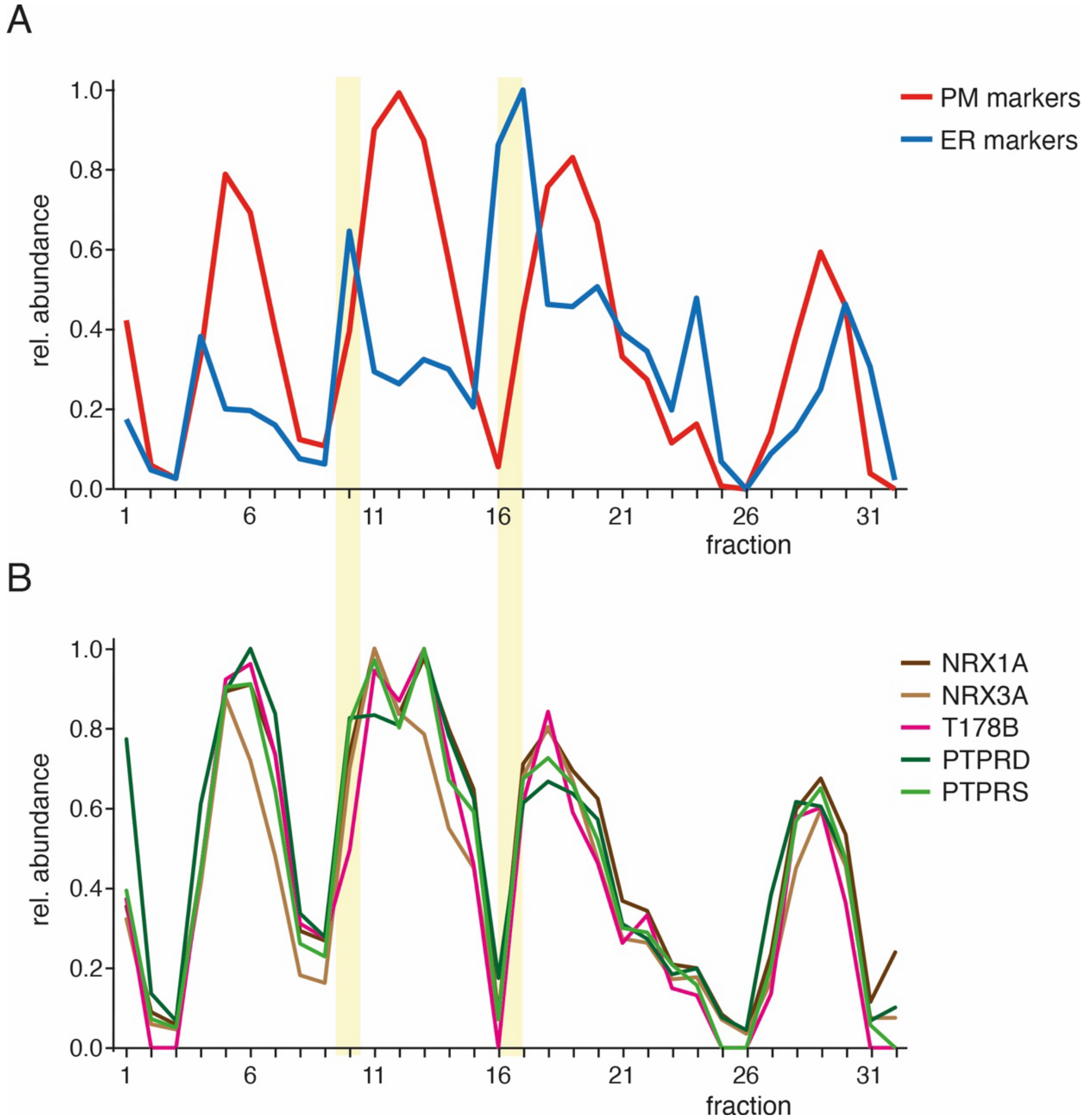

Supplementary Figure 7

Distribution of the core-constituents across subcellular fractions of the rodent brain

(A), Relative abundances determined for marker proteins of plasma membrane (red) or ER (blue) across the 32 subcellular fractions used for organellar proteomics (**Figure 3A**). Data are medians of either type of marker (PM: AT1A3, AT1B1, NPTN, AT2B2, GTR3, CD47, CD59, BASI; ER: PDIA3, AT2A2, CALX, PGRC1, CALR, VAPA, TECR, SAC1).

(B) Relative abundances determined for NRX1A, NRX3A, T178B, PTPRD and PTPRS across the 32 subcellular fractions in (A). Note (i) the close co-distribution of all five core-constituents over all subcellular fractions, in line with their quantitative association found in Figure 1D, and (ii) their strong correlation with plasma membrane markers, shown in Figure 3A. Intensities in fractions 10, 16 and 17 (shaded yellow) that discriminate ER marker proteins from PM markers suggest that a minor portion (<10%) of the ternary core complexes localize to the ER.

Supplementary Figure 8 (information related to Figure 3)

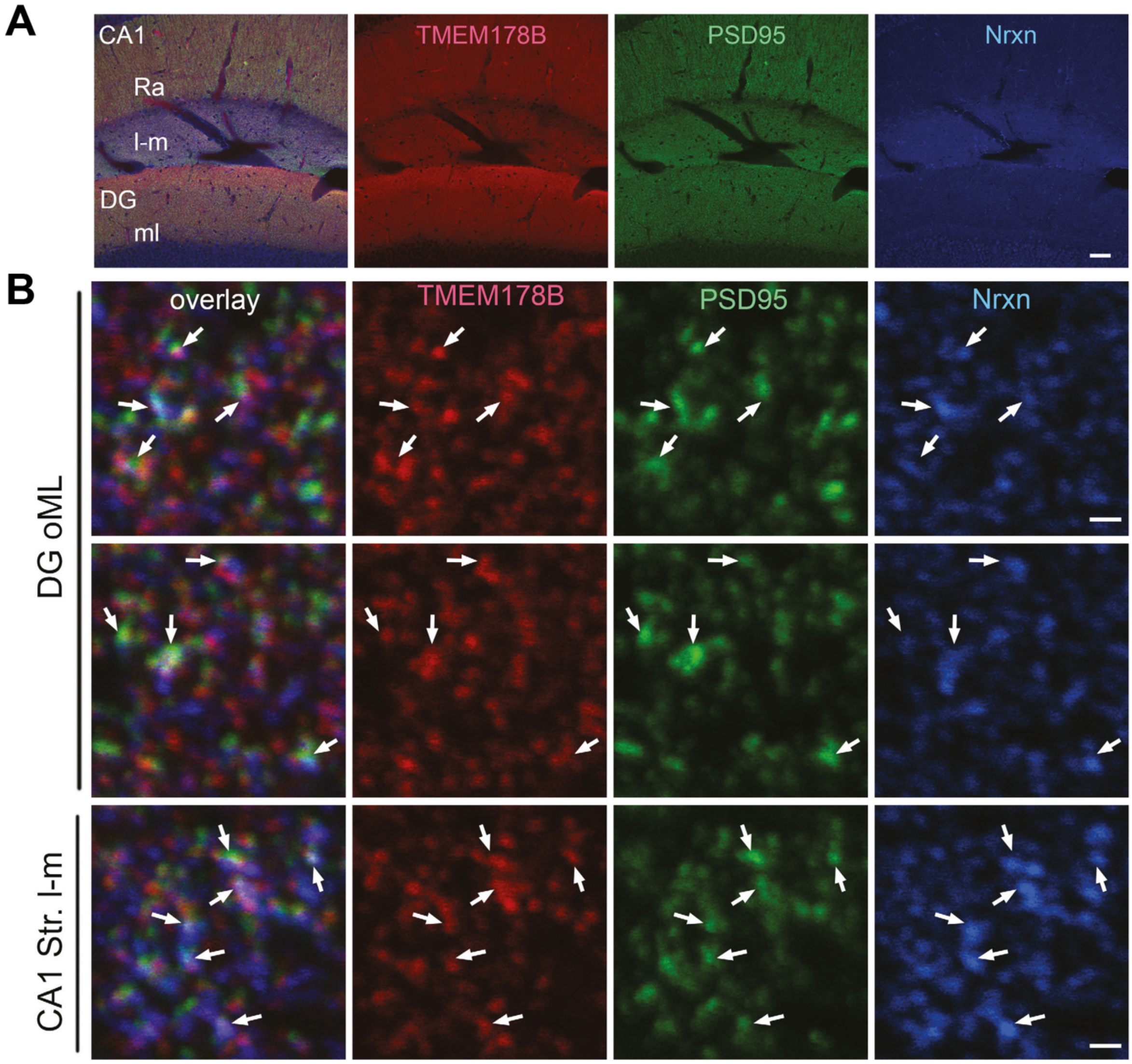

Supplementary Figure 8

Localization of T178B to synapses together with NRX1-3 and PSD95/DLG4 in the hippocampus of adult mice

(A), Low-magnification view of the hippocampus of heterozygous T178B-HA mice showing that T178B (red), PSD95, and NRX are expressed in the stratum radiatum (Ra) and stratum lacunosum-moleculare (l-m) of the CA1 region, as well as in the molecular layer (ml) of the dentate gyrus (DG). Scale bar, 50 µm.

(B), High-magnification views of the outer molecular layer of the dentate gyrus (DG oML) and the stratum lacunosum-moleculare (l-m) of CA1. Arrows indicate co-localization of T178B, PSD95, and NRX. Scale bar, 1 µm.

Supplementary Figure 9 (information related to Figure 3)

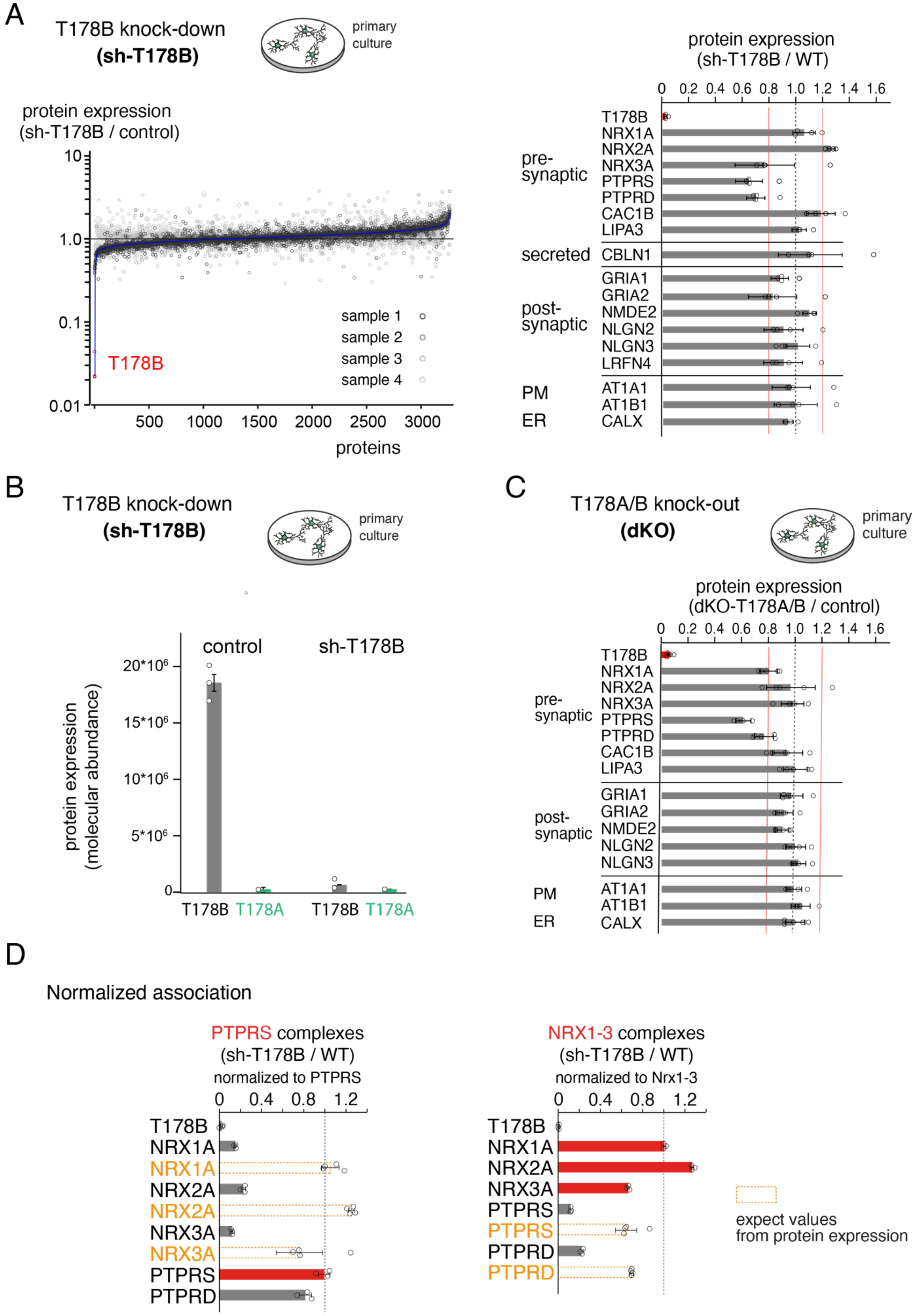

Supplementary Figure 9

Proteome-wide assessment of the specificity of sh-T178B and lack of compensatory upregulation of T178A

(A), Right panel, Alterations in abundance for proteins in membranes from primary neuronal cultures infected with AAV driving expression of sh-T178B relative to infections with AAV control visualized as ratio plot. The fold-change in protein abundance determined by quantitative MS in each of the four independent samples are plotted for 3350 identified proteins. Blue line indicates the median ratio. Note efficiency and specificity of the sh-mediated knockdown of the target protein T178B, which was reduced by ~95%. Left panel, Abundance ratios of selected proteins determined in these neuronal cultures; data are median ± SD. Note marked alteration in the expression of distinct synaptic proteins by sh-T178B. Abundances of PTPRD and S were decreased by ~30-40%, while essential subunits of NMDA receptors (NMDE2), Na/K-ATPase (AT1A1/AT1B1) and Calnexin (CALX) remained unchanged.

(B), MS-determined protein amounts of T178B and T178A in cultures infected with sh-T178B or control virus. Note that sh-T178B selectively reduced its target without any compensatory effect on T178A.

(C), Cre-recombinase mediated removal of T178A, B in cultured neurons from T178A/B flox/flox mice reduced expression of PTPRS and PTPRD, similar to sh-mediated knockdown of T178B. Abundance ratios were plotted as mean ± SD of 5 individual cultures.

(D), Alterations of PTPR and NRX complexes after knockdown of T178B (additional information to **Figure 3D**). Abundance values were target-normalized to visualize the altered degrees of association. Data are mean ± SD of the three anti-PTPRS and anti-NRX1-3 APs in Figure 3D. Values for the AP targets are marked in red. Note that reduced associations are not due to the altered protein expression (see A, right panel), but are over-proportional.

Supplementary Figure 10 (information related to Figure 3)

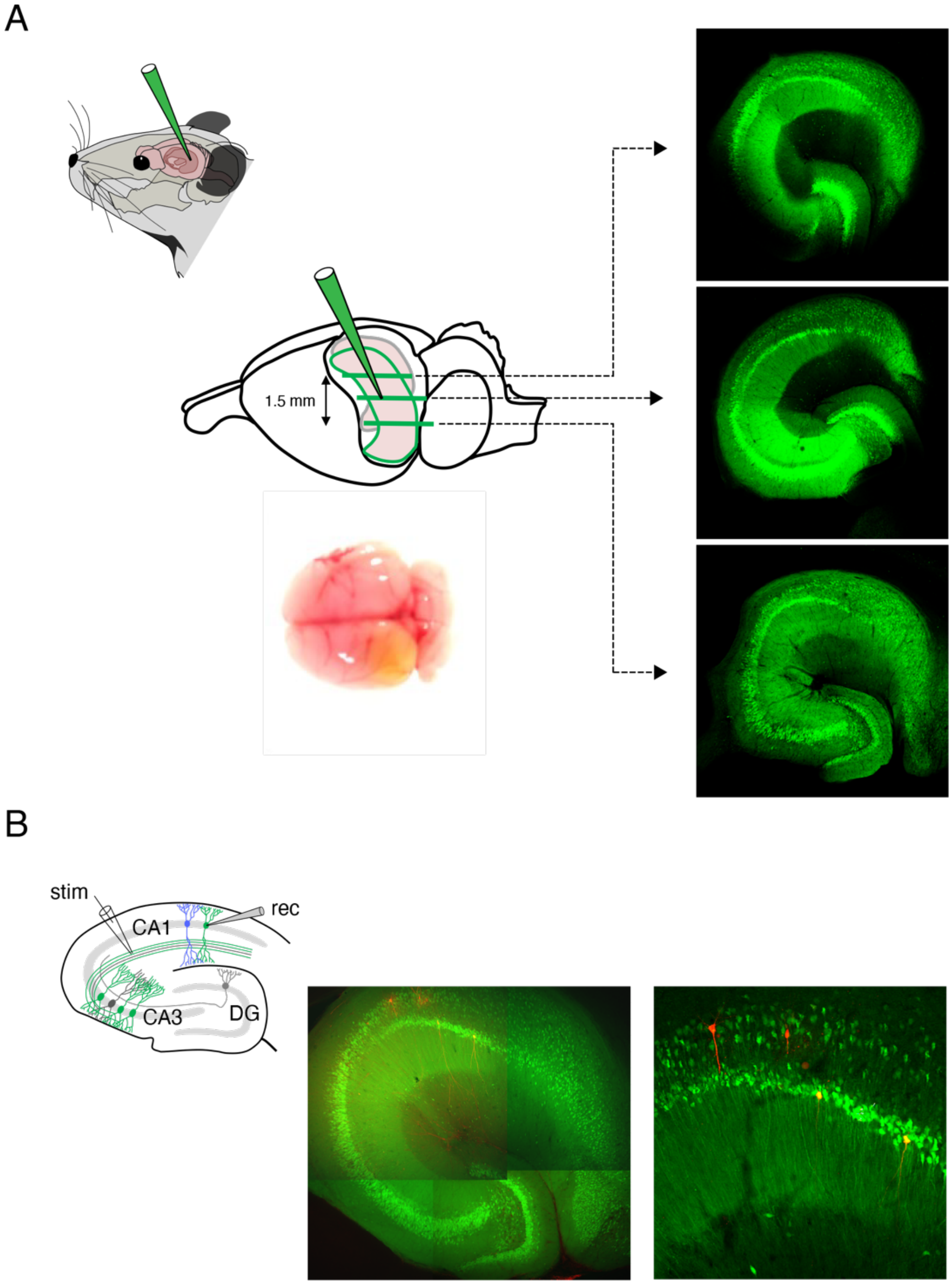

Supplementary Figure 10

Stereotactic injection of AAV expressing sh-T178B into hippocampal CA3 regions and somatic recordings in CA1 pyramidal cells of adult mice

(A), Scheme depicting stereotactic injection into the hippocampal CA3 region and confocal images showing GFP-fluorescence in three brain slices to document spreading and transfection of cells by the AAV particles; GFP-expression is driven by the ubiquitin promotor (see generation of AAV-virus in Methods). Inset: Image showing unilateral injection into the left hemisphere.

(B), Scheme illustrating slice-recordings of CA1 PCs in whole cell configuration and confocal images showing four CA1 PCs filled with biocytin (red fluorescence)

through the patch-pipette during recordings. Note two of these cells were infected by the AAV (yellow fluorescence), two cells were non-infected (red fluorescence).

Supplementary Figure 11 (information related to Figure 3)

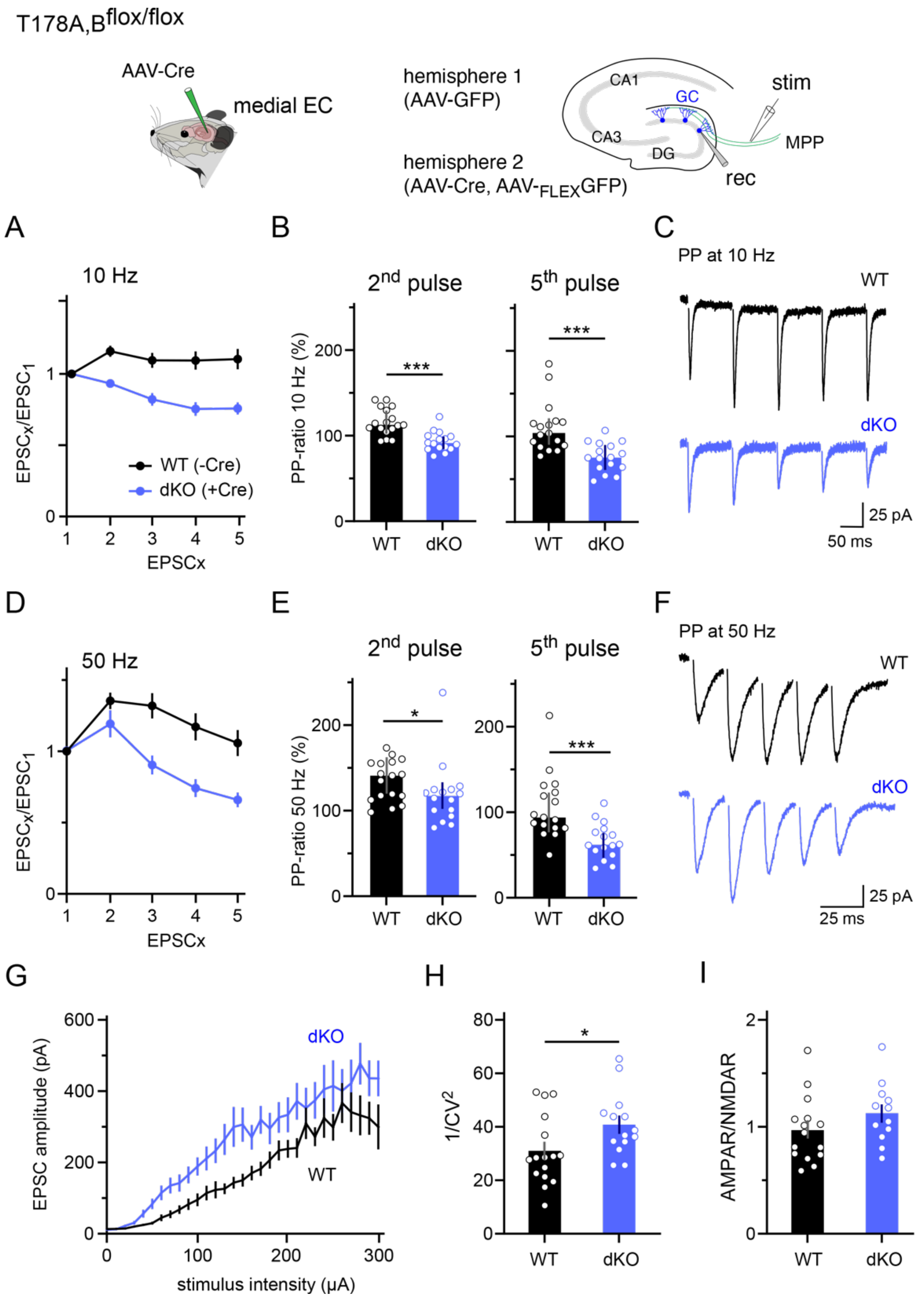

Supplementary Figure 11

Alterations in pre-synaptic function induced by knockout of T178A,B

Induction of T178A,B double knock-out in the medial entorhinal cortex (EC) of T178A,B flox/flox mice by AAV-driven expression of the Cre-recombinase. Patch-

clamp recording of AMPAR- and NMDAR-mediated EPSCs in DG granule cells (GCs) upon stimulation of the medial perforant path fibres (MPP).

(A), EPSC amplitudes measured in train stimulations at a frequency of 10 Hz and normalized to the peak-amplitude of the first EPSC. Data are mean ± SEM of 17 (WT) and 16 (dKO) GCs, respectively.

(B), Summary data of $EPSC_2/EPSC_1$ (left, 2nd pulse) and $EPSC_5/EPSC_1$ (right, 5th pulse) ratios (median ± quartiles; p-values <0.001 between WT and dKO, two-tailed Mann-Whitney U-test).

(C), Representative AMPAR-mediated EPSC trains measured at 10 Hz stimulation.

(D – F), Experiments as in (A – C) but at a stimulation frequency of 50 Hz. EPSC ratios are different between WT and dKO at p-values <0.05 (2nd pulse) and <0.001 (5th pulse, two-tailed Mann-Whitney U-test).

(G), Input-output relation measured with increasing stimulation intensity (0-300 µA).

(H), Variance ($1/CV^2$, mean ± SEM) calculated over 50 EPSCs recorded in 16 (WT) 14 (dKO) GCs. Values were different between WT and dKO at $p<0.05$ (unpaired t-test).

(I) Ratios of AMPAR- and NMDAR-mediated currents are not significantly different between WT and dKO GCs. Data are mean (± SEM) of 15 (WT) and 12 (dKO) GCs. (p-value 0.18, unpaired t-test).

Supplementary Figure 12 (information related to Figure 5)

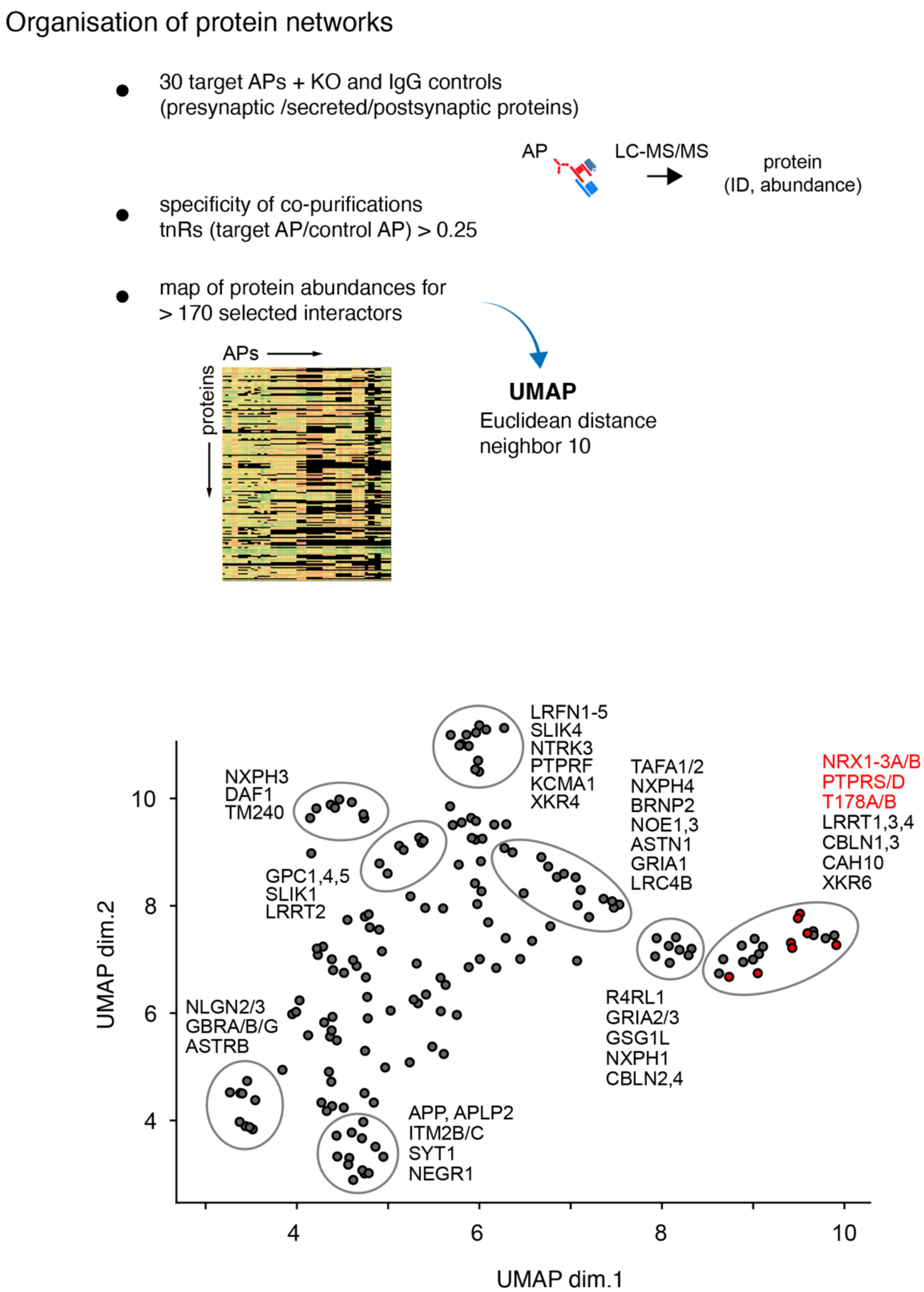

Supplementary Figure 12

UMAP (Uniform Manifold Approximation and Projection, [77]) cluster-analysis performed with the MS-derived abundance values of all proteins (total of 172) that were specifically co-purified in NRX1-3 and reverse APs with a total of 30 different target proteins. UMAP-derived subclusters (of similar behavior over the entire set of APs) were encircled with selected constituents annotated.

Supplemental Methods

Electrophysiology

In-vivo stereotactic Injection

Surgeries were performed on 3-week-old T178A,B flox/flox mice of either sex. Animals were injected with a single dose of caprofen (5 mg/kg i.p) 30 min before surgery. Animals were fixed in a stereotaxic frame and kept under isoflurane anesthesia (4% induction, 1-2% surgery). 1% lidocaine and 0.25 % bupivacaine were applied topically to the scalp before incision. Thin glass pipettes pulled from glass capillaries (WPI, #504949), were filled with a mix of AAV9 hSyn FLEx eGFP (5.7 x 10^{12} gcp/ml) and AAV2 hSyn iCre (5.4 x 10^{11} gcp/ml) or AAV9 hSyn eGFP (1.1 x 10^{13} gcp/ml) alone. Viruses were injected into the medial entorhinal cortex (EC) area at coordinates in relation to Bregma (AP, -4 mm; ML, -3.2 mm; DV, -2.95, -3.05, -3.15 mm). For each animal one hemisphere was injected with a combination of FLEx eGFP and Cre (knock-out), the other hemisphere was injected with eGFP alone (control). For each injection site a total of 180 nl (60 nl per DV coordinate) of the virus mix was injected at 60 nl/min with Nanoliter2020 Injector (WPI). The pipette was left in place for 5 min to allow diffusion of the viruses. Mice were left to recover on a heating pad for 2-3 h and then returned to home cages.

Slice preparation and patch-clamp recordings in brain slices

2-3 weeks after the stereotaxic injections mice were anesthetized with a mixture of Ketamine/Xylazine (120 mg and 16 mg/kg respectively) and cardiac-perfused with an ice-cold sucrose-based solution composed of (in mM): 2 KCl, 26 $NaHCO_3$, 1.15 NaH_2PO_4, 10 D-glucose, 220 sucrose, 0.2 $CaCl_2$, 6 $MgCl_2$ (320 mOsm/L). Brains were rapidly removed, dissected into hemispheres, placed in the cutting chamber with ice-cold sucrose-based solution, and hippocampal-entorhinal cortex brain slices were cut to 300 µm with a Leica VT 1200S vibratome. Slices were transferred to the incubation chamber containing aCSF composed of (in mM): 126 NaCl, 2.5 KCl, 1.25 NaH_2PO_4, 25 $NaHCO_3$, 12.1 D-glucose, 1.3 $MgCl_2$, 2.5 $CaCl_2$ (300 mOsm/L) placed in a water bath heated to 32ºC. After 15 min, slices were moved to room temperature and left to recover for 1h. All solutions were continuously equilibrated with carbogen.

Slice-recordings were performed in a chamber continuously perfused with carbogen-equilibrated aCSF (kept at a temperature of 30-32°C by an in-line solution heater (WPI)) composed of (in mM): 126 NaCl, 2.5 KCl, 1.25 NaH_2PO_4, 25 $NaHCO_3$, 12.1 D-glucose, 1.3 $MgCl_2$, 2.5 $CaCl_2$, 100 Picrotoxin (HelloBio; #HB0506). A monopolar glass stimulation electrode filled with aCSF and connected to a constant voltage stimulus isolator (IsoFlex, A.M.P.I.) was placed in the medial perforant path (MPP) of the hippocampal dentate gyrus defined by the eGFP signal. Electrode placement and patching was visually guided by observing the preparation with oblique illumination of infrared light through a 16× water immersion objective (Nikon, CF175 LWD) and 1-2× optical zoom and a ROLERA™ Bolt CMOS camera controlled by QCapture 2.9.13 software (QImaging). Patch pipettes (4-6 MΩ) were

pulled from borosilicate glass (Science Products GmbH, #BF150-86-10) and filled with a cesium-based internal solution composed of (in mM): 135 cesium methanesulfonate (Sigma-Aldrich, #C1426), 10 HEPES (Sigma-Aldrich, #H4034), 4 NaCl, 0.6 EGTA (Sigma-Aldrich, #3889), 0.1 $CaCl_2$, 2 $MgCl_2$, 2 Na_2-ATP (Sigma-Aldrich, #A7699), 0.3 Na-GTP (Sigma-Aldrich, #51120) (pH 7.3, 290 mOsm/L).

For patch-clamp recordings of AMPAR-mediated EPSCs in whole-cell configuration, granule cells (GCs) in the dentate gyrus were kept a holding potential of -70 mV. Analogue voltage signals were filtered online and digitized at 10 kHz directly to the computer hard disk using an MultiClamp 700B and Digidata 1440A controlled by MultiClamp and Clampex 10.7.0.3 softwares (Molecular Devices) from a computer workstation running Windows 10 (Microsoft). For recordings of short-term synaptic plasticity, trains of five electrical stimulations (0.3 ms) at different frequencies (10 Hz, 20 Hz, 50 Hz) were delivered. Each protocol consisted of 10 sweeps of trains delivered at 0.1 Hz. For determination of $EPSC_x/EPSC_1$ amplitude ratios consecutive EPSCs were measured using Clampfit 11.4.2 software (Molecular Devices) and averaged. For the input-output relation, stimulation intensity was increased by 10 µA increments from 0 to 300 µA. For the mean variance analysis 50 EPSC were recorded at 0.1 Hz and $1/CV^2$ was calculated ($1/CV^2$ = (mean amplitude)^2/ (SD)^2). AMPAR/NMDAR current ratios were obtained from five EPSCs recorded at potentials of -70 and +40 mV, respectively, and averaged. NMDAR-mediated EPSC amplitude was measured 5 ms after the onset of the EPSC.

All data was analyzed in Clampfit 11.4.2 software (Molecular Devices). All statistical analysis was done with GraphPad Prism 10.2.0 software. To assess normal distribution a Shapiro-Wilk normality test was used; depending on the result data were analyzed with parametric or non-parametric tests.

Supplementary Tables

Supplementary Table 1

Characterization of anti-NRX ABs by MS-analysis in eluates of target APs (related to Figs. 1C-E)

**Anti-NRX1-3 ABs (pan-Neurexin)**	***targeted epitope***	**PSM / Sequences**	**Sequence coverage**	**Protein abundance** (AbundanceNorm)
**#1**	mouse NRX1a, C-terminus	NRX1: 670 / 73<br>NRX2: 194 / 75<br>NRX3: 181 / 60	86.2%<br>81.6%<br>69.8%	18,568,884<br>4,365,946<br>5,443,053
**#2**	mouse NRX3a: aa1523-1571	NRX1: 153 / 67<br>NRX2: 110 / 64<br>NRX3: 137 / 69	67.2%<br>60.7%<br>66.4%	2,739,945<br>1,208,948<br>3,702,084
**#3**	mouse NRX3a: aa1471-1491<br>rat NRX1a: aa1328 -1421	NRX1: 150 / 61<br>NRX2: 28 / 18<br>NRX3: 81 / 44	66.3%<br>13.2%<br>44.1%	6,051,837<br>1,155,983<br>1,441,228
**#4**	rat NRX1/2/3, C-terminus	NRX1: 311 / 88<br>NRX2: 152 / 79<br>NRX3: 149 / 71	79.2%<br>68.8%<br>60.4%	8,884,697<br>195,795<br>270,329

**Anti-NRX1**	***targeted epitope***	**PSM / Sequences**	**Sequence coverage**	**Protein abundance** (AbundanceNorm)
**#1**	rat NRX1a: aa1328 -1421	NRX1: 344 / 79	81.3%	5,335,601
**#2**	rat NRX1b: aa 47-392	NRX1: 153 / 67	76.5%	6,595,500
**#3**	anti-HA<br>(for Nrx1a-HA Knockin, Sterky F. *et al.*)	NRX1: 422 / 74	66.2%	5,263,383

**Anti-NRX2**	***targeted epitope***	**PSM / Sequences**	**Sequence coverage**	**Protein abundance** (AbundanceNorm)
**#1**	mouse NRX2a, aa 266-282	NRX2: 56 / 44	39%	891,147
**#2**	mouse NRX2a: aa1459-1476	NRX2: 71 / 50	43.3%	260,388
**#3**	mouse NRX2a: aa1459-1476	NRX2: 65 / 47	39.2%	169,506
**#4**	(undisclosed)	NRX2: 76 / 56	46.5%	703,839

**Anti-NRX3**	***targeted epitope***	**PSM / Sequences**	**Sequence coverage**	**Protein abundance** (AbundanceNorm)
**#1**	mouse NRX3, aa1349-1368	NRX3: 221 / 64	58.2%	12,462,808
**#2**	rat NRX3, aa1298-1494	NRX3: 105 / 60	59.9%	1,799,555
**#3**	mouse NRX3, aa1377-1397	NRX3: 174 / 79	64.3%	1,822,274

**#4**	human NRX3 (NP_620426), aa35-357	NRX3: 666 / 95	75.9%	6,894,268
**#5**	mouse NRX3, aa1377-1397	NRX3: 164 / 63	59.7%	3,796,077

PSM: peptide-spectrum matches (MS/MS spectra assigned to a given protein)
Sequences: number of distinct peptide sequences identified by MS/MS spectra
AbundanceNorm: Sum of protein-specific peptide intensities (PVs) / number of MS-accessible amino acids (arbitrary units)

Supplementary Table 2

tnR-values of APs with the anti-NRX1-3 ABs (**Table 1**), and with the sub-type specific anti-NRX1, 2, 3 ABs

Table S2 tnR-values of APs wit anti-Nrx1-3

NRX1-3	anti-Nrx1-3 exp.1:AB#1	anti-Nrx1-3 exp.2:AB#1	anti-Nrx1-3 exp.3:AB#1	anti-Nrx1-3 exp.4:AB#1	anti-Nrx1-3 exp.1:AB#2	anti-Nrx1-3 exp.2:AB#2	anti-Nrx1-3 exp.3:AB#2	anti-Nrx1-3 exp.4:AB#2	anti-Nrx1-3 exp.1:AB#3	anti-Nrx1-3 exp.2:AB#3	anti-Nrx1-3 exp.1:AB#4	anti-Nrx1-3 exp.2:AB#4	anti-Nrx1-3 exp.3:AB#4	anti-Nrx1-3 exp.4:AB#4
NRX1A	1.00	1.00	1.00	1.00	1.00	1.00	1.00	1.00	1.00	1.00	1.00	1.00	1.00	1.00
NRX1B	0.90	0.90	0.69	0.83	0.00	0.00	0.89	0.88	0.00	0.00	0.86	0.86	0.64	0.75
NRX2A	0.98	1.00	1.00	0.98	0.99	1.00	1.00	1.00	0.60	0.55	0.91	0.93	0.86	0.86
NRX2B	0.99	1.00	0.90	0.92	0.91	0.91	1.00	1.00	0.63	0.58	0.94	0.96	0.86	0.90
NRX3A	0.80	1.00	1.00	1.00	0.94	1.00	1.00	1.00	0.78	1.00	0.73	0.99	0.95	0.89
NRX3B	0.73	0.70	0.62	0.69	0.88	0.88	0.99	1.00	0.71	0.68	0.66	0.61	0.62	0.75
A4	0.55	0.66	0.41	0.60	0.17	0.25	0.00	0.00	0.30	0.38	0.41	0.53	0.31	0.53
AGRB1	0.49	0.41	0.10	0.40	0.40	0.23	0.00	0.00	0.00	0.00	0.45	0.34	0.00	0.31
APLP2	0.72	0.70	0.38	0.62	0.45	0.31	0.56	0.34	0.00	0.00	0.55	0.48	0.25	0.52
ASTN1	0.46	0.65	0.42	0.63	0.42	0.75	0.79	0.83	0.00	0.00	0.29	0.49	0.17	0.41
ASTRB	0.32	0.19	0.00	0.08	0.40	0.20	0.75	0.21	0.52	0.42	0.11	0.00	0.00	0.27
BRNP1	0.55	0.50	0.17	0.52	0.53	0.42	0.69	0.60	0.00	0.00	0.46	0.37	0.00	0.00
BRNP2	0.52	0.45	0.05	0.46	0.47	0.34	0.65	0.52	0.00	0.00	0.00	0.00	0.00	0.00
C1QL3	0.35	0.39	0.13	0.34	0.03	0.00	0.24	0.18	0.06	0.04	0.25	0.27	0.51	0.44
CADM3	0.72	0.65	0.44	0.42	0.38	0.13	0.78	0.27	0.00	0.00	0.00	0.00	0.00	0.15
CAH10	1.00	1.00	0.89	0.99	1.00	1.00	0.97	1.00	0.84	0.82	1.00	1.00	0.88	0.83
CAH11	0.83	0.84	0.87	0.85	0.88	0.91	0.93	1.00	0.73	0.72	0.87	0.89	0.94	0.88
CALR	0.58	0.52	0.25	0.39	0.39	0.23	0.62	0.44	0.83	0.82	0.53	0.45	0.17	0.47
CBLN1	0.73	0.87	0.90	0.91	0.70	0.94	1.00	1.00	0.52	0.63	0.70	0.87	0.86	0.97
CBLN2	0.78	0.78	0.48	0.80	0.68	0.64	0.78	0.79	0.64	0.60	0.78	0.78	0.54	0.63
CBLN3	0.92	0.84	0.85	0.80	1.00	0.88	1.00	1.00	0.72	0.57	0.93	0.83	0.80	0.95
CBLN4	0.76	0.74	0.59	0.75	0.58	0.49	0.82	0.75	0.57	0.51	0.72	0.68	0.52	0.69
CLUS	0.36	0.38	0.07	0.35	0.02	0.00	0.72	0.18	0.16	0.13	0.24	0.22	0.00	0.60
CNTN1	0.32	0.18	0.00	0.13	0.04	0.00	0.51	0.00	0.46	0.33	0.18	0.00	0.00	0.30
CXB1	0.65	0.61	0.40	0.41	0.40	0.23	0.82	0.80	0.32	0.19	0.44	0.33	0.00	0.32
GRIA1	0.34	0.27	0.00	0.21	0.01	0.00	0.64	0.00	0.15	0.02	0.21	0.09	0.00	0.61
GRIA2	0.39	0.29	0.00	0.28	0.04	0.00	0.45	0.00	0.22	0.07	0.22	0.06	0.00	0.49
GRIA3	0.42	0.35	0.00	0.37	0.09	0.00	0.60	0.00	0.21	0.08	0.26	0.13	0.00	0.61
GRM4	0.35	0.18	0.00	0.22	0.05	0.00	0.60	0.00	0.31	0.10	0.22	0.00	0.00	0.49
GSLG1	0.57	0.52	0.40	0.50	0.37	0.21	0.68	0.53	0.00	0.00	0.43	0.33	0.24	0.52
ITM2B	0.56	0.60	0.23	0.55	0.16	0.08	0.68	0.17	0.13	0.07	0.42	0.43	0.07	0.65
ITM2C	0.53	0.53	0.14	0.49	0.09	0.00	0.72	0.13	0.11	0.01	0.47	0.46	0.20	0.80
KCJ10	0.50	0.44	0.00	0.00	0.32	0.16	0.84	0.62	0.45	0.37	0.53	0.47	0.00	0.29
KCMA1	0.42	0.31	0.00	0.31	0.55	0.38	0.90	0.42	0.38	0.23	0.30	0.12	0.00	0.43
LRC4B	0.59	0.53	0.26	0.43	0.44	0.29	0.62	0.43	0.00	0.00	0.45	0.34	0.00	0.33
LRFN1	0.60	0.55	0.28	0.49	0.44	0.29	0.66	0.43	0.00	0.00	0.49	0.40	0.01	0.43
LRFN3	0.65	0.60	0.30	0.45	0.47	0.33	0.65	0.49	0.00	0.00	0.49	0.40	0.10	0.43
LRRT1	1.00	0.90	0.75	0.96	0.74	0.29	0.90	0.58	0.62	0.30	0.73	0.42	0.28	0.75
LRRT2	0.53	0.49	0.45	0.48	0.32	0.18	0.56	0.51	0.00	0.00	0.41	0.33	0.30	0.44
LRRT3	0.73	0.66	0.52	0.77	0.21	0.00	0.62	0.15	0.33	0.15	0.42	0.25	0.00	0.56
LRRT4	0.99	0.86	0.64	0.89	0.49	0.07	0.64	0.10	0.43	0.15	0.65	0.40	0.00	0.60
GP158/MGLYR	0.44	0.42	0.00	0.11	0.04	0.00	0.55	0.19	0.42	0.40	0.34	0.30	0.00	0.29
NLGN1	0.35	0.26	0.33	0.25	0.00	0.00	0.39	0.10	0.00	0.00	0.00	0.00	0.09	0.41
NLGN2	0.29	0.11	0.00	0.00	0.04	0.00	0.57	0.00	0.00	0.00	0.20	0.00	0.00	0.49
NLGN3	0.24	0.46	0.35	0.37	0.00	0.00	0.62	0.45	0.00	0.00	0.00	0.00	0.20	0.48
NSG2	0.52	0.45	0.21	0.38	0.00	0.00	0.47	0.17	0.00	0.00	0.00	0.00	0.00	0.24
NXPH1	0.79	0.78	0.70	0.72	0.77	0.74	0.86	0.83	0.58	0.51	0.77	0.75	0.64	0.75
NXPH3	0.75	0.73	0.64	0.73	0.56	0.47	0.75	0.72	0.55	0.49	0.66	0.62	0.49	0.61
NXPH4	0.54	0.47	0.25	0.51	0.19	0.00	0.46	0.16	0.28	0.14	0.44	0.33	0.01	0.38
PCDH1	0.28	0.47	0.19	0.44	0.00	0.23	0.66	0.48	0.00	0.00	0.42	0.69	0.45	0.66
PRRT1	0.43	0.35	0.07	0.29	0.45	0.32	0.67	0.48	0.25	0.12	0.70	0.67	0.53	0.71
PTPRD	0.90	0.90	0.75	0.86	0.93	0.93	1.00	0.89	0.47	0.37	0.75	0.71	0.50	0.80
PTPRF	0.73	0.71	0.50	0.66	0.68	0.63	0.72	0.66	0.00	0.00	0.58	0.52	0.22	0.47
PTPRS	0.85	0.81	0.68	0.79	0.85	0.77	1.00	0.78	0.47	0.34	0.70	0.62	0.41	0.82
R4RL1	0.82	0.81	0.59	0.74	0.76	0.72	0.82	0.75	0.00	0.00	0.75	0.72	0.51	0.69
RGS7	0.38	0.34	0.00	0.05	0.04	0.00	0.54	0.12	0.36	0.30	0.29	0.21	0.00	0.21
RTN4R	0.68	0.64	0.42	0.56	0.55	0.42	0.71	0.54	0.49	0.40	0.61	0.54	0.22	0.54
SLIT1	0.51	0.44	0.26	0.40	0.00	0.00	0.64	0.46	0.00	0.00	0.00	0.00	0.00	0.37
SLIT2	0.46	0.38	0.24	0.42	0.00	0.00	0.48	0.25	0.00	0.00	0.00	0.00	0.00	0.30
SLIT3	0.29	0.45	0.22	0.53	0.00	0.00	0.60	0.36	0.03	0.00	0.13	0.29	0.00	0.00
SO3A1	0.79	0.81	0.72	0.75	0.75	0.77	0.75	0.87	0.00	0.00	0.79	0.81	0.72	0.66
SYT11	0.51	0.47	0.00	0.36	0.13	0.00	0.67	0.05	0.29	0.19	0.28	0.17	0.00	0.44
T132A	0.60	0.55	0.13	0.49	0.00	0.00	0.31	0.20	0.00	0.00	0.44	0.34	0.00	0.19
T178A	0.78	0.76	0.65	0.77	0.75	0.71	0.72	0.67	0.26	0.12	0.64	0.59	0.29	0.50
T178B	0.88	1.00	1.00	1.00	0.94	1.00	1.00	1.00	0.32	0.32	0.79	0.90	0.76	0.85
TAFA1	0.66	0.62	0.34	0.40	0.46	0.32	0.76	0.60	0.41	0.31	0.54	0.45	0.14	0.51
TAFA2	0.69	0.66	0.44	0.54	0.57	0.48	0.79	0.72	0.42	0.33	0.59	0.53	0.25	0.52
TRPV2	0.43	0.49	0.11	0.51	0.05	0.03	0.71	0.33	0.00	0.00	0.19	0.21	0.00	0.44
VWA8	0.27	0.10	0.00	0.06	0.00	0.00	0.48	0.00	0.50	0.34	0.16	0.00	0.00	0.26
XKR4	0.55	0.49	0.00	0.42	0.37	0.19	0.00	0.00	0.48	0.40	0.44	0.34	0.00	0.00
XKR6	0.80	0.80	0.76	0.82	0.76	0.74	0.81	0.85	0.82	0.82	0.56	0.51	0.43	0.57

Supplementary Table 3

tnR-values of APs with anti-NRX1-3 and anti-PTPRs ABs from ER-membranes

Table S3 tnR-values of APs wit anti-Nrx1-3 and anti-PTPRS from ER-membranes

NRX1-3	anti-Nrx1-3<br>exp.1:AB#1-vs-IgG#1
NRX1A	1.00
NRX1B	0.76
NRX2A	0.81
NRX2B	0.85
NRX3A	0.81
PTPRD	0.45
PTPRS	0.58
T178A	0.19
T178B	0.66
CBLN1	0.80
CBLN2	0.64
CBLN3	0.87
CBLN4	0.69
CAH10	0.63
C1QL3	0.38
NXPH1	0.55
ITM2B	0.59
ITM2C	0.80
R4RL1	0.55
SYT11	0.60
T132A	0.45
LIPA2	0.42
LIPA3	0.46
GRM4	0.61
CAC1B	0.34

PTPRS	anti-PTPRS<br>exp.1:AB#1-vs-IgG#1
PTPRS	1.00
PTPRD	0.24
PTPRF	0.56
T178A	0.30
T178B	0.93
NRX1A	0.62
NRX2A	0.45
NRX2B	0.48
NRX3A	0.48
SYT11	0.59
T132A	0.41
LIPA2	0.40
LIPA3	0.50
LIPA4	0.36
LRFN1	0.58
LRFN3	0.51
LRFN4	0.57
LRFN5	0.55
GRM4	0.41
CAC1B	0.23

Supplementary Table 4

tnR-values of specifically co-purified proteins in the target APs indicated in Figure 5

Table S4 tnR-values of APs related to Figures 5, 6

Antibodies	AB#1, anti-HA, Roche, #11867423001
	AB#2, anti-HA, Thermo Fisher, #26183
	IgG, Millipore (#12-370)
	IgG, RnD Systems
source	Knock-In Cbln1-HA
	Knock-In Cbln2-HA
	WT

CBLN1/2	CBLN1_AB#1_exp1-vs-HA(WT)	CBLN1_AB#1_exp1-vs-IgG#1	CBLN1_AB#1_exp1-vs-IgG#2	CBLN1_AB#1_exp1-vs-IgG#3	CBLN1_AB#2_exp1-vs-HA(WT)	CBLN1_AB#2_exp1-vs-IgG#1
	tnR	tnR	tnR	tnR	tnR	tnR
CAH10	0.377	0.345	0.299	0.373	0.366	0.356
CBLN1	1	1	1	1	1	1
CBLN2	0.548	0.519	0.502	0.547	0.564	0.544
CBLN3	0.958	0.944	0.947	0.951	0.938	0.923
CBLN4	0.636	0.630	0.615	0.643	0.668	0.661
CSKP	0.311	0.292	0.264	0.338	0.327	0.305
GRID2	0.414	0.071	0.000	0.411	0.426	0.113
GRM4	0.538	0.519	0.497	0.529	0.538	0.532
NRX1A	0.736	0.635	0.590	0.739	0.722	0.630
NRX2A	0.529	0.512	0.487	0.529	0.534	0.528
NRX2B	0.608	0.609	0.589	0.627	0.586	0.586
NRX3A	0.243	0.252	0.201	0.262	0.275	0.293
NXPH1	0.381	0.361	0.332	0.372	0.000	0.000
PTPRD	0.294	0.262	0.234	0.236	0.297	0.279
PTPRS	0.341	0.223	0.278	0.338	0.356	0.261
RO52	0.434	0.149	0.172	0.423	0.030	0.187
T178B	0.304	0.284	0.258	0.309	0.000	0.000
XKR6	0.392	0.365	0.336	0.407	0.408	0.390

CBLN1/2	CBLN1_AB#2_exp2-vs-IgG#2	CBLN1_AB#2_exp1-vs-IgG#3	CBLN1_AB#1_exp2-vs-HA(WT)	CBLN1_AB#1_exp1-vs-IgG#4	CBLN1_AB#2_exp2-vs-IgG#5	CBLN1_AB#2_exp2-vs-HA(WT)
	tnR	tnR	tnR	tnR	tnR	tnR
CAH10	0.313	0.383	0.464	0.261	0.440	0.501
CBLN1	1	1	1	1	1	1
CBLN2	0.529	0.571	0.615	0.466	0.640	0.607
CBLN3	0.925	0.930	0.958	0.924	1.00	0.967
CBLN4	0.648	0.673	0.661	0.561	0.751	0.674
CSKP	0.278	0.348	0.177	0.000	0.197	0.277
GRID2	0.000	0.437	0.437	0.000	0.138	0.514
GRM4	0.512	0.542	0.359	0.000	0.356	0.439
NRX1A	0.586	0.729	0.719	0.402	0.725	0.772
NRX2A	0.505	0.544	0.364	0.445	0.610	0.616
NRX2B	0.566	0.603	0.582	0.462	0.670	0.636
NRX3A	0.247	0.302	0.410	0.229	0.465	0.459
NXPH1	0.000	0.000	0.459	0.275	0.491	0.512
PTPRD	0.253	0.254	0.405	0.000	0.286	0.497
PTPRS	0.316	0.371	0.429	0.000	0.210	0.504
RO52	0.211	0.448	0.065	0.230	0.428	0.153
T178B	0.000	0.000	0.444	0.279	0.491	0.492
XKR6	0.363	0.430	0.316	0.087	0.306	0.355

CBLN1/2	CBLN1_AB#2_exp2-vs-IgG#4	CBLN1_AB#2_exp2-vs-IgG#5	CBLN2_AB#1_exp1-vs-IgG#1	CBLN2_AB#1_exp1-vs-IgG#2	CBLN2_AB#1_exp1-vs-IgG#3	CBLN2_AB#1_exp1-vs-HA(WT)
	tnR	tnR	tnR	tnR	tnR	tnR
CAH10	0.286	0.466	0.210	0.386	0.479	0.000
CBLN1	1	1	0.785	0.710	0.837	0.829
CBLN2	0.441	0.623	1	1	1	1
CBLN3	0.943	1.00	0.000	0.000	0.000	0.000
CBLN4	0.561	0.756	0.812	0.887	0.849	0.802
CSKP	0.029	0.279	0.000	0.271	0.297	0.000
GRID2	0.000	0.211	0.000	0.000	0.000	0.000
GRM4	0.000	0.423	0.000	0.000	0.000	0.000
NRX1A	0.456	0.781	0.485	0.768	0.833	0.798
NRX2A	0.469	0.635	0.418	0.549	0.597	0.467
NRX2B	0.510	0.719	0.348	0.546	0.538	0.000
NRX3A	0.251	0.492	0.397	0.587	0.585	0.370
NXPH1	0.325	0.542	0.445	0.606	0.616	0.488
PTPRD	0.000	0.370	0.000	0.337	0.523	0.353
PTPRS	0.000	0.275	0.000	0.294	0.582	0.449
RO52	0.180	0.390	0.331	0.495	0.247	0.660
T178B	0.324	0.537	0.383	0.556	0.553	0.331
XKR6	0.114	0.337	0.037	0.280	0.340	0.000

CBLN1/2	CBLN2_AB#2_exp1-vs-IgG#1	CBLN2_AB#2_exp1-vs-IgG#2	CBLN2_AB#2_exp1-vs-IgG#3	CBLN2_AB#2_exp1-vs-HA(WT	CBLN2_AB#1_exp2-vs-IgG#4	CBLN2_AB#1_exp2-vs-IgG#5
	tnR	tnR	tnR	tnR	tnR	tnR
CAH10	0.000	0.000	0.000	0.128	0.333	0.411
CBLN1	0.853	0.829	0.834	0.725	0.663	0.780
CBLN2	1	1	1	1	1	1
CBLN3	0.000	0.000	0.000	0.000	0.000	0.000
CBLN4	0.821	0.805	0.812	0.724	0.825	0.778
CSKP	0.000	0.000	0.000	0.000	0.181	0.175
GRID2	0.000	0.000	0.000	0.000	0.000	0.000
GRM4	0.000	0.000	0.000	0.000	0.000	0.000
NRX1A	0.700	0.639	0.805	0.454	0.759	0.816
NRX2A	0.460	0.418	0.469	0.406	0.546	0.304
NRX2B	0.000	0.000	0.000	0.246	0.480	0.448
NRX3A	0.394	0.336	0.394	0.365	0.572	0.539
NXPH1	0.479	0.445	0.478	0.355	0.547	0.548
PTPRD	0.326	0.291	0.285	0.000	0.253	0.427
PTPRS	0.319	0.388	0.447	0.000	0.211	0.500
RO52	0.333	0.370	0.648	0.414	0.564	0.146
T178B	0.318	0.283	0.339	0.309	0.509	0.494
XKR6	0.000	0.000	0.000	0.000	0.262	0.305

CBLN1/2	CBLN2_AB#1_exp2-vs-HA(WT)	CBLN2_AB#2_exp2-vs-IgG#4	CBLN2_AB#2_exp2-vs-IgG#5	CBLN2_AB#2_exp2-vs-HA(WT)
	tnR	tnR	tnR	tnR
CAH10	0.335	0.335	0.279	0.358
CBLN1	0.801	0.830	0.807	0.813
CBLN2	1	1	1	1
CBLN3	0.000	0.000	0.000	0.000
CBLN4	0.840	0.855	0.842	0.845
CSKP	0.260	0.244	0.206	0.288
GRID2	0.000	0.000	0.000	0.000
GRM4	0.000	0.000	0.000	0.000
NRX1A	0.771	0.686	0.630	0.782
NRX2A	0.480	0.489	0.453	0.496
NRX2B	0.463	0.479	0.442	0.489
NRX3A	0.382	0.413	0.362	0.412
NXPH1	0.502	0.509	0.480	0.507
PTPRD	0.300	0.289	0.255	0.253
PTPRS	0.425	0.327	0.390	0.443
RO52	0.187	0.373	0.409	0.657
T178B	0.406	0.403	0.376	0.418
XKR6	0.000	0.000	0.000	0.000

Table S4 tnR-values of APs related to Figures 5, 6

Antibodies	AB#1, anti-CAH10, (raised against recombinant CAH10 (PMID: 33586859), rabbit, #Hadlai)
	AB#2, anti-CAH10, (raised against recombinant CAH10 (PMID: 33586859), rabbit, #Habeba)
	AB#3, anti-CAH10, RnD, #MAB2189
	IgG, Millipore (#12-370)
	IgG (Bioss, #11104R)
source	WT

CAH10	AB#1-vs-IgG#1	AB#1-vs-IgG#2	AB#1-vs-IgG#3	AB#2-vs-IgG#1	AB#2-vs-IgG#2	AB#2-vs-IgG#3	AB#3-exp1-vs-IgG#1	AB#3-exp2-vs-IgG#1	AB#3-exp2-vs-IgG#2	AB#3-exp2-vs-IgG#3
	tnR	tnR	tnR	tnR	tnR	tnR	tnR	tnR	tnR	tnR
CADM3	0.318	0.281	0.412	0.290	0.253	0.378	0.000	0.187	0.137	0.273
CAH10	1	1	1	1	1	1	1	1	1	1
CAH11	0.849	0.851	1000	0.849	0.850	1000	0.794	0.613	0.616	0.901
CBLN1	0.570	0.469	0.936	0.565	0.462	0.937	0.619	0.278	0.138	0.719
CBLN2	0.542	0.545	0.717	0.560	0.564	0.745	0.362	0.221	0.229	0.344
CBLN3	0.610	0.608	0.806	0.620	0.618	0.823	0.514	0.237	0.237	0.366
CBLN4	0.563	0.561	0.720	0.567	0.566	0.729	0.193	0.257	0.257	0.358
CD47	0.363	0.370	0.487	0.321	0.328	0.434	0.000	0.000	0.000	0.000
CSKP	0.153	0.002	0.212	0.427	0.272	0.573	0.295	0.464	0.250	0.711
G37L1	0.149	0.458	0.577	0.104	0.418	0.526	0.000	0.000	0.381	0.533
GLRA1	0.197	0.214	0.432	0.225	0.243	0.475	0.000	0.000	0.000	0.198
GLRA2	0.188	0.171	0.422	0.239	0.222	0.495	0.000	0.000	0.000	0.186
GLRB	0.061	0.102	0.548	0.217	0.258	0.765	0.000	0.000	0.000	0.663
GPC1	0.145	0.144	0.187	0.354	0.351	0.463	0.000	0.000	0.000	0.000
ITM2B	0.236	0.222	0.500	0.228	0.213	0.495	0.000	0.000	0.000	0.236
KCJ10	0.401	0.440	0.536	0.410	0.450	0.551	0.000	0.047	0.105	0.088
LIN7A	0.175	0.177	0.223	0.244	0.246	0.314	0.334	0.000	0.000	0.000
LIN7B	0.309	0.313	0.397	0.311	0.315	0.401	0.505	0.264	0.269	0.382
LIPA4	0.050	0.092	0.041	0.194	0.236	0.231	0.252	0.169	0.227	0.215
LRFN1	0.212	0.213	0.278	0.368	0.368	0.484	0.209	0.000	0.000	0.000
LRFN3	0.230	0.234	0.315	0.323	0.327	0.439	0.000	0.000	0.000	0.000
LRRT1	0.174	0.175	0.225	0.442	0.441	0.579	0.326	0.123	0.125	0.180
LRRT3	0.131	0.062	0.185	0.383	0.311	0.518	0.187	0.000	0.000	0.000
LRRT4	0.188	0.190	0.237	0.244	0.246	0.312	0.000	0.000	0.000	0.000
LSAMP	0.026	0.216	0.267	0.105	0.297	0.376	0.292	0.000	0.245	0.342
NEGR1	0.343	0.120	0.461	0.349	0.122	0.471	0.000	0.239	0.000	0.375
NOE1	0.096	0.096	0.037	0.316	0.315	0.325	0.456	0.294	0.292	0.297
NOE3	0.203	0.202	0.264	0.284	0.283	0.373	0.371	0.308	0.306	0.460
NRX1A	0.448	0.547	0.505	0.696	0.795	0.833	0.649	0.515	0.653	0.639
NRX2A	0.400	0.400	0.493	0.687	0.686	0.874	0.627	0.636	0.634	0.903
NRX2B	0.427	0.426	0.549	0.691	0.689	0.900	0.449	0.477	0.475	0.698
NRX3A	0.254	0.265	0.350	0.459	0.469	0.622	0.078	0.246	0.261	0.394
NXPH1	0.370	0.369	0.486	0.408	0.407	0.538	0.308	0.000	0.000	0.000
NXPH4	0.255	0.253	0.336	0.284	0.282	0.376	0.117	0.000	0.000	0.000
PLPP3	0.315	0.336	0.452	0.289	0.310	0.421	0.000	0.000	0.000	0.000
PTPRD	0.376	0.422	0.405	0.515	0.561	0.589	0.356	0.306	0.372	0.320
PTPRF	0.175	0.178	0.242	0.328	0.330	0.445	0.255	0.133	0.136	0.218
PTPRS	0.300	0.307	0.331	0.462	0.467	0.544	0.190	0.244	0.253	0.265
S61A2	0.347	0.363	0.389	0.340	0.356	0.381	0.118	0.000	0.000	0.000
SC6A1	0.000	0.256	0.018	0.074	0.415	0.223	0.336	0.000	0.467	0.192
T178A	0.327	0.330	0.442	0.344	0.347	0.467	0.202	0.000	0.000	0.000
T178B	0.599	0.599	0.771	0.624	0.623	0.807	0.537	0.382	0.383	0.550
XKR4	0.173	0.175	0.223	0.277	0.278	0.360	0.000	0.000	0.000	0.000
XKR6	0.358	0.351	0.386	0.551	0.542	0.640	0.790	0.678	0.665	0.881

Table S4 tnR-values of APs related to Figures 5, 6

Antibodies AB#1, anti-PTPRS, RnD Systems, #AF3430
AB#2, anti-PTPRS, Invitrogen, #PA5-47487
AB#3, anti-PTPRS, MediMabs, #MM-0020
IgG, Millipore, #12-370
source WT

**PTPRS**	**AB#1-vs-IgG#1**	**AB#1-vs-IgG#2**	**AB#1-vs-IgG#3**	**AB#2-vs-IgG#1**	**AB#2-vs-IgG#2**	**AB#2-vs-IgG#3**	**AB#3-vs-IgG#1**	**AB#3-vs-IgG#2**	**AB#3-vs-IgG#3**
	**tnR**	**tnR**	**tnR**	**tnR**	**tnR**	**tnR**	**tnR**	**tnR**	**tnR**
ASTN1	0.387	0.372	0.321	0.198	0.384	0.335	0.000	0.000	0.030
BRNP1	0.408	0.352	0.331	0.000	0.368	0.350	0.000	0.058	0.000
CAC1B	0.395	0.377	0.423	0.430	0.493	0.555	0.267	0.213	0.246
CAH10	0.494	0.519	0.584	0.000	0.551	0.622	0.389	0.399	0.469
CAH11	0.390	0.379	0.420	0.521	0.491	0.549	0.000	0.000	0.000
CBLN1	0.498	0.287	0.499	0.000	0.318	0.538	0.000	0.000	0.086
CBLN2	0.417	0.413	0.470	0.528	0.481	0.549	0.000	0.000	0.000
CBLN4	0.411	0.373	0.423	0.433	0.427	0.487	0.304	0.226	0.270
CSKP	0.487	0.282	0.366	0.209	0.369	0.467	0.318	0.000	0.060
ITM2B	0.542	0.340	0.415	0.000	0.366	0.446	0.536	0.255	0.347
ITM2C	0.499	0.321	0.359	0.568	0.308	0.344	0.528	0.289	0.337
KCMA1	0.584	0.387	0.444	0.000	0.437	0.502	0.314	0.000	0.000
LIN7B	0.304	0.278	0.298	0.000	0.373	0.407	0.313	0.279	0.309
LRFN1	0.609	0.584	0.668	0.000	0.620	0.711	0.431	0.356	0.432
LRFN2	0.521	0.538	0.606	0.640	0.565	0.638	0.000	0.000	0.000
LRFN3	0.565	0.556	0.627	0.544	0.613	0.694	0.395	0.342	0.404
LRFN4	0.582	0.625	0.694	0.614	0.653	0.728	0.424	0.446	0.510
LRFN5	0.642	0.642	0.724	0.606	0.673	0.761	0.456	0.413	0.486
LRRT1	0.513	0.494	0.557	0.330	0.581	0.657	0.301	0.226	0.263
LRRT2	0.327	0.327	0.357	0.587	0.354	0.389	0.000	0.000	0.000
LRRT3	0.412	0.335	0.345	0.350	0.368	0.383	0.253	0.110	0.080
LRRT4	0.345	0.310	0.347	0.441	0.430	0.485	0.166	0.076	0.083
NDRG3	0.468	0.452	0.505	0.419	0.464	0.520	0.384	0.341	0.396
NRX1A	0.619	0.671	0.761	0.514	0.766	0.872	0.452	0.485	0.579
NRX1B	0.475	0.443	0.508	0.700	0.631	0.724	0.000	0.000	0.000
NRX2A	0.518	0.512	0.584	0.636	0.631	0.720	0.318	0.264	0.318
NRX2B	0.511	0.479	0.549	0.619	0.610	0.700	0.312	0.222	0.272
NRX3A	0.555	0.569	0.647	0.623	0.672	0.766	0.369	0.346	0.414
NTRK3	0.583	0.640	0.713	0.458	0.652	0.728	0.426	0.469	0.541
NXPH1	0.469	0.433	0.496	0.365	0.532	0.611	0.000	0.000	0.000
PCDH1	0.392	0.216	0.170	0.602	0.000	0.000	0.336	0.080	0.000
PTPRD	0.493	0.423	0.466	0.463	0.437	0.482	0.296	0.155	0.162
PTPRF	0.638	0.667	0.624	0.505	0.692	0.651	0.280	0.237	0.085
PTPRS	1	1	1	1	1	1	1	1	1
R4RL1	0.402	0.366	0.416	0.000	0.515	0.587	0.000	0.000	0.000
SLIK1	0.392	0.372	0.402	0.000	0.417	0.454	0.000	0.000	0.000
SLIK4	0.468	0.509	0.565	0.431	0.525	0.585	0.308	0.326	0.372
SYT11	0.505	0.325	0.445	0.231	0.314	0.435	0.494	0.243	0.401
T132A	0.360	0.485	0.546	0.000	0.546	0.617	0.142	0.264	0.308
T178A	0.400	0.408	0.455	0.417	0.668	0.754	0.000	0.000	0.000
T178B	0.834	0.885	1	0.622	0.836	0.946	0.889	0.973	1
TEN4	0.427	0.340	0.346	0.365	0.364	0.374	0.230	0.064	0.017
UNC5A	0.490	0.501	0.560	0.000	0.589	0.662	0.242	0.200	0.226
XKR4	0.561	0.541	0.622	0.000	0.555	0.641	0.403	0.338	0.416

Table S4 tnR-values of APs related to Figures 5, 6

Antibodies AB#1, anti-T178B, LifeTein, #RB4235
AB#2, anti-T178B, LifeTein, #RB4236
AB#3, anti-T178B, AmsBio, #3967
AB#4, anti-T178B, AmsBio, #3968
IgG, Millipore, #12-370

source WT

T178B	AB#1-vs-IgG#1	AB#1-vs-IgG#2	AB#2-vs-IgG#1	AB#2-vs-IgG#2	AB#3-vs-IgG#1	AB#3-vs-IgG#2	AB#4-vs-IgG#1	AB#4-vs-IgG#2	AB#1-exp2-vs-IgG#	AB#1-exp2-vs-IgG#	AB#2-exp2-vs-IgG#	AB#2-exp2-vs-IgG#2
	tnR	tnR	tnR	tnR	tnR	tnR	tnR	tnR	tnR	tnR	tnR	tnR
ASTN1	0.349	0.129	0.360	0.138	0.000	0.000	0.000	0.000	0.000	0.000	0.000	0.000
BRNP1	0.377	0.356	0.409	0.389	0.393	0.366	0.000	0.000	0.000	0.000	0.000	0.000
CAC1B	0.562	0.188	0.555	0.178	0.931	0.444	0.797	0.362	0.812	0.428	0.725	0.332
CAH10	0.528	0.494	0.521	0.487	0.000	0.000	0.000	0.000	0.431	0.391	0.454	0.415
CBLN1	0.172	0.158	0.181	0.168	0.000	0.000	0.003	0.000	0.029	0.009	0.000	0.000
CSKP	0.360	0.245	0.341	0.225	0.176	0.010	0.132	0.000	0.225	0.098	0.175	0.044
GBRA2	0.666	0.696	0.674	0.704	0.668	0.709	0.666	0.703	0.676	0.708	0.633	0.664
GBRB1	0.536	0.628	0.521	0.613	0.602	0.731	0.570	0.682	0.621	0.722	0.547	0.646
GBRG2	0.452	0.826	0.457	0.833	0.715	1	0.649	1	0.743	1	0.672	1
GRIA1	0.306	0.209	0.310	0.213	0.000	0.000	0.000	0.000	0.088	0.000	0.000	0.000
GRIA2	0.398	0.201	0.411	0.213	0.039	0.000	0.076	0.000	0.133	0.000	0.103	0.000
GRIA3	0.480	0.279	0.475	0.272	0.009	0.000	0.125	0.000	0.140	0.000	0.164	0.000
GRM4	0.341	0.208	0.331	0.196	0.464	0.290	0.403	0.247	0.442	0.306	0.406	0.266
ITM2B	0.304	0.275	0.306	0.277	0.198	0.152	0.213	0.173	0.341	0.312	0.296	0.264
ITM2C	0.313	0.230	0.295	0.211	0.257	0.141	0.258	0.156	0.360	0.275	0.323	0.235
KCMA1	0.287	0.281	0.274	0.268	0.342	0.337	0.325	0.319	0.392	0.391	0.361	0.358
LIN7B	0.379	0.176	0.375	0.171	0.000	0.000	0.000	0.000	0.000	0.000	0.343	0.124
LIPA2	0.374	0.198	0.385	0.209	0.356	0.117	0.346	0.136	0.385	0.201	0.322	0.132
LIPA3	0.239	0.100	0.231	0.091	0.177	0.000	0.167	0.000	0.221	0.075	0.180	0.029
LIPA4	0.577	0.196	0.558	0.174	0.652	0.139	0.608	0.155	0.666	0.268	0.573	0.165
LRFN1	0.438	0.419	0.434	0.414	0.620	0.605	0.585	0.570	0.633	0.622	0.565	0.550
LRFN4	0.552	0.548	0.492	0.486	0.604	0.602	0.508	0.501	0.534	0.528	0.513	0.507
LRRT1	0.361	0.069	0.000	0.000	0.505	0.116	0.000	0.000	0.455	0.151	0.000	0.000
LRRT3	0.487	0.214	0.504	0.230	0.627	0.264	0.611	0.291	0.645	0.364	0.580	0.291
NLGN2	0.328	0.305	0.362	0.341	0.218	0.181	0.178	0.144	0.246	0.219	0.225	0.197
NLGN3	0.537	0.243	0.564	0.270	0.715	0.326	0.621	0.274	0.625	0.319	0.571	0.258
NRX1A	0.742	0.549	0.770	0.577	0.699	0.434	0.634	0.399	0.649	0.442	0.598	0.385
NRX2A	0.520	0.222	0.546	0.247	0.529	0.125	0.423	0.063	0.451	0.134	0.431	0.109
NRX3A	0.672	0.661	0.701	0.692	0.648	0.631	0.544	0.524	0.607	0.593	0.549	0.532
NTRK3	1	1	1	1	0.999	0.985	0.875	0.856	0.861	0.844	0.851	0.833
PRRT1	1	0.777	1	0.775	1	1	1	0.970	1	0.938	1	0.921
PTPRD	0.941	0.895	0.965	0.920	1	0.972	0.916	0.859	0.880	0.829	0.854	0.800
PTPRF	0.518	0.498	0.493	0.471	0.616	0.594	0.536	0.513	0.572	0.554	0.522	0.501
PTPRS	0.872	0.920	0.888	0.936	0.988	1.000	0.874	0.931	0.837	0.886	0.824	0.872
SYT11	0.210	0.251	0.219	0.261	0.285	0.346	0.231	0.282	0.420	0.473	0.359	0.410
T178B	**1**	**1**	**1**	**1**	**1**	**1**	**1**	**1**	**1**	**1**	**1**	**1**
UN13A	0.465	0.244	0.458	0.235	0.352	0.045	0.346	0.077	0.371	0.134	0.321	0.078
UN13C	0.793	0.321	0.776	0.301	0.916	0.283	0.825	0.263	0.779	0.281	0.694	0.185
UNC5A	0.323	0.279	0.276	0.230	0.319	0.260	0.399	0.351	0.000	0.000	0.439	0.398

Table S4 tnR-values of APs related to Figures 5, 6

Antibodies AB#1, anti-TAFA1, RnD Systems, #AF5154
AB#2, anti-TAFA1, RnD Systems, #MAB5154
AB#3, anti-TAFA2, RnD Systems, #4179
IgG, Millipore, #12-370
pre-immune-sera, LifeTein
unrelated AB, anti-MDGA2, RnD Systems, #AF5184

source WT

TAFA1/2	AB#1-vs-IgG#1	AB#1-vs-unrel.AB	AB#1-exp#2-vs-IgG#1	AB#1-exp#2-vs-unrel.AB	TAFA2	AB#3-vs-IgG#1	AB#3-vs-IgG#2	AB#3-vs-IgG#3
	tnR	tnR	tnR	tnR		tnR	tnR	tnR
ASTN1	0.937	0.852	0.951	0.814	ASTN1	0.84	0.68	0.50
BRNP1	0.880	0.856	0.754	0.757	BRNP1	0.61	0.49	0.28
BRNP2	0.857	0.765	0.827	0.806	BRNP2	0.66	0.64	0.60
CBLN1	0.350	0.398	0.587	0.388	CBLN1	0.06	0.33	0.36
KCMA1	1	0.757	1	0.854	KCMA1	0.15	0.15	0.03
NRX1A	0.999	0.657	1	0.770	NRX1A	0.36	0.36	0.13
NRX2A	0.999	0.970	0.709	1	NRX2A	0.65	0.64	0.60
NRX2B	0.999	0.999	1	1	NRX2B	0.90	0.90	0.88
NRX3A	0.439	0.976	1	1	NRX3A	0.86	0.85	0.27
PTPRD	0.810	0.853	0.894	0.833	PTPRD	0.45	0.42	0.22
PTPRS	0.877	0.614	0.981	0.564	PTPRS	0.40	0.18	0.00
T178B	1	0.880	0.842	0.769	T178B	0.60	0.58	0.41
TAFA1	1	1	1	1	TAFA1	0.54	0.55	0.48
TAFA2					TAFA2	1	1	1
XKR4	0.895	0.860	0.544	0.504	XKR4			
XKR6	0.640	0.578	0.433	0.387	XKR6	0.39	0.38	0.33

Table S4 tnR-values of APs related to Figures 5, 6

Antibodies AB#1, anti-NXPH1, RnD, #AF4208
AB#1, anti-NXPH3, RnD, #AF5098
IgG, Millipore (#12-370)

source WT

NXPH1	AB#1-vs-IgG#1	AB#1-exp#2-vs-IgG#1	NXPH3	AB#1-vs-IgG#1	AB#1-exp#2-vs-IgG#1
	tnR	tnR		tnR	tnR
A4			A4	0.495	0.176
CA2D1	0.610	0.400	CA2D1	0.406	0.112
CAC1A	0.580	0.140	CAC1A	0.627	0.362
CACB1	0.530	0.120	CACB1	0.346	0.530
CACB3	0.250	0.190	CACB3	0.546	0.280
CAH10			CAH10	0.473	0.550
CALX	0.380	0.000	CALX	0.530	0.059
CBLN1	0.300	0.220	CBLN1	0.441	0.673
CBLN2	0.390	0.270	CBLN2	0.658	0.705
CBLN4	0.570	0.170	CBLN4	0.497	0.573
CLUS	0.560	0.130	CLUS	0.123	0.358
CSKP	0.480	0.020	CSKP	0.504	0.305
CYTSB			CYTSB	0.660	0.646
DAF1			DAF1	0.531	0.682
GRIK2			GRIK2	0.386	0.367
KCMA1	0.460	0.000	KCMA1	0.622	0.482
LDAF1			LDAF1	0.728	0.572
LRRT1			LRRT1	0.506	0.641
LRRT3			LRRT3	0.326	0.408
MAGI2	0.110	0.060	MAGI2	0.478	0.664
NEGR1	0.520	0.530	NEGR1	0.384	0.831
NMDE1	0.540	0.080	NMDE1	0.385	0.447
NMDE2	0.500	0.000	NMDE2	0.229	0.425
NMDZ1	0.480	0.040	NMDZ1	0.418	0.499
NPTX1	0.190	0.050	NPTX1	0.405	0.407
NRX1A	0.680	0.740	NRX1A	1	1
NRX2A	0.640	0.600	NRX2A	0.756	0.513
NRX3A	0.750	0.760	NRX3A	0.296	0.719
NXPH1	1	1	NXPH1		
NXPH3			NXPH3	1	1
PKD2			PKD2	0.170	0.731
PLPR2			PLPR2	0.496	0.592
PLPR4	0.310	0.170	PLPR4	0.533	0.775
PTPRD	0.370	0.160	PTPRD	0.412	0.513
PTPRS	0.520	0.310	PTPRS	0.522	0.637
T178B			T178B	0.530	0.469
TM240			TM240	0.740	0.750
XKR4			XKR4	0.504	0.244
XKR6	0.560	0.530	XKR6	0.446	0.354

Table S4 tnR-values of APs related to Figures 5, 6

Antibodies AB#1, anti-LRRT4, RnD Systems, #AF5377
AB#2, anti-LRRT4, AbFrontier, #C1
AB#3, anti-LRRT4, Neuromab, #75-261
IgG, Millipore, #12-370

source WT
LRRT4 KO

**LRRT4**	**AB#1-vs-IgG#1**	**AB#1-vs-IgG#2**	**AB#1-vs-IgG#3**	**AB#2-vs-IgG#1**	**AB#2-vs-IgG#2**	**AB#2-vs-IgG#3**	**AB#2-exp2-vs-IgG#1**	**AB#2-exp2-vs-IgG#2**	**AB#2-exp2-vs-IgG#3**	**AB#3-vs-IgG#1**	**AB#3-vs-IgG#2**	**AB#3-vs-IgG#3**	**AB#3-vs-AB#3KO**
	**tnR**	**tnR**	**tnR**	**tnR**	**tnR**	**tnR**	**tnR**	**tnR**		**tnR**	**tnR**	**tnR**	**tnR**
CLUS	0.309	0.000	0.200	0.648	0.115	0.543	0.351	0.000	0.243	0.255	0.000	0.162	0.000
DLG3	0.332	0.000	0.457	0.479	0.000	0.585	0.186	0.000	0.313	0.408	0.000	0.514	0.134
GPC1	0.558	0.245	0.433	0.729	0.454	0.614	0.627	0.321	0.503	0.695	0.426	0.584	0.538
GPC4	0.395	0.217	0.332	0.541	0.386	0.482	0.415	0.242	0.353	0.482	0.330	0.426	0.418
GPC5	0.000	0.000	0.000	0.506	0.362	0.453	0.320	0.158	0.266	0.397	0.256	0.348	0.591
GRIA1	0.461	0.023	0.466	0.617	0.232	0.617	0.565	0.135	0.566	0.294	0.000	0.302	0.414
GRIA2	0.441	0.077	0.633	0.626	0.306	0.791	0.574	0.218	0.759	0.304	0.000	0.473	0.307
GRIA3	0.466	0.087	0.517	0.633	0.299	0.673	0.613	0.242	0.659	0.333	0.007	0.380	0.460
GSG1L	0.768	0.448	0.627	0.962	0.681	0.833	0.994	0.681	0.849	0.602	0.327	0.485	0.557
LRRT3	0.188	0.120	0.221	0.500	0.440	0.521	0.153	0.086	0.186	0.237	0.178	0.264	0.319
LRRT4	**1**	**1**	**1**	**1**	**1**	**1**	**1**	**1**	**1**	**1**	**1**	**1**	**1**
MARK1	0.439	0.000	0.296	0.692	0.222	0.559	0.386	0.000	0.248	0.385	0.000	0.263	0.000
NRX1A	0.424	0.022	0.497	0.607	0.253	0.667	0.444	0.049	0.514	0.508	0.162	0.568	0.329
NRX2A	0.422	0.000	0.289	0.562	0.167	0.441	0.380	0.000	0.251	0.466	0.081	0.351	0.384
NRX3A	0.309	0.000	0.254	0.499	0.075	0.446	0.368	0.000	0.313	0.421	0.008	0.371	0.455
PTPRD	0.340	0.001	0.230	0.557	0.259	0.455	0.347	0.015	0.240	0.413	0.122	0.317	0.376
PTPRS	0.327	0.000	0.355	0.559	0.168	0.578	0.287	0.000	0.316	0.300	0.000	0.325	0.480
SHSA7	0.420	0.000	0.307	0.732	0.153	0.624	0.399	0.000	0.288	0.302	0.000	0.207	0.142
T178B	0.268	0.000	0.133	0.467	0.183	0.342	0.414	0.097	0.277	0.400	0.122	0.279	0.423
TEFF1	0.000	0.000	0.000	0.558	0.283	0.429	0.395	0.088	0.256	0.440	0.171	0.317	0.464
TEFF2	0.000	0.000	0.000	0.595	0.297	0.446	0.448	0.116	0.287	0.548	0.258	0.404	0.420
SDC4	0.000	0.000	0.000	0.497	0.235	0.393	0.179	0.000	0.073	0.423	0.168	0.324	0.461

Table S4 tnR-values of APs related to Figures 5, 6

Antibodies
AB#1, anti-LRRT2, NeuroMab, #75-364
AB#2, anti-LRRT1/3, Millipore, #ABN642
IgG, Millipore, #12-370
pre-immune-sera, LifeTein

**LRRT1/2/3**	**AB#1-vs-IgG#1**	**AB#1-vs-IgG#2**	**AB#2-vs-IgG#1**	**AB#2-vs-IgG#2**
	**tnR**	**tnR**	**tnR**	**tnR**
GPC1	0.39	0.27	0.12	0.75
LRRT1	0.00	0.00	**1.00**	**1.00**
LRRT2	**1**	**1**	0.00	0.00
LRRT3	0.00	0.00	**0.29**	**0.74**
NRX1A	0.36	0.31	0.26	0.45
NRX2A	0.21	0.09	0.13	0.43
NRX3A	0.48	0.40	0.00	0.50
PTPRF	0.00	0.00	0.22	0.46
PTPRS	0.20	0.00	0.27	0.68

Table S4 tnR-values of APs related to Figures 5, 6

Antibodies AB#1, anti-XKR6, AmsBio, #RB3961
AB#2, anti-XKR6, AmsBio, #RB3962
AB#3, anti-XKR6, SignalwayAntibody, #42864
AB#4, anti-XKR6, Sino Biologicals, #206331
AB#5, anti-XKR6, LifeTein, #RB4229

IgG, Millipore, #12-370
source WT

**XKR6**	**AB#1-vs-IgG#1**	**AB#1-vs-IgG#2**	**AB#2-vs-IgG#1**	**AB#2-vs-IgG#2**	**AB#3-vs-IgG#1**	**AB#4-vs-IgG#1**	**AB#5-vs-IgG#2**
	**tnR**	**tnR**	**tnR**	**tnR**	**tnR**	**tnR**	**tnR**
C1QI3	0.00	0.27	0.09	0.37	0.78	0.62	0.44
CAH10	0.47	0.47	0.53	0.53	0.51	0.41	0.76
LIN7B	0.53	0.51	0.58	0.56	0.58	0.44	0.78
NRX1A	0.56	0.55	0.54	0.54	0.47	0.28	0.73
NRX2A	0.31	0.32	0.32	0.32	0.29	0.00	0.46
NRX3A	0.52	0.52	0.50	0.50	0.47	0.42	0.80
PTPRD	0.38	0.36	0.34	0.32	0.38	0.19	0.44
PTPRS	0.48	0.30	0.43	0.25	0.40	0.15	0.19
T178B	0.21	0.20	0.29	0.28	0.21	0.00	0.00
XKR6	**1**	**1**	**1**	**1**	**1**	**1**	**1**

Table S4 tnR-values of APs related to Figures 5, 6

Antibodies AB#1, anti-GRM4, Bioworld, #BS1144
AB#2, anti-GRM4, Abcam, #ab184302
AB#3, anti-GRM4, Abcam, #ab53088
IgG, Millipore, #12-370

source WT cerebellum

**GRM4**	**AB#1-vs-IgG#1**	**AB#2-vs-IgG#1**	**AB#3-vs-IgG#1**
	**tnR**	**tnR**	**tnR**
CAC1A	0.266	0.539	0.385
CBLN1	0.362	0.505	0.443
CBLN3	0.800	0.841	0.819
GPM6A	0.652	0.736	0.655
GRM4	1	1	1
NRX1A	0.694	0.707	0.752
NRX2A	0.362	0.473	0.504
NRX2B	0.483	0.548	0.563
PTPRS	0.000	0.345	0.292
SNP25	0.488	0.513	0.495
STX1B	0.557	0.658	0.598
STXB1	0.359	0.504	0.393
SV2A	0.491	0.600	0.525
SYT1	0.620	0.681	0.611
SYT3	0.682	0.805	0.752
SYT9	0.471	0.598	0.553
T132A	0.393	0.561	0.468
VGLU1	0.517	0.645	0.579

Table S4 tnR-values of APs related to Figures 5, 6

Antibodies AB#1, anti-HA, Roche, #11867423001
AB#2, anti-HA, Thermo Fisher, #26183
IgG, Millipore (#12-370)

source WT
Knock-In NLGN1-HA

**NLGN1**	**AB#1-vs-IgG**	**AB#2-vs-HA(WT)**	**AB#1-exp2-vs-IgG**	**AB#2-exp2-vs-HA(WT)**
	**tnR**	**tnR**	**tnR**	**tnR**
GBRA1	1	1	1	1
GBRA2	0.927	0.970	1	1
GBRA3	0.714	0.818	0.858	1
GBRB1	0.036	0.837	0.558	1
GBRB2	0.848	0.711	0.388	0.821
GBRG1	0.734	0.649	0.711	0.753
GBRG2	1	1	1	1
GEPH	0.567	0.127	0.600	0.307
GLRA1	0.603	0.642	0.813	0.141
GLRA3	0.805	0.743	0.657	1
GLRB	1	1	1	1
GLRB	0.921	0.873	1	1
LHPL4	0.889	0.659	0.880	0.990
LHPL4	0.858	0.980	1	1
**NLGN1**	**1**	**1**	**1**	**1**
NLGN2	0.860	1	0.715	1
NLGN3	0.868	1	0.541	1
NLGN4	1	1	0.992	1
S12A5	0.910	0.988	0.669	1

Table S4 tnR-values of APs related to Figures 5, 6

Antibodies AB#1, anti-NLGN2, Synaptic Systems, #129511
AB#2, anti-NLGN3, NeuroMab, #75-158
IgG, Millipore (#12-370)

source WT

**NLGN2_3**	**NLGN2-vs-IgG**	**NLGN2-vs-IgG#2**	**NLGN3-vs-IgG**	**NLGN3-vs-IgG#2**
	**tnR**	**tnR**	**tnR**	**tnR**
NLGN1	0.340	0.459	0.302	0.248
NLGN2	**1**	**1**	0.368	0.249
NLGN3	0.598	0.715	**1**	**1**
GBRA1	0.396	0.604	0.493	0.422
GBRA2	0.475	0.783	0.540	0.495
GBRA3	0.565	0.677	0.475	0.430
GBRA5	0.372	0.490	0.315	0.270
GBRA6	0.185	0.480	0.000	0.000
GBRB1	0.598	0.714	0.491	0.445
GBRB2	0.458	0.610	0.608	0.334
GBRB3	0.442	0.827	0.619	0.567
GBRG2	0.516	0.692	0.655	0.402
LHPL4	0.744	0.854	0.519	0.481
PTPRS	0.043	0.227	0.000	0.000

Table S4 tnR-values of APs related to Figures 5, 6

Antibodies AB#1, anti-GLRA1, RnD Systems, #PPS009
IgG, Millipore (#12-370)

source WT

GLRA1	AB#1-vs-IgG	AB#1-vs-IgG#2
	tnR	tnR
NLGN1	0.325	0.391
NLGN2	0.526	0.433
NLGN3	0.471	0.508
NLGN4	0.458	0.491
GBRA1	0.035	0.274
GBRA2	0.051	0.401
GBRA3	0.379	0.432
GBRB1	0.255	0.343
GBRB2	0.002	0.189
GBRB3	0.044	0.482
GBRG2	0.085	0.280
GLRA1	**1**	**1**
GLRA2	0.291	0.686
GLRA3	0.730	0.710
GLRB	0.971	0.746
GEPH	0.288	0.381

Table S4 tnR-values of APs related to Figures 5, 6

Antibodies AB#1, anti-GBRG2, Synaptic Systems, #224003
AB#2, anti-GBRA1, Synaptic Systems, #224203

IgG, Millipore (#12-370)

source WT

GBRA1/G2	AB#1-vs-IgG#1 tnR	AB#1-vs-IgG#2 tnR	AB#2-vs-IgG#1 tnR	AB#2-vs-IgG#2 tnR
GBRA1	1	1	**1**	**1**
GBRA2	0.735	0.717	0.822	0.819
GBRA3	0.895	0.875	1	1
GBRA4	0.000	0.000	0.826	0.804
GBRA5	0.768	0.712	0.831	0.768
GBRA6	0.463	0.355	0.752	0.676
GBRB1	0.736	0.676	1	1
GBRB2	0.910	0.879	1	1
GBRB3	1	1	1	1
GBRD	0.000	0.000	0.921	0.433
GBRG1	0.000	0.000	0.942	0.883
GBRG2	**1**	**1**	1	1
GBRG3	0.000	0.000	0.382	0.219
GEPH	0.213	0.201	0.000	0.000
LHPL3	0.505	0.412	0.597	0.488
LHPL4	0.659	0.578	0.956	0.921
MDGA2	0.653	0.587	0.851	0.810
NACHO	0.230	0.052	0.447	0.252
NLGN2	0.810	0.763	1	1
NLGN3	0.704	0.635	0.960	0.932
NLGN4	0.564	0.471	0.834	0.776
NRX3A	0.000	0.000	0.331	0.150

Table S4 tnR-values of APs related to Figures 5, 6

Antibodies AB#1, anti-RTN4R, RnD Systems, #AF1440
IgG, Millipore (#12-370)

source WT

**RTN4R**	**AB#1-vs-IgG**<br>**tnR**	**AB#1-vs-IgG#2**<br>**tnR**	**AB#1-vs-IgG#3**<br>**tnR**
BSN	0.740	0.610	0.610
CAC1B	0.520	0.510	0.480
CAC1E	0.590	0.520	0.450
CSKI1	0.800	0.730	0.630
CYFP1	0.670	0.620	0.540
LRFN1	0.700	0.700	0.480
NHSL1	0.790	0.780	0.680
NRX1A	0.630	0.620	0.510
NRX3A	0.650	0.650	0.310
PTPRD	0.450	0.440	0.330
PTPRS	0.450	0.320	0.220
RTN4R	**1**	**1**	**1**
SEM6D	0.670	0.660	0.640
SHSA6	0.700	0.600	0.490
TNIK	0.530	0.480	0.420

Table S4 tnR-values of APs related to Figures 5, 6

Antibodies AB#1, anti-SYT11, Synaptic Systems, #270003
IgG, Millipore (#12-370)

source WT
SYT11 KO

data published in Trovo et al. 2024 (PMID: 38698221)

**SYT11**	**AB#1-vs-IgG**	**AB#1-exp2-vs-IgG#2**	**AB#1-exp2-vs-KO**
	**tnR**	**tnR**	**tnR**
A4	0.207	0.277	0.293
APLP2	0.266	0.349	0.372
ASTN1	0.299	0.283	0.321
ATRN	0.528	0.500	0.540
ATRN1	0.446	0.408	0.440
BTBDH	0.010	0.361	0.385
CAC1B	0.382	0.185	0.146
GABR1	0.174	0.203	0.285
GABR2	0.153	0.279	0.265
GPC1	0.525	0.417	0.179
GRM2	0.000	0.313	0.336
ITM2B	0.501	0.493	0.644
ITM2C	0.278	0.475	0.514
KCD16	0.165	0.259	0.344
KCTD8	0.294	0.230	0.371
KIF1A	0.396	0.288	0.297
KIF1B	0.384	0.261	0.276
MRCKA	0.132	0.339	0.370
NRX1A	0.259	0.303	0.300
NSG1	0.251	0.318	0.340
NTRK2	0.681	0.809	0.902
PCD17	0.242	0.364	0.389
PTPRS	0.155	0.356	0.386
RFTN1	0.559	0.400	0.448
SNPH	0.000	0.241	0.253
SYT11	**1**	**1**	**1**
T132A	0.409	0.528	0.579
TRPV2	0.418	0.433	0.453
VGLU1	0.550	0.850	0.940

Table S4 tnR-values of APs related to Figures 5, 6

Antibodies	AB#1, anti-CAC1A, FrontierInstitutes, #Af810
	AB#2, anti-CAC1E, Synaptic Systems, #152403
	IgG, Millipore (#12-370)
source	WT
	CAC1A KO
	CAC1E KO

**CAC1A/E**	**AB#1-vs-KO tnR**	**AB#1-vs-KO tnR**
CAC1B	0.073	0.080
CAC1A	**1**	0.000
CAC1E	0.156	**1**
CACB1	0.425	0.444
CACB2	0.490	0.494
CACB3	0.541	0.261
CACB4	0.468	0.340
CA2D1	0.462	0.356
KCMA1	0.399	0.215
PTPRS	0.369	0.259
PTPRD	0.386	0.276
T178B	0.373	0.209
NRX1A	0.291	0.096
NRX2A	0.408	0.315
NRX3A	0.508	0.287
RIMB1	0.000	0.317
RIMB2	0.597	0.000

AB#1 and #2 were selected based on their abundant co-purification of the NRX-T178-PTPR core-module
Interactors consistent among AB#1 and #2 are listed

Table S4 tnR-values of APs related to Figures 5, 6

Antibodies AB#1, anti-A4, Abgent, #ABIN1741750
AB#2, anti-A4,Sigma, #A8717
IgG, Millipore (#12-370)

source WT
A4 KO

A4/APLP2	AB#1-vs-KO tnR	AB#1-IgG tnR	AB#2-vs-KO tnR	AB#2-IgG tnR
A4	**1**	**1**	**1**	**1**
APLP2	0.380	0.454	0.493	0.315
C1QL3	0.109	0.406	0.390	1000
CADM3	0.083	0.369	0.311	0.821
CSTN1	0.823	0.567	0.439	1000
CSTN3	0.702	0.408	0.417	0.681
EPHA4	0.504	0.000	0.399	1000
GABR1	0.676	0.054	0.209	0.640
ITM2B	0.694	0.969	0.829	1000
ITM2C	0.686	1000	0.643	1000
L1CAM	0.797	0.202	0.451	0.895
LRRN3	0.546	0.089	0.338	0.893
NRX1A	0.468	0.233	0.232	0.357
NSG1	0.833	0.484	0.430	1000
NSG2	0.723	0.400	0.498	1000
PLXA4	0.594	0.070	0.163	0.891
PTPRD	0.457	0.104	0.230	0.106
S6A17	0.439	0.467	0.176	0.519
SEM7A	0.774	0.230	0.442	1000
STMN2	0.828	0.866	0.362	0.941
STMN3	0.790	0.746	0.292	1000
STMN4	0.564	0.586	0.247	0.715
SYT11	0.940	0.467	0.525	1000
T132A	0.669	0.351	0.404	1000

Table S4 tnR-values of APs related to Figures 5, 6

Antibodies AB#1, anti-NOE1, Ref. Boudkkazi et al.
AB#2, anti-NOE1, Ref. Boudkkazi et al.
AB#3, anti-NOE1, RnD Systems
IgG, Millipore (#12-370)

source WT
NOE1 KO

data published in Boudkkazi et al. 2023 (PMID: 37591201)

**NOE1**	**AB#1-vs-KO**	**AB#2-vs-KO**	**AB#3-vs-KO**
	**tnR**	**tnR**	**tnR**
NOE1	**1**	**1**	**1**
NOE2	1000	0.650	0.690
NOE3	0.940	0.510	0.708
NRN1	1000	0.752	1000
GRIA1	0.748	0.473	0.464
GRIa2	0.728	0.388	0.346
GRIA3	0.878	0.496	0.598
GRIA4	0.929	0.733	0.734
CCG2	0.825	0.434	0.323
CCG4	1000	0.442	0.000
CCG7	0.554	0.273	0.000
CCG3	0.501	0.171	0.000
VWC2	0.766	0.313	0.000
VWC2L	0.711	0.312	0.000
PRRT1	0.787	0.235	0.000
SHSA9	0.522	0.237	0.000
CNIH2	0.659	0.306	0.000
SE6L1	1000	0.297	1000
NRX1A	0.450	0.215	0.674
HPLN4	0.405	0.261	0.815
CAH10	0.359	0.147	0.157
RENR	1000	0.883	1000
SHPS1	1000	0.945	0.000

Table S4 tnR-values of APs related to Figures 5, 6

Antibodies AB#1, anti-GSG1L, (aa304-322, Perozzo et al.)
AB#2, anti-GSG1L, (aa257-278, Perozzo et al.)
AB#3, anti-GSG1L, (aa287-308, Perozzo et al.)
IgG, Millipore (#12-370)

source WT
GSG1L KO

data published in Perozzo et al. 2023 (PMID: 37884493)

GSG1L	AB#1-vs-KO tnR	AB#2-vs-KO tnR	AB#3-vs-KO tnR
CCG2	0.601	0.506	0.450
CCG3	0.587	0.540	0.455
CCG8	0.532	0.371	0.349
CNIH2	0.527	0.461	0.314
GRIA1	0.742	0.717	0.755
GRIA2	0.708	0.661	0.669
GRIA3	0.671	0.629	0.558
GRIA4	0.540	0.522	0.572
GSG1L	**1**	**1**	**1**
LRRT4	0.485	0.583	0.478
PRRT1	0.484	0.315	0.595
SHSA9	0.538	0.394	0.403

Supplementary Table 5

Resource Table – Antibodies used in Figs.1-6

Antibodies			
**TARGET**	**SOURCE**	**IDENTIFIER**	**Experiments**
Rabbit polyclonal anti-NRX1	Millipore	#ABN161-I	Fig. 1, 2, 3, 6
Rabbit polyclonal anti-NRX1-3	this study	#RB2987	Fig. 1, 2, 4, 6
Rabbit polyclonal anti-NRX1-3	this study	#3-4-rb1	Fig. 1, 6
Rabbit polyclonal anti-NRX1	Synaptic Systems	#175103	Fig. 1, 6
Rabbit polyclonal anti-NRX1-3	Synaptic Systems	#175003	Fig. 1, 6
Sheep polyclonal anti-NRX1	RnD Systems	#AF4524	Fig. 1, 6
Rabbit polyclonal anti-NRX2	this study	#RB4227	Fig. 1, 6
Rabbit polyclonal anti-NRX2	this study	#RB4226	Fig. 1, 6
Rabbit polyclonal anti-NRX2	this study	#RB4225	Fig. 1, 6
Rabbit polyclonal anti-NRX2	Abcam	#ab34245	Fig. 1, 6
Rabbit polyclonal anti-NRX3	this study	#3-1	Fig. 1, 6
Rabbit polyclonal anti-NRX3	this study	#3-2-rb1	Fig. 1, 6
Rabbit polyclonal anti-NRX3	this study	#3-2-rb2	Fig. 1, 6
Rabbit polyclonal anti-NRX3	RnD Systems	#AF5269	Fig. 1, 6
Rabbit polyclonal anti-T178B	this study	#RB4235	Fig. 2, 3, 6
Rabbit polyclonal anti-T178B	this study	#RB3967	Fig. 2, 6
Rat monoclonal anti-HA	Roche	#11867423001	Fig. 1
Mouse monoclonal anti-HA	Thermo Fisher	#26183	Fig. 1, 2
Goat polyclonal anti-PTPRS	RnD Systems	#AF3430	Fig. 2, 3, 5
Mouse monoclonal anti-PTPRS	MediMabs	#MM-0020-P	Fig. 5, 6
Goat polyclonal anti-PTPRS	Invitrogen	#PA5-47487	Fig. 5, 6
Mouse monoclonal anti-PTPR	NeuroMab	#75-194	Fig. 2, 3
Mouse monoclonal anti-CAH10	RnD Systems	#MAB2189	Fig. 5, 6
Rabbit polyclonal anti-CAH10	Montoliu-Gaya, L[73]	#Hadlai	Fig. 5, 6
Rabbit polyclonal anti-CAH10	Montoliu-Gaya, L[73]	#Habeba	Fig. 5, 6
Guinea pig polyclonal anti-CAC1A	Frontier Institute	#GP-Af810	Fig. 6
Rabbit polyclonal anti-CAC1E	Alomone	#ACC-006	Fig. 6
Goat polyclonal anti-TAFA1	RnD Systems	#AF5154	Fig. 5, 6
Sheep polyclonal anti-TAFA2	RnD Systems	#AF4179	Fig. 5, 6
Sheep polyclonal anti-NXPH1	RnD Systems	#AF4208	Fig. 5, 6
Goat polyclonal anti-NXPH3	RnD Systems	#AF5098	Fig. 5, 6
Mouse monoclonal anti-LRRT4	NeuroMab	#75-261	Fig. 5, 6
Rabbit polyclonal anti-LRRT4	this study	#C1	Fig. 5, 6
Sheep polyclonal anti-LRRT4	RnD Systems	#AF5377	Fig. 5, 6
Rabbit polyclonal anti-A4	Abgent	#ABIN1741750	Fig. 6
Rabbit polyclonal anti-A4	Sigma	#A8717	Fig. 6
Rabbit polyclonal anti-GBRA1	Synaptic Systems	#224203	Fig. 6
Rabbit polyclonal anti-GBRG2	Synaptic Systems	#224003	Fig. 6
Goat polyclonal anti-RTN4R	RnD Systems	#AF1440	Fig. 6
Rabbit polyclonal anti-LRRT1	Millipore	#ABN642	Fig. 6
Mouse monoclonal anti-LRRT2	NeuroMab	#75-364	Fig. 6
Rabbit polyclonal anti-GRM4	Abcam	#ab53088	Fig. 6
Rabbit polyclonal anti-GRM4	Abcam	#ab184302	Fig. 6
Rabbit polyclonal anti-GRM4	Bioworld	#BS1144	Fig. 6
Mouse monoclonal anti-NLGN2	Synaptic Systems	#129511	Fig. 6

Mouse monoclonal anti-NLGN3	NeuroMab	#75-158	Fig. 6
Rabbit polyclonal anti-SYT11	Synaptic Systems	#270003	Fig. 6
Rabbit polyclonal anti-XKR6	This study	#RB3961	Fig. 6
Rabbit polyclonal anti-XKR6	This study	#RB3962	Fig. 6
Rabbit polyclonal anti-XKR6	Signalway	#42864	Fig. 6
Goat polyclonal anti-VGLU1	Frontier Institute	#MSFR106210	Fig. 4
Guinea pig polyclonal anti-VIAAT	Frontier Institute	#MSFR106150	Fig. 4
Mouse monoclonal anti-V5	BioRad	#MCA1360	Fig. 2
Mouse monoclonal anti-FLAG	Thermo Scientific	#MA1-91878	Fig. 2
