# Supplementary material for: Ternary Neurexin-T178-PTPR complexes represent a pre-synaptic core-module of neuronal synapse organization
Source: Nat Commun. 2026 Sep 5;17:9509. doi: 10.1038/s41467-026-77377-4 (PMC13546301; doi:10.1038/s41467-026-77377-4)
Supplement: Supplementary file 2 [file 41467_2026_77377_MOESM2_ESM.pdf]

nature portfolio

Corresponding author(s): Bernd Fakler, Jochen Schwenk

Last updated by author(s): 24. July, 2026

Reporting Summary

Nature Portfolio wishes to improve the reproducibility of the work that we publish. This form provides structure for consistency and transparency in reporting. For further information on Nature Portfolio policies, see our Editorial Policies and the Editorial Policy Checklist.

Statistics

For all statistical analyses, confirm that the following items are present in the figure legend, table legend, main text, or Methods section.

n/a	Confirmed	
☐	☒	The exact sample size (*n*) for each experimental group/condition, given as a discrete number and unit of measurement
☐	☒	A statement on whether measurements were taken from distinct samples or whether the same sample was measured repeatedly
☐	☒	The statistical test(s) used AND whether they are one- or two-sided<br>*Only common tests should be described solely by name; describe more complex techniques in the Methods section.*
☒	☐	A description of all covariates tested
☒	☐	A description of any assumptions or corrections, such as tests of normality and adjustment for multiple comparisons
☐	☒	A full description of the statistical parameters including central tendency (e.g. means) or other basic estimates (e.g. regression coefficient) AND variation (e.g. standard deviation) or associated estimates of uncertainty (e.g. confidence intervals)
☐	☒	For null hypothesis testing, the test statistic (e.g. *F*, *t*, *r*) with confidence intervals, effect sizes, degrees of freedom and *P* value noted<br>*Give P values as exact values whenever suitable.*
☒	☐	For Bayesian analysis, information on the choice of priors and Markov chain Monte Carlo settings
☒	☐	For hierarchical and complex designs, identification of the appropriate level for tests and full reporting of outcomes
☒	☐	Estimates of effect sizes (e.g. Cohen's *d*, Pearson's *r*), indicating how they were calculated

Our web collection on statistics for biologists contains articles on many of the points above.

Software and code

Policy information about availability of computer code

Data collection	The individual datasets were acquired with the following commercially available software (in combination with respective hardware)<br>msconvert v3.0.11098 for processing of primary MS data (obtained with Orbitrap Elite or QExactive HF-X mass spectrometer)<br>Built-in software of TEM microscope (Jeol JEM 2100 Plus) for EM
Data analysis	msconvert v3.0.11098 for processing of primary MS data<br>MaxQuant 1.6.3 for callibration and quantification of MS data<br>Mascot 2.7 for database search<br>BELKI software suite (https://github.com/phys2/belki)<br>pClamp 10 software and IGOR Pro 9 for evaluation of electrophysiological data<br>MultiClamp and Clampex 10.7.0.3 softwares (Molecular Devices)<br>Clampfit 11.4.2 software (Molecular Devices)<br>GraphPad Prism 10.2.0 software<br>Python software (pandas library (10.5281/zenodo.3509134), BOKEH library (http://www.bokeh.pydata.org), UMAP (https://github.com/lmcinnes/umap))

For manuscripts utilizing custom algorithms or software that are central to the research but not yet described in published literature, software must be made available to editors and reviewers. We strongly encourage code deposition in a community repository (e.g. GitHub). See the Nature Portfolio guidelines for submitting code & software for further information.

Data

Policy information about availability of data

All manuscripts must include a data availability statement. This statement should provide the following information, where applicable:

- Accession codes, unique identifiers, or web links for publicly available datasets
- A description of any restrictions on data availability
- For clinical datasets or third party data, please ensure that the statement adheres to our policy

The mass spectrometry data have been deposited to the ProteomeXchange Consortium via the PRIDE partner repository with the dataset identifier PXD054017, and are publicly available as of the date of publication.

Research involving human participants, their data, or biological material

Policy information about studies with human participants or human data. See also policy information about sex, gender (identity/presentation), and sexual orientation and race, ethnicity and racism.

Reporting on sex and gender	*Use the terms sex (biological attribute) and gender (shaped by social and cultural circumstances) carefully in order to avoid confusing both terms. Indicate if findings apply to only one sex or gender; describe whether sex and gender were considered in study design; whether sex and/or gender was determined based on self-reporting or assigned and methods used.*<br>*Provide in the source data disaggregated sex and gender data, where this information has been collected, and if consent has been obtained for sharing of individual-level data; provide overall numbers in this Reporting Summary. Please state if this information has not been collected.*<br>*Report sex- and gender-based analyses where performed, justify reasons for lack of sex- and gender-based analysis.*
Reporting on race, ethnicity, or other socially relevant groupings	*Please specify the socially constructed or socially relevant categorization variable(s) used in your manuscript and explain why they were used. Please note that such variables should not be used as proxies for other socially constructed/relevant variables (for example, race or ethnicity should not be used as a proxy for socioeconomic status).*<br>*Provide clear definitions of the relevant terms used, how they were provided (by the participants/respondents, the researchers, or third parties), and the method(s) used to classify people into the different categories (e.g. self-report, census or administrative data, social media data, etc.)*<br>*Please provide details about how you controlled for confounding variables in your analyses.*
Population characteristics	*Describe the covariate-relevant population characteristics of the human research participants (e.g. age, genotypic information, past and current diagnosis and treatment categories). If you filled out the behavioural & social sciences study design questions and have nothing to add here, write "See above."*
Recruitment	*Describe how participants were recruited. Outline any potential self-selection bias or other biases that may be present and how these are likely to impact results.*
Ethics oversight	*Identify the organization(s) that approved the study protocol.*

Note that full information on the approval of the study protocol must also be provided in the manuscript.

Field-specific reporting

Please select the one below that is the best fit for your research. If you are not sure, read the appropriate sections before making your selection.

☒ Life sciences ☐ Behavioural & social sciences ☐ Ecological, evolutionary & environmental sciences

For a reference copy of the document with all sections, see nature.com/documents/nr-reporting-summary-flat.pdf

Life sciences study design

All studies must disclose on these points even when the disclosure is negative.

Sample size	No statistical method was used to predetermine sample size. The sample size was determined based on previous experience with the respective types of experiments; detailed numbers of experiments are indicated in figures and legends.
Data exclusions	No relevant data were excluded.
Replication	All experiments were performed in replicas and detailed in the text, methods and figure legends. Images from Western Blots, EM and immunostainings are representative images.
Randomization	For proteomics and electrophysiology the animals were allocated to experimental groups according to their genotype.
Blinding	Blinding was not used in this study.

Behavioural & social sciences study design

All studies must disclose on these points even when the disclosure is negative.

Study description	*Briefly describe the study type including whether data are quantitative, qualitative, or mixed-methods (e.g. qualitative cross-sectional, quantitative experimental, mixed-methods case study).*
Research sample	*State the research sample (e.g. Harvard university undergraduates, villagers in rural India) and provide relevant demographic information (e.g. age, sex) and indicate whether the sample is representative. Provide a rationale for the study sample chosen. For studies involving existing datasets, please describe the dataset and source.*
Sampling strategy	*Describe the sampling procedure (e.g. random, snowball, stratified, convenience). Describe the statistical methods that were used to predetermine sample size OR if no sample-size calculation was performed, describe how sample sizes were chosen and provide a rationale for why these sample sizes are sufficient. For qualitative data, please indicate whether data saturation was considered, and what criteria were used to decide that no further sampling was needed.*
Data collection	*Provide details about the data collection procedure, including the instruments or devices used to record the data (e.g. pen and paper, computer, eye tracker, video or audio equipment) whether anyone was present besides the participant(s) and the researcher, and whether the researcher was blind to experimental condition and/or the study hypothesis during data collection.*
Timing	*Indicate the start and stop dates of data collection. If there is a gap between collection periods, state the dates for each sample cohort.*
Data exclusions	*If no data were excluded from the analyses, state so OR if data were excluded, provide the exact number of exclusions and the rationale behind them, indicating whether exclusion criteria were pre-established.*
Non-participation	*State how many participants dropped out/declined participation and the reason(s) given OR provide response rate OR state that no participants dropped out/declined participation.*
Randomization	*If participants were not allocated into experimental groups, state so OR describe how participants were allocated to groups, and if allocation was not random, describe how covariates were controlled.*

Ecological, evolutionary & environmental sciences study design

All studies must disclose on these points even when the disclosure is negative.

Study description	*Briefly describe the study. For quantitative data include treatment factors and interactions, design structure (e.g. factorial, nested, hierarchical), nature and number of experimental units and replicates.*
Research sample	*Describe the research sample (e.g. a group of tagged Passer domesticus, all Stenocereus thurberi within Organ Pipe Cactus National Monument), and provide a rationale for the sample choice. When relevant, describe the organism taxa, source, sex, age range and any manipulations. State what population the sample is meant to represent when applicable. For studies involving existing datasets, describe the data and its source.*
Sampling strategy	*Note the sampling procedure. Describe the statistical methods that were used to predetermine sample size OR if no sample-size calculation was performed, describe how sample sizes were chosen and provide a rationale for why these sample sizes are sufficient.*
Data collection	*Describe the data collection procedure, including who recorded the data and how.*
Timing and spatial scale	*Indicate the start and stop dates of data collection, noting the frequency and periodicity of sampling and providing a rationale for these choices. If there is a gap between collection periods, state the dates for each sample cohort. Specify the spatial scale from which the data are taken*
Data exclusions	*If no data were excluded from the analyses, state so OR if data were excluded, describe the exclusions and the rationale behind them, indicating whether exclusion criteria were pre-established.*
Reproducibility	*Describe the measures taken to verify the reproducibility of experimental findings. For each experiment, note whether any attempts to repeat the experiment failed OR state that all attempts to repeat the experiment were successful.*
Randomization	*Describe how samples/organisms/participants were allocated into groups. If allocation was not random, describe how covariates were controlled. If this is not relevant to your study, explain why.*
Blinding	*Describe the extent of blinding used during data acquisition and analysis. If blinding was not possible, describe why OR explain why blinding was not relevant to your study.*

Did the study involve field work? ☐ Yes ☐ No

Field work, collection and transport

Field conditions	*Describe the study conditions for field work, providing relevant parameters (e.g. temperature, rainfall).*
Location	*State the location of the sampling or experiment, providing relevant parameters (e.g. latitude and longitude, elevation, water depth).*
Access & import/export	*Describe the efforts you have made to access habitats and to collect and import/export your samples in a responsible manner and in compliance with local, national and international laws, noting any permits that were obtained (give the name of the issuing authority, the date of issue, and any identifying information).*
Disturbance	*Describe any disturbance caused by the study and how it was minimized.*

Reporting for specific materials, systems and methods

We require information from authors about some types of materials, experimental systems and methods used in many studies. Here, indicate whether each material, system or method listed is relevant to your study. If you are not sure if a list item applies to your research, read the appropriate section before selecting a response.

Materials & experimental systems

n/a	Involved in the study	
☐	☒	Antibodies
☐	☒	Eukaryotic cell lines
☒	☐	Palaeontology and archaeology
☐	☒	Animals and other organisms
☒	☐	Clinical data
☒	☐	Dual use research of concern
☒	☐	Plants

Methods

n/a	Involved in the study	
☒	☐	ChIP-seq
☒	☐	Flow cytometry
☒	☐	MRI-based neuroimaging

Antibodies

Antibodies used	anti-Nrx1-3 (Millipore, #ABN161-I), anti-Nrx1-3 (AB generation by LifeTein, #RB2987, rabbit, epitope: mouse NRX3A aa 1523-1571); anti-Nrx1-3 (Synaptic Systems, #175003), anti-Nrx1-3 (AB generation by AbFrontier, #3-4-rb1, rabbit, epitope: mouse NRX3A aa 1471-1491), anti-Nrx1 (Synaptic Systems, #175103), anti-Nrx1 (RnD Systems, #AF4524), anti-Nrx2 (AB generation by LifeTein, #RB4227, rabbit, epitope: mouse NRX2A aa 266-282), anti-Nrx2 (AB generation by LifeTein, #RB4226, rabbit, epitope: mouse NRX2A aa 1459-1476), anti-Nrx2 (AB generation by LifeTein, rabbit, #RB4225, epitope: mouse NRX2A aa 1459-1476), anti-Nrx2 (Abcam, #ab34245), anti-Nrx3 (AB generation by AbFrontier, rabbit, #3-1-rb1, epitope: mouse NRX3A aa 1349-1368), anti-Nrx3 (Synaptic Systems, #175303), anti-Nrx3 (AB generation by AbFrontier, rabbit, #3-2-rb1, epitope: mouse NRX3A aa 1377-1397), anti-Nrx3 (RnD Systems, #AF5269), anti-Nrx3 (AB generation by AbFrontier, rabbit, #3-2-rb2, epitope: mouse NRX3A aa 1377-1397), anti-T178B (AB generation by LifeTein, rabbit, #RB4235, epitope: mouse T178B aa 273-294), anti-T178B (AB generation by AmsBio, rabbit, #RB3967, epitope: mouse T178B aa 273-294), anti-HA (Roche, #11867423001), anti-HA (Thermo Fisher, #26183), anti-PTPRS (RnD Systems, #AF3430), anti-PTPRS (MediMabs, #MM-0020-P), anti-PTPRS (Invitrogen, #PA5-47487), anti-PTPR (Neuromab, #75-194), anti-CAH10 (RnD Systems, #MAB2189), anti-CAH10 (raised against recombinant CAH10 (71, rabbit, #Hadlai), anti-CAH10 (raised against recombinant CAH10 71, rabbit, #Habeba), anti-CAC1A (FrontierInstitute, #GP-Af810), anti-CAC1E (Alomone, #ACC-006), anti-TAFA1 (RnD Systems, #AF5154), anti-TAFA2 (RnD Systems, #AF4179), anti-NXPH1 (RnD Systems, #AF4208), anti-NXPH3 (RnD Systems, #AF5098), anti-LRRT4 (Neuromab, #75-261), anti-LRRT4 (AbFrontier, rabbit, #C1, raised against mouse LRRT4 aa 548-568), anti-LRRT4 (RnD Systems, #AF5377), anti-A4 (Abgent, #ABIN1741750), anti-A4 (Sigma, #A8717), anti-GBRA1 (Synaptic Systems, #224203), anti-GBRG2 (Synaptic Systems, #224003), anti-RTN4R (RnD Systems, #AF1440), anti-LRRT1 (MerckMillipore, #ABN642), anti-LRRT2 (NeuroMab, #75-364), anti-GRM4 (Abcam, #ab53088), anti-GRM4 (Abcam, #ab184302), anti-GRM4 (Bioworld, #BS1144), anti-NLGN2 (Synaptic Systems, #129511), anti-NLGN3 (NeuroMab, #75-158), anti-Syt11 (Synaptic Systems, #270003), anti-vGLU1 (Frontier Institute), anti-VIAAT (Frontier Institute), anti-V5 (BioRad, #MCA1360), anti-XKR6 (AB generation by AmsBio, rabbit, #RB3961, epitope: mouse XKR6 aa 85-102), anti-XKR6 (AB generation by AmsBio, rabbit, #RB3962, epitope: mouse XKR6 aa 85-102), anti-XKR6 (Signalway Antibody, #42864), anti-pan Neurexin (rabbit, Nittobo Medical Co., Ltd, # MSFR104640), anti-PSD95 (guinea pig, Nittobo Medical Co., Ltd, MSFR105190), IgG (Millipore, #12-370), rabbit pre-immune-sera (LifeTein).
Validation	All antibodies used for AP-MS experiments were validated by target-specificity and efficiency by quantitative mass spectrometry (detailed in Supplementary Figure 3, Results and Methods). Antibodies used for stainings were verfied in heterologous expressions by western blot and immunocytochemistry.

Eukaryotic cell lines

Policy information about cell lines and Sex and Gender in Research

Cell line source(s)	tsa201, Sigma, Cat#96121229)

Authentication	Cell lines were not authenticated.
Mycoplasma contamination	Cells were not contaminated.
Commonly misidentified lines (See ICLAC register)	Commonly misidentified lines were not used in this study.

Animals and other research organisms

Policy information about studies involving animals; ARRIVE guidelines recommended for reporting animal research, and Sex and Gender in Research

Laboratory animals	Wistar rat (adult and embryonic day E18), C57BL/6 mice: WT, NRX1A HA knock-in, NRX3A knock-out, NRX3 AS5 HA/HA knock-in, HA-Nlgn1 knock-in, HA-Cbln1, HA-Cbln2, T178B HA knock-in, CAH10 knock-out, 7-12 weeks; C57BL/6 mice: TMEM178A/B floxed, 3 weeks and P1
Wild animals	No wild animals were used in this study.
Reporting on sex	Sex was not considered. Animals of both sexes were used
Field-collected samples	This study did not use samples from the field.
Ethics oversight	Ethics committee: Regierungspräsidium Freiburg (Germany)

Note that full information on the approval of the study protocol must also be provided in the manuscript.

Plants

Seed stocks	*Report on the source of all seed stocks or other plant material used. If applicable, state the seed stock centre and catalogue number. If plant specimens were collected from the field, describe the collection location, date and sampling procedures.*
Novel plant genotypes	*Describe the methods by which all novel plant genotypes were produced. This includes those generated by transgenic approaches, gene editing, chemical/radiation-based mutagenesis and hybridization. For transgenic lines, describe the transformation method, the number of independent lines analyzed and the generation upon which experiments were performed. For gene-edited lines, describe the editor used, the endogenous sequence targeted for editing, the targeting guide RNA sequence (if applicable) and how the editor was applied.*
Authentication	*Describe any authentication procedures for each seed stock used or novel genotype generated. Describe any experiments used to assess the effect of a mutation and, where applicable, how potential secondary effects (e.g. second site T-DNA insertions, mosiacism, off-target gene editing) were examined.*
