# Supplementary material for: Ternary Neurexin-T178-PTPR complexes represent a pre-synaptic core-module of neuronal synapse organization
Source: Nat Commun. 2026 Sep 5;17:9509. doi: 10.1038/s41467-026-77377-4 (PMC13546301; doi:10.1038/s41467-026-77377-4)
Supplement: Supplementary file 3 [file 41467_2026_77377_MOESM3_ESM.pdf]

nature portfolio

Peer Review File

Ternary Neurexin-T178-PTPR complexes represent a pre-synaptic core-module of neuronal synapse organization

Corresponding Author: Professor Bernd Fakler

This file contains all reviewer reports in order by version, followed by all author rebuttals in order by version.

Version 0:

Reviewer comments:

Reviewer #1

(Remarks to the Author)
The overarching goal of this study was to systematically determine the neurexin proteome and thereby identify the molecular networks that neurexins coordinate in the mammalian brain. Thivaios et al. employ multi-epitope affinity purification combined with quantitative mass spectrometry to construct a comprehensive map of neurexin-associated proteins. This approach confirms well-established neurexin ligands, including neuroligins, LRRTMs, and cerebellins, while also expanding the interactome to encompass additional classes of synaptic organizers, adhesion molecules, and receptor complexes. The authors argue that these datasets reveal neurexins as central nodes in large presynaptic and trans-synaptic networks that extend beyond previously appreciated binary interactions. Within this broader context, they highlight the discovery of a ternary complex containing neurexins, LAR-type receptor protein tyrosine phosphatases, and the tetraspanins T178A/B. They suggest that this complex forms a presynaptic core-module that organizes active zones and aligns presynaptic release machinery with postsynaptic receptors. To support these claims, the manuscript integrates proteomic analysis with native gels, AlphaFold-based structural predictions, immuno-electron microscopy, and electrophysiological assays. While the dataset is large and technically impressive, the claims about the essentiality and universality of the ternary complex, particularly the role of T178, are exaggerated and not convincingly demonstrated.

Strengths
The most significant strength of the manuscript lies in its scale and ambition. The authors provide one of the most comprehensive maps of the neurexin interactome to date, greatly extending prior synaptic proteomic. This resource has intrinsic value to the field, independent of any single mechanistic claim, because it confirms known interactions and uncovers new candidate partners that can be followed up in future studies. The breadth of associations uncovered including known synaptic organizers, receptor complexes, and scaffolding proteins underscores the idea of neurexins as multi-faceted hubs for presynaptic assembly and trans-synaptic signaling.
A second strength is the integrative experimental strategy. The authors do not rely solely on proteomics but attempt to connect their biochemical findings with cellular localization and physiological function. The immuno-EM analysis provides spatial resolution of neurexin distribution at active zones, and the electrophysiological assays attempt to link molecular assembly to presynaptic function. Together, these diverse approaches demonstrate the authors' technical sophistication and their commitment to situating proteomic observations in a functional context.

Weaknesses
Despite these strengths, the manuscript has major weaknesses that undermine its conclusions. The most problematic aspect is the aspects of T178. The authors build their central mechanistic story around the claim that T178A/B act as essential "brackets" stabilizing NRX-PTPR complexes. Yet all of their functional evidence relies on shRNA-mediated knockdown rather than genetic knockout. Knockdown approaches are inherently limited by incomplete penetrance, residual protein, and potential off-target effects. Indeed, the authors' own proteomic data show that neurexins and PTPRs remain detectable after T178B knockdown, suggesting that the assemblies persist, albeit in altered form. This makes it impossible to conclude that T178 is required. Only knockout models could establish necessity, and in their absence the story remains speculative.
The physiological experiments do little to strengthen the case. The observed changes in paired-pulse ratio are modest and nonspecific. Since basal synaptic transmission remains intact, these data argue against T178 as a core organizer of presynaptic function. The heterologous expression assays further complicate the picture by showing that NRX-PTPR interactions persist, albeit weakly, without T178, which suggests a modulatory rather than essential role. The claim that T178

is indispensable is thus overstated.
A second weakness is the framing of the ternary assembly as a “universal presynaptic core-module.” This description is not supported. Synapses remain functional after T178 knockdown, and decades of genetic studies, including NRX triple knockouts and LAR-PTPR deletions, have consistently shown that synapse formation and function are remarkably resilient. Redundancy and compensation are built into the system, and this manuscript does not adequately grapple with that reality.
Finally, the scope of the physiology and structural analysis is too narrow to support the sweeping claims. The AlphaFold-based docking predictions are intriguing but speculative and untested. Moreover, the discussion underplays extensive prior literature on neurexin in order to present the ternary module as a paradigm shift, which it is not.
This work will only be suitable for publication after substantial new data and conceptual reframing. Most critically, the role of T178 must be established using genetic knockout rather than shRNA knockdown. Without KO models, the claim that T178 is required for the NRX-PTPR complex cannot stand, and the manuscript should not be considered further. Functional analyses should be broadened to include direct measures of presynaptic release probability such as variance-mean analysis, vesicle pool labeling, or optical glutamate sensors rather than relying exclusively on paired-pulse ratio. The authors should also assess whether the NRX–T178–PTPR module functions similarly across different synapse types, given the well-established circuit-specific roles of neurexins. Structural predictions must be validated experimentally, and the discussion should be revised to place the findings within the context of redundancy and prior adhesion molecule studies, rather than overstating novelty.

Reviewer #2

(Remarks to the Author)
This manuscript presents a methodologically outstanding study that defines a ternary complex of Neurexins, LAR-type PTPRs, and the previously uncharacterized tetraspanins T178A/B as a stable biochemical unit in the mammalian brain. Using a combination of advanced proteomic techniques, including multi-epitope affinity purifications, complexome profiling of ER and plasma membrane fractions, and high-resolution quantitative mass spectrometry, the authors convincingly demonstrate that these proteins co-assemble into a ~400 kDa complex already during early biogenesis in the ER. Additional support is provided by reverse APs, native gels, heterologous expression, and immuno-EM, culminating in a dense interaction network centered around this tripartite complex.

The quality of the biochemical work is exceptionally high throughout. The systematic application of meAP-MS, the careful use of multiple antibodies and knockout controls, and the combination of native gels with complexome profiling (csBN-MS) are particularly impressive. The organellar proteomics and ER-based co-assembly data are among the strongest aspects of the study, clearly demonstrating that this complex is not only stable but likely trafficked as a preassembled unit. These datasets represent a major technical and conceptual contribution to the field.

The authors propose that this ternary complex forms a “core module” for synapse formation and argue that its identification marks a “paradigm shift” in our understanding of synapse-organizing factors. While the study makes an important structural and molecular contribution, this interpretive framing may go somewhat beyond what the functional data support. The designation as a “core module for synapse formation” suggests an essential role in assembly, yet knockdown of T178B leads to relatively moderate changes in paired-pulse ratios and redistribution of Neurexins, without impairing synapse formation or causing overt transmission failure. Thus, although the ternary complex likely plays a widespread and conserved role in presynaptic architecture, its indispensability is not firmly established. A more restrained interpretation—as a scaffolding platform that coordinates trans-synaptic linkage and stabilizes molecular assemblies—might better reflect the data.

Along similar lines, the paper frames the coordination of multiple sCAM families as a major unresolved question and implies that the findings provide a new conceptual model. However, extensive prior work has shown that sCAMs are expressed in overlapping, combinatorial patterns and contribute to synapse-type diversity. The current study adds important structural and proteomic resolution to these observations but does not necessarily shift the conceptual ground. In this sense, the novelty is more technical and molecular than paradigmatic.

One weaker aspect of the manuscript is the physiological readout. The increase in paired-pulse ratio upon presynaptic T178B knockdown is consistent with a presynaptic effect but remains difficult to interpret mechanistically. Without additional functional assays, it is hard to connect the observed biochemical reorganization to specific changes in neurotransmitter release, vesicle dynamics, or synapse function. This limits the physiological insight and makes the functional consequences of complex disruption somewhat abstract.

Another point that could be reconsidered is the use of AlphaFold to propose a membrane-proximal structure of the complex. While such modeling is increasingly common, in the absence of experimental validation (e.g., crosslinking-MS, mutagenesis), the AlphaFold-based structural predictions remain speculative. Given the strength of the biochemical data, the model adds relatively little and might be better relegated to a supplementary figure with appropriate disclaimers.

Finally, some statements could be clarified for precision. For example, the phrase “strong preference for (localization to) active zones” in the EM section should be more explicit: Neurexins are enriched at active zones relative to extra-synaptic membrane regions. These details are important to convey the findings accurately and avoid ambiguity.

Conclusion:
This is a high-quality and rigorous study that provides compelling biochemical evidence for a widely distributed presynaptic

protein assembly involving Neurexins, LAR-PTPRs, and tetraspanins. The experimental design and execution are exemplary, and the dataset will be a valuable resource for the field. While some of the broader interpretive claims could be toned down, the overall contribution is clear and important. I support publication of this work after minor revisions aimed at sharpening the conceptual framing and clarifying a few overstated points.

Reviewer #3

(Remarks to the Author)
This manuscript uses an unbiased quantitative analysis of native protein assemblies at synapses by multi-epitope affinity-purifications (meAPs). Using this approach they propose a new pre-synaptic core-module consisting of NRXs, LAR-PTPRs, and previously uncharacterized tetraspanins T178A/B. The manuscript provides evidence for the assembly of the ternary structure of NRX-T178-PTPR during biogenesis in the ER, highlights that T178 functions as the structural 'bracket' stabilizing the interaction between NRXs and LAR-PTPRs, and regulates the distribution of NRXs within presynaptic active zones. The authors further discuss the integration of the core module into synaptic protein networks by performing a series of reverse meAPs targeting an array of NRX-interactome constituents. Overall, this manuscript presents a conceptually significant idea that defines a novel presynaptic core module bridging two well-characterized adhesion systems in synapse organization.

Major concerns:
1) AlphaFold docking & structural inference: The paper uses BN-PAGE, reverse-APs and AlphaFold predictions to infer the ternary structure, which provides valuable yet insufficient validation of the direct NRX-T178-PTPR contact. Adding crosslinking-MS experiments the meAP of this complex would address the following:
a) Direct contact evidence to support the proposed 1:1:1 ternary structure and the AlphaFold docking.
b) The BN-PAGE result is consistent with the 1:1:1 stoichiometry, yet it's indirect in the fact that glycosylation could skew the results, so crosslinking MS would help solidify the results.
c) Prove T178-dependent interfaces for its function as the structural 'bracket'. Comparisons between T178B-present and T178B-absent states to prove T178-dependent stabilization of NRX-PTPR interaction.

2) T178A redundancy: The paper only interrogates the function of T178B through T178B knockdown, yet the role of T178A is unexplored. Does the expression level of T178A change in response to T178B knockdown? Is there any potential T178A redundancy that masks some stronger phenotypes? Dual perturbation of T178A and T178B could be a good way to address this possibility. Alternatively, if these experiments are not currently feasible, the authors should at least acknowledge the possibility that T178A could have certain functions and may mask some phenotypes. Additionally, the authors should discuss the expression level of T178A and T178B in a cell-type and brain-region specific manner based on existing datasets in the literature.

3) The paper does not dissect excitatory and inhibitory synapses when showing NRXs localization in the T178B knockdown condition in Figure 4E. This was done for the WT condition, but curiously missing from the knockdown data. The authors should also do analyses on excitatory versus inhibitory synapses in the T178B knockdown condition. Could the consequences of T178B differ between the synapse types?

4) There are several concerns need to be addressed in Figure 3A:
a) The data shown here seems to disagree with the data of ER-specific membrane analysis shown in Figure 3C, since none of NRXs, T178 or PTPRs appear in the ER cluster.
b) The authors should clarify the significance of Figure 3A in the general context. If it aims at showing the clustering of the ternary structure components, it makes more sense to perform covariation analysis of protein levels instead of organellar clustering. See the following literature for analysis of this type of data using clustering algorithms (PMCID: PMC6338729; PMC7984842)

5) In Figure 3C, the authors should show that the ER membrane-enriched fraction is indeed ER-membrane enriched by using ER-specific markers (either MS or immunoblot).

6) The authors should tone down and not use the word 'universal' to describe the complex given that they don't show that the complex is universal at various types of synapses such as dopaminergic, serotonergic synapses, etc., or across even brain regions.

Minor concerns:
7) Please add replicate-level statistical significance analysis into the figures throughout the paper.
8) The authors should give more introduction to the tetraspanins before hypothesizing T178A/B to be part of the ternary complex.
9) The paper claims that 'these analyses showed that the major portion of NRXs is covalently linked to CAH10' in Figure 1D. Please remove "covalently" or explain how the data supports a covalent interaction is shown, given that AP experiments don't necessarily indicate covalent binding.
10) Please provide the AlphaFold3 docking scores (pTM and ipTM) in Figure 2F. If these are low (pTM below 0.5 or ipTM below 0.6), then the crosslinking MS data may help by incorporating the data into AlphaLink using ColabFold: https://github.com/Rappsilber-Laboratory/AlphaLink2?tab=readme-ov-file
11) Please edit for consistent hyphen use in terminology in figures and text. For example, presynaptic versus pre-synaptic.
12) Page 4 typo: 'fast majority' should be 'vast majority'.

Version 1:

Reviewer comments:

Reviewer #1

(Remarks to the Author)

The authors have improved the manuscript somewhat; however, major conceptual and technical issues persist that cast significant doubt on their central claim regarding the purported Neurexin T178 PTPR core module.

As is evident across all figures involving T178 knockdown (for example, Figure 3D), PTPR levels are dramatically reduced following T178 depletion, and the lysates clearly show a marked loss of PTPRs. In this context, the Neurexin1-3 affinity purifications still retrieve PTPRs in proportion to its abundance in the T178 knockdown lysates, indicating that the apparent loss of Neurexin-bound PTPR largely reflects reduced PTPR levels rather than a specific disruption of a Neurexin-PTPR interaction. Furthermore, in the co-expression experiments (Figure 2E and all panels of supplementary Figure 6A), the PTPR subunit appears unstable and cleaved in the absence of T178, consistent with previously described γ-secretase-dependent cleavage or cleavage by Adam10 or 17 of related PTPR family members (see Haapasalo et al., 2007, JBC,). Most concerningly, in their earlier biorXiv manuscript posted on July 19, 2024, the authors themselves reported that T178 knockdown in primary neurons reduced total PTPRS levels by approximately 78% and surface PTPRS levels by nearly 90%. This critical dataset, which speaks directly to how lack of T178 affects PTPR stability, has been omitted from both the current submission and the subsequent biorxiv version submitted on April 10, 2025.

Taken together, the evidence strongly suggests that the primary role of T178 is to stabilize PTPRs and promote its surface expression, with any effects on protein-protein interactions such as with Neurexins being secondary. Indeed, the authors' own data (including their new Figure 2F) better support this interpretation than the speculative ternary complex model they continue to promote. The notion of a direct Neurexin T178 PTPR "core module" is therefore unsubstantiated and misleading; what the data actually reveal is a indirect or secondary effect driven by PTPR stability, a valid and interesting mechanism in its own right, but not the one claimed. Therefore, a reinterpretation of the results is warranted.

The reported approximately 50% mislocalization of Neurexin under T178 knockdown is also problematic (Figure 4). Given the well-known challenges of reliable Neurexin immunostaining, immuno-EM quantification without knockout or knockdown validation lacks rigor and should not be considered definitive evidence. Moreover, with that level of neurexin mislocalization, one would expect major deficits in basal synaptic function, which was absent in the author's own data, as well as in another work from Thomas Sudhof's lab (https://purl.stanford.edu/cp200pt0640). I would either remove this data or improve upon it with KO/KD control experiment for neurexin antibody validation in immuno-EM. The same can be said for Supplementary Figure 8.

Finally, the manuscript fails to acknowledge extensive prior work demonstrating that the Neurexin-PTPR interaction is largely dependent on heparan sulfate modification of Neurexin (see for example, Han et al, J Neurosci, 2020 Oct 28;40(44):8438-8462 and Roppongi et al. Neuron, 2020 Apr 8;106(1):108-125.e12), a major omission that distorts the interpretive context. In Figure 2D, the authors assess heparinase effects on protein abundance by mass spectrometry, yet the assay buffer employed may contain detergents inhibitory to enzymatic activity. These experiments must be repeated under either detergent-free or heparinase-compatible conditions reported in previous reports, and validated by quantitative immunoblotting.

Likewise, the structural model in Figure 2G is not convincingly supported. Although the figure legend claims it is based on interaction assays (C–F), the model bears little resemblance to those data. Moreover, both UniProt and AlphaFold offer conflicting predictions for T178B topology (three versus four transmembrane domains, respectively), and the low confidence scores reported (pTM = 0.52; ipTM = 0.26) are well below accepted thresholds for a reliable multimeric model according to EMBL–EBI AlphaFold guidelines. ipTM values below 0.8 are considered failed predictions (https://www.ebi.ac.uk/training/online/courses/alphafold/inputs-and-outputs/evaluating-alphafolds-predicted-structures-using-confidence-scores/confidence-scores-in-alphafold-multimer/). Mutant-based domain mapping would be a considerably more convincing approach.

In its current form, the manuscript overinterprets its results- one cannot draw conclusions of a protein interactions when the protein itself is unstable, omits critical contradictory evidence, and fails to adequately consider prior literature. The conclusions as stated are neither rigorously supported nor mechanistically credible.

Other major concerns

1. It is erroneous to claim that the so-called Neurexin–T178–PTPR module "recruits stable and extended trans-synaptic protein networks with defined pre- and post-synaptic partners and secreted extracellular linkers." The authors provide no compelling evidence to support such an expansive statement other than co-isolation in mass spectrometry. The only other data presented are modest reductions in protein abundance detected by mass spectrometry, which cannot by itself establish recruitment, stabilization, or structural assembly of any trans-synaptic network. There is no demonstration that synaptic networks are altered, destabilized, or "collapsed" in the absence of T178, nor is there biochemical, imaging, or functional

evidence of complex assembly. This sweeping mechanistic claim is therefore unsupported by rigour and represents a serious overreach beyond the data shown. At best, the results suggest a change in steady-state protein levels, not the recruitment or maintenance of a defined trans-synaptic architecture.

2. The authors state that “all three NRXs co-assemble with T178B and LAR-PTPRs into previously unrecognized stoichiometric complexes,” yet this claim is not unique to PTPR-Neurexin interaction based on their own data. In Figure 1D and 1E, several additional proteins show comparably robust co-assembly with T178B and LAR-PTPRs, indicating that formation of such stoichiometric complexes is not specific to Neurexins and therefore cannot be taken as definitive evidence for a privileged Neurexin-T178-LAR-PTPR module.

3. On page 6, the authors state: “In addition, these analyses showed that the major portion of NRXs is covalently linked to CAH10 and interacts with the secreted CBLN1 protein.” However, if most neurexins are bound to CA10 and thereby lack HS, it remains unclear why endogenous neurexins still run as broad smear bands, which typically indicate heterogeneous HS modification. In addition, if CA10-bound, HS-deficient neurexins are indeed the predominant species, it is difficult to reconcile this with the strong synaptic and behavioral phenotypes reported in Nrxn1ΔHS mice, where only the HS attachment site on Nrxn1 is mutated (see Zhang et al, 2018, Cell). Please clarify how your model accounts for the apparent abundance of HS-modified neurexin species on SDS-PAGE and explain why disrupting HS at a single neurexin locus (Nrxn1ΔHS) produces such pronounced phenotypes despite the proposed dominance of CA10-bound, non-HS neurexin complexes.

Minor concerns

1. The claim that PTPRD was identified as a new partner of neurexin is not correct. It was already shown before, see Han et al, J Neurosci, 2020 Oct 28;40(44):8438-8462. Please remove this claim.

2. Another concern is how the authors ensured that the analyzed immune-EM synapses actually originated from T178 knockdown neurons, given that no infection reporter is visible in the EM images. In Fig. 4E, it is also unclear why the WT condition combines excitatory and inhibitory synapses, whereas the T178 KD condition is shown as “all synapses” and “excitatory synapses” separately; the rationale for this mismatch in sampling and presentation should be explained. Since T178 KO or tagged mice are available, the authors should instead (or additionally) validate and directly confirm synaptic localization of T178 in situ.

3. I recommend that the authors include a table summarizing all antibodies used for AP, WB, and IHC, indicating for each antibody the target, host species, source/catalog number, dilution, and the specific experiments/figures in which it was used, to facilitate interpretation and enhance reproducibility.

Bottomline and recommendation
My main concern is that, in light of prior reports demonstrating heparan sulfate–dependent neurexin–PTPR interactions, together with the evidence in this study that T178 primarily affects PTPR levels (and surface expression in the original bioRxiv version), as well as data from the Südhof lab showing minimal synaptic phenotypes upon removal of T178A/B, the current conclusions proposing a functionally significant ternary complex are not adequately supported by the available evidence and appear substantially overstated.

As currently framed, the strong emphasis on a ternary complex risk giving a misleading impression to the field and could propagate an incorrect conceptual framework. I therefore recommend that the claims about T178 as the mediator of ternary complex formation be thoroughly reconsidered, instead the emphasis should be on how T178 is regulating stability of PTPRs and its lack thereof predictably affects its complexes as a secondary outcome. This should be emphasised throughout the manuscript, including in the Title, Abstract and Discussion. Given that the authors are now developing tagged mouse lines and additional tools, definitive statements about the existence and mode of interaction of a ternary complex should, in my view, be deferred to future studies employing rigorous structural biology and functional analyses. Provided that the authors substantially revise, moderate, and clearly qualify their claims about the ternary complex along these lines, I would be supportive of publication, as the proteomics data appear to be of high quality and will constitute a valuable resource for the field.

Reviewer #2

(Remarks to the Author)
From my perspective, the authors have responded thoughtfully and constructively to the reviewer’s comments, appropriately refining their claims and improving the clarity of the manuscript. In its revised form, I am pleased to support acceptance.

Reviewer #3

(Remarks to the Author)
We thank the authors for carefully addressing our comments and revising the manuscript. The manuscript has improved substantially and all of our concerns have been addressed, with just one remaining textual change request.

Concerns that were adequately addressed
Structural validation (original concern #1): We appreciate the authors’ attempt to perform crosslinking-MS. While it did not

yield crosslinked peptides, this is understandable. Importantly, the authors added new pairwise binding data from heterologous expression and quantitative MS measurements demonstrating that T178 increases the stability of the NRX1A–PTPRS interaction by approximately 10-fold. These data corroborate the role of T178 as a structural bracket that stabilizes the ternary complex. Relocating the AlphaFold model to the supplement is appropriate given that it remains unvalidated and other reviewer's suggested this as well.

T178A redundancy (original concern #2): The authors added functional recordings on dentate gyrus granule cells using a T178A,B double conditional knockout mouse, as suggested. This is a substantial experimental addition that goes beyond what we originally requested. The double KO in entorhinal cortex produces presynaptic phenotypes (altered paired-pulse ratios, increased release probability) consistent with the T178B knockdown data, which largely addresses the concern of T178A redundancy and also successfully addressed Reviewer 1's objection about reliance on shRNA (in my opinion).

Subcellular fractionation and other concerns (#4–6, #7–12): We appreciate that the authors added the distribution profiles of the core-module constituents together with averages of PM and ER markers across subcellular fractions (new Suppl. Fig. 7), showing that the core-module constituents have high relative abundance at the same fractions. The replicate-level statistics, textual revisions (removal of "universal," toned-down framing), and all other minor concerns have been properly addressed in the revised manuscript.

One remaining minor issue
Excitatory vs. inhibitory synapse analysis (original concern #3): The authors attempted to analyze the distribution of NRXs after T178B knockdown at inhibitory synapses, as requested, but did not obtain distribution data of comparable quality to excitatory synapses. We accept this technical limitation. However, the revised manuscript does not explicitly acknowledge this gap. Given the well-established circuit- and synapse-type-specific roles of neurexins, the authors should mention their attempt to address this question in the manuscript text and acknowledge T178B's contribution to NRX localization and presynaptic function may differ at inhibitory versus excitatory synapses. A brief statement in the Results section (around Figure 4E) and/or in the Discussion would suffice.

In summary, the revised manuscript has been significantly improved and represents a strong contribution to the field. The addition of the double conditional knockout mouse model was a substaintial and well done addition and it greatly strengthens the conclusions.

Version 2:

Reviewer comments:

Reviewer #1

(Remarks to the Author)
Despite the additional work and clearer presentation in the revised manuscript, I remain unconvinced that the new data resolve the main interpretive problems raised in my initial and follow-up review. The central conceptual issue remains unresolved: in my view, the data are fully compatible with T178 acting primarily to stabilize PTPR expression and surface delivery, with any changes in Neurexin-PTPR association arising as secondary consequences of altered PTPR availability, whereas the manuscript still presents a privileged ternary Neurexin-T178-PTPR "core module" as an established mechanistic entity, thereby overstating what can be rigorously inferred from the experiments. I outline below where I see persistent gaps between data and claims, and how a more conservative, quantitatively grounded re framing could better align the conclusions with the available evidence.

1. T178, PTPR stability, and "over proportional" loss
The authors argue that T178 removal reduces total PTPRs by only 30-40% (MS) but causes a "profound over proportional" reduction of PTPRs in Neurexin APs, which they interpret as evidence that T178 directly stabilizes Neurexin-PTPR binding ("bracketing"). There are several problems here:
a. The rebuttal does not provide the underlying quantitative metrics (effect sizes, variance, normalization strategy) for Supplementary Fig. S9D, only the qualitative assertion of "over proportionality." Without explicit stoichiometric comparisons (e.g. co purified PTPR normalized to input PTPR and Neurexin) and proper statistical treatment, this central claim remains descriptive rather than mechanistic.
b. The authors now acknowledge that earlier primary neuron data showed ~78% reduction in total PTPRS and ~90% reduction at the surface upon T178 knockdown, which they omitted because of "limited dynamic range." Yet this dataset is conceptually crucial: it makes the simplest model one in which T178 primarily governs PTPR stability/surface trafficking and, only as a consequence, the amount of PTPR available to participate in Neurexin complexes. Because the proteomic approach ultimately quantifies total protein abundance, even when applied to membrane-enriched fractions, it cannot, on its own, provide a foolproof measure of surface levels. By contrast, surface immunostaining remains a widely accepted standard for assessing cell-surface expression, and the authors' decision to dismiss their own staining data as merely "limited in dynamic range," without quantitative justification or comparison, reads more as an opinion than as a substantiated methodological critique.
c. The statement that reduced co purification "far exceeds" the drop in total PTPR does not by itself prove altered binding or support a ternary core module. It could also reflect changes in subcellular localization, membrane microdomain access, or reduced avidity due to lower local PTPR density.

In other words, the new data are compatible with T178 exerting a strong upstream effect on PTPR abundance and localization, which predictably impacts Neurexin bound PTPR pools, but they do not force the specific mechanistic conclusion that T178 "brackets" Neurexins and PTPRs into a defined ternary structural module. A more cautious interpretation that foregrounds PTPR stability and surface expression as the primary function of T178, with consequences for Neurexin PTPR complexes treated as downstream, would be better aligned with the totality of the evidence, including the authors' own earlier bioRxiv dataset.

2. Claims of a privileged ternary "core module"
The authors continue to state that "all three NRXs co assemble with T178B and LAR PTPRs into previously unrecognized stoichiometric complexes" that serve as "core modules of stable trans synaptic protein networks," and argue that only Neurexins, LAR PTPRs, and T178 are "effectively and consistently retrieved" across multiple synaptic APs. Several issues remain:
a. By their own t SNE and AP datasets (Fig. 1D,E; Fig. 5; Fig. 6), multiple additional proteins show robust and recurrent co assembly with T178B and LAR PTPRs. The rebuttal tries to elevate the Neurexin-T178-PTPR triad as uniquely stoichiometric, but without explicitly quantifying stoichiometry relative to other partners, this remains an interpretive preference rather than a demonstrated property.
b. The authors acknowledge that their proteomics primarily report "steady state" co isolation rather than recruitment dynamics. Yet the text still uses strong language such as "recruits stable and extended trans synaptic protein networks" and "core modules". The proteomics data only reveal which proteins co-isolate with T178 at steady state, but they do not show how these proteins are recruited or assembled. Therefore, language such as "recruits stable trans-synaptic networks" or "core modules" may be too strong unless supported by independent evidence of assembly, structural organization, or functional dependence in vivo.
c. There is still no direct demonstration that the proposed networks are destabilized upon T178 loss in a way that cannot be fully explained by changes in PTPR abundance. The observed changes in MS abundance and patch clamp measures support an important role for T178, but do not alone define a specific ternary architecture.

The authors should make it clear that the "core module" idea remains an interpretation or working model rather than a proven fact. Their experiments provide several solid findings, including robust protein co-purification, strong dependence of PTPR levels on T178, and modest changes in ppr when T178 is lost. However, these findings do not automatically prove the existence of a specific physical "core module" or ternary complex. That conclusion remains a proposed explanation for the data. The concern is that the current text mixes up the distinction between what the experiments directly show, namely that T178 affects PTPR levels, protein co-purification, and synaptic function, and what the authors infer from those findings, namely that T178 forms or organizes a defined core module or ternary architecture.

3. Neurexin mislocalization and functional consequences
The immuno EM data suggesting ~50% mislocalization of Neurexin in the absence of T178 remain a weak link in the proposed mechanism. Although the authors emphasize MS based validation of their custom Neurexin antibody and similarity to prior EM staining, this is insufficient, because antibody performance is application specific and the crucial control, KO/KD based validation of the EM labeling, is still missing, which is particularly problematic given the historical difficulty of Neurexin immunostaining and the magnitude of the reported effect. Moreover, if such a large redistribution of Neurexin is invoked as a central mechanistic pillar, the relatively modest basal synaptic changes and the observation that ~50% of Neurexin exits active zones need to be explicitly reconciled with this claim rather than used to dismiss concerns about structure-function correspondence. At minimum, the EM based redistribution should be presented as suggestive rather than definitive, with its limitations (lack of genetic validation in the EM context and incomplete infection coverage) clearly acknowledged, or else the manuscript should tone down mechanistically strong statements that rely heavily on these EM results.
4. Heparan sulfate dependence and prior literature
The discussion of prior work on HS-dependent Neurexin-PTPR interactions remains insufficient and contributes to the impression that the current manuscript is overlooking, rather than integrating, important existing evidence.
a. The rebuttal emphasizes that Han et al. and Roppongi et al. used "qualitative Western blotting" and questions their quantitative rigor, but does not sufficiently acknowledge that multiple independent groups have converged on an HS dependent Neurexin-LAR PTPR interaction using different approaches.
b. The authors clarify that their heparinase treatment was performed on native tissue in detergent free conditions, and that they see strong loss of LRRTs and AMPARs but preserved co purification of PTPRs and T178 with Neurexins. This is valuable, but it establishes that a subset of Neurexin-PTPR complexes persists without HS, not that HS is irrelevant for Neurexin-PTPR biology more broadly.
c. The GPI-anchored, HS-modified Neurexin3 construct, which fails to bind T178 and PTPRs, is interpreted as showing that HS is neither required nor sufficient for complex formation; however, the authors do not demonstrate that this GPI-anchored Neurexin3 is heparan-sulfated to a comparable extent as the full-length protein, so the negative result cannot be taken as a clean test of HS sufficiency.

As written, the current manuscript leans toward "correcting" the literature without sufficient experimental head to head comparison.

5. Structural model and topology of T178B
The authors now explicitly describe Fig. 2G as a "working model". This is a welcome clarification but does not fundamentally resolve the concerns:
a. The AlphaFold Multimer ipTM and pTM scores remain in a range that EMBL–EBI classifies as unreliable for complex topology, and this is not counterbalanced by any mutational domain mapping or crosslinking data that would independently

validate the proposed arrangement.
b. The model is still described as summarizing “extensive series of pairwise binding studies,” yet the binding assays themselves do not uniquely dictate the geometry or stoichiometry of the transmembrane interactions; they only indicate that T178 can promote Neurexin-PTPR co purification under the tested conditions.
The figure and text should make it unequivocally clear that the structural depiction is a speculative synthesis built on low confidence in silico predictions plus biochemical interaction data, not a structural determination, and that alternative arrangements (including models in which T178 primarily modulates PTPR stability and microenvironment rather than acting as a rigid “bracket”) remain plausible.

6. Scope of claims and recommended re framing
In its current form, the manuscript still contains multiple statements that imply a level of mechanistic certainty about a ternary Neurexin-T178-PTPR “core module” that is not warranted by the data. This includes language in the Title, Abstract, and Discussion about:
- “recruitment” of extended trans synaptic networks;
- “core modules” and “stoichiometric complexes” that are privileged relative to other Neurexin assemblies;
- T178 “interlinking” Neurexins and PTPRs as the central mechanistic role, rather than primarily stabilizing PTPRs at the cell surface thereby facilitating their broader interaction landscape, including interactions with Neurexins, rather than directly forming a specific interlinking module.
Given the strength of the proteomic dataset and the clear importance of T178 in shaping PTPR biology, I would be enthusiastic about publication if the manuscript were substantially reframed along the following lines:
- Emphasize that T178 is a critical regulator of PTPR abundance, stability, and/or surface expression, with robust quantitative proteomic support and electrophysiological consequences.
- Present the Neurexin-T178-PTPR complex as one prominent configuration within a broader Neurexin-LAR PTPR landscape that includes known HS dependent interactions, rather than as a single defining core module.
- Explicitly label all structural and network level models as working hypotheses, clearly separated from experimentally established findings.
- Tone down or remove language that implies recruitment/assembly of defined trans synaptic architectures in the absence of direct structural, imaging based, or functional complex disruption evidence.
- Remove over-interpretations from EM data if including in manuscript and clearly state the caveats.
If the authors are willing to adjust, I would be supportive of publication. Without such re framing, I remain concerned that the current narrative risks cementing an overly rigid conceptual framework that the present data do not unambiguously justify.

Open Access This Peer Review File is licensed under a Creative Commons Attribution 4.0 International License, which permits use, sharing, adaptation, distribution and reproduction in any medium or format, as long as you give appropriate credit to the original author(s) and the source, provide a link to the Creative Commons license, and indicate if changes were made.
In cases where reviewers are anonymous, credit should be given to 'Anonymous Referee' and the source.
The images or other third party material in this Peer Review File are included in the article’s Creative Commons license, unless indicated otherwise in a credit line to the material. If material is not included in the article’s Creative Commons license and your intended use is not permitted by statutory regulation or exceeds the permitted use, you will need to obtain permission directly from the copyright holder.

To view a copy of this license, visit https://creativecommons.org/licenses/by/4.0/

Responses to the reviewers‘ comments

Reviewer 1

Strengths

The most significant strength of the manuscript lies in its scale and ambition. The authors provide one of the most comprehensive maps of the neurexin interactome to date, greatly extending prior synaptic proteomic. This resource has intrinsic value to the field, independent of any single mechanistic claim, because it confirms known interactions and uncovers new candidate partners that can be followed up in future studies. The breadth of associations uncovered including known synaptic organizers, receptor complexes, and scaffolding proteins underscores the idea of neurexins as multi-faceted hubs for presynaptic assembly and trans-synaptic signaling.

A second strength is the integrative experimental strategy. The authors do not rely solely on proteomics but attempt to connect their biochemical findings with cellular localization and physiological function. The immuno-EM analysis provides spatial resolution of neurexin distribution at active zones, and the electrophysiological assays attempt to link molecular assembly to presynaptic function. Together, these diverse approaches demonstrate the authors' technical sophistication and their commitment to situating proteomic observations in a functional context.

We appreciate the reviewer‘s statements highlighting scale, importance and sophistication of our work. Moreover, we are grateful that the reviewer acknowledges the ‘intrinsic value’ of the work as an integrated resource for the field.

Weaknesses

Despite these strengths, the manuscript has major weaknesses that undermine its conclusions.
The most problematic aspect is the aspects of T178. The authors build their central mechanistic story around the claim that T178A/B act as essential “brackets” stabilizing NRX-PTPR complexes.

Yet all of their functional evidence relies on shRNA-mediated knockdown rather than genetic knockout. Knockdown approaches are inherently limited by incomplete penetrance, residual protein, and potential off-target effects. Indeed, the authors' own proteomic data show that neurexins and PTPRs remain detectable after T178B knockdown, suggesting that the assemblies persist, albeit in altered form. This makes it impossible to conclude that T178 is required. Only knockout models could establish necessity, and in their absence the story remains speculative.

The physiological experiments do little to strengthen the case. The observed changes in paired-pulse ratio are modest and nonspecific. Since basal synaptic transmission remains intact, these data argue against T178 as a core organizer of presynaptic function. The heterologous expression assays further complicate the picture by showing that NRX-PTPR interactions persist, albeit weakly, without T178, which suggests a modulatory rather than essential role. The claim that T178 is indispensable is thus overstated.

General remarks

The major goal of this work was the comprehensive, unbiased and quantitative analysis of the architecture of the protein network(s) associated with the NRX proteins in the brain by *deep proteomics* (meAP-MS with 16 different anti-NRX ABs, followed by meAP-MS with 64 APs targeting a variety of network constituents, plus APs with 27 control ABs).

As an outcome, these analyses uncovered **extended and highly stable protein networks** that we found **centered around a previously unrecognized ternary element assembled from NRXs, LAR-PTPRs and the tetraspanins T178A,B**. This ternary element was characterized in its basic role for network assembly/integration (Figs. 1, 2, 4-6), and T178 was characterized in its basic function to interlink the two major families of sCAMs, NRX and LAR-PTPRs (Figs. 2, 3).

In their comments regarding the strengths of the manuscript, the reviewer acknowledges the importance of this major part of the work.

The weakness regarding methodology and interpretation for the T178 knockdown experiments noted by the reviewer relates to only a minor fraction of the work presented in the manuscript (Fig. 3D, E of the original manuscript). To address these comments, we conducted an extensive set of additional experiments and modified the manuscript accordingly. These are described below:

(1) ***Role of T178 for the stability of the ternary core-complexes***

The profound stabilization of the NRX1A-PTPRS interaction by T178B documented in the original ms by Western blotting (Fig. 2E), was complemented in the revised ms by quantitative MS-measurements demonstrating that T178 increases the stability of the NRX1A-PTPRS interaction by as much as a **factor of 10** (**new Figure 2F**).
The sole NRX-PTPRS interaction is weak (or not even occurring for some NRX isoforms). Please note that in the original ms no claim was made of 'T178 being indispensable' as indicated in the reviewer's comment.

In addition, the revised ms

- presents this profound interlinking effect of T178B also for the interaction between NRX1G and PTPRS (**new Figure 2E**, right panel), and
- highlights robust binding of T178 to both NRX1A/G and NRX3 in pairwise expressions (**new Figure 2E**, left panel, **new Suppl. Fig. 6A**).

Finally (contrary to the reviewer's statements), the stabilizing function of T178 was perfectly recapitulated in neurons (neuronal cultures and brain), where essentially the entirety of T178 co-assembles with NRXs (Fig. 1D) and where its knockdown resulted in a largely reduced (> 80% !) co-purification of NRX and PTPRs based on MS-analysis (Fig. 3D).

(2) ***Specificity of T178B knockdown on presynaptic function and target-specificity of sh-T178B***

Target-specificity of sh-T78B expression was assessed by proteome-wide MS-based analysis of protein amounts in four independent neuronal cultures, demonstrating consistent and highly selective reduction of T178 amounts by 96 - 98% (**new suppl. Fig. 9**).
Target-specificity of sh-T178B in the context of functional recordings was further demonstrated by AAV driving expression of sh-RNA that does not target any transcript. This sh-RNA failed to exert any effect on both sEPSC amplitude and paired-pulse ratios (sh-control, **new Fig. 3E**).

(3) ***Alterations in presynaptic function upon targeted removal of T178B, A in a double knock-out mouse model***

In addition to knock-down of T178B, we have established double conditional mutant mice (T178A,B flox/flox, see **new Methods part**) and induced knock-out of proteins T178A and B by viral expression of the Cre-recombinase.

Interestingly, MS-based assessment of protein amounts provided results very similar to those obtained with sh-T178B (**new Suppl. Fig. 9A, C**).

For functional analyses, Cre-driving AAV was injected into the entorhinal cortex and followed by whole-cell patch-clamp recordings of AMPA- and NMDA-receptor-mediated EPSCs in dentate gyrus granule cells (GCs) in response to stimulation of the medial perforant path (MPP, see **new Suppl. Fig. 11**).
The respective results uncovered alterations in presynaptic functions reflected by paired-pulse ratios and increased release probability (input-output relation, variance analysis, **new Suppl. Fig. 11**) at unaltered AMPAR-/NMDAR-EPSCs
These genetic knockout data obtained from an additional synapse population further support our conclusion that T178 proteins stabilize NRXN-PTPR associations and that their removal impacts presynaptic properties.

A second weakness is the framing of the ternary assembly as a "universal presynaptic core-module." This description is not supported. Synapses remain functional after T178 knockdown, and decades of genetic studies, including NRX triple knockouts and LAR-PTPR deletions, have consistently shown that synapse formation and function are remarkably resilient. Redundancy and compensation are built into the system, and this manuscript does not adequately grapple with that reality.

This may represent a misunderstanding. The term 'core-module' (of the identified networks) is based on the observations that (i) all of its three constituents are robustly co-purified in series of APs targeting proteins in the pre-synapse, the post-synapse, as well as in synaptic cleft (see Figs. 5, 6), and that (ii) the entirety of LAR-PTPRs and T178A, B are co-assembled with the NRXs (see Fig. 1D).
The term 'universal' was meant to reflect the abundant and wide-spread expression of the two sCAM families, NRX and LAR-PTPRs, established in a wealth body of work.

We agree with the reviewer, that synapse formation does not rely on the presence of the two tetraspanin proteins – as was explicitly noted in the original ms (Figures 3 and 4, and related text). On this background, we respectfully disagree on the reviewer's claim that we have neglected the activity of several sCAM proteins in synapses (also stated in the Introduction section).

We also think it necessary to add a note of caution on the cited NRX triple KOs and LAR-PTPR deletions:
Our ms shows - for the first time - that NRX1G, that was neglected in the triple KO strategy (by the Südhof group), amounts to as much as 20% of NRX1 thus leaving a considerable amount of ternary core-modules unaltered in the KO animals.
Moreover, there is considerable debate on the complete removal of PTPRs in the respective KO mice, as the deletion of the PTPR proteins has not been verified by quantitative MS.

Finally, the scope of the physiology and structural analysis is too narrow to support the sweeping claims. The AlphaFold-based docking predictions are intriguing but speculative and untested.

For the scope of the study, we refer to the **general remarks** above.

We have removed the structural model by AlphaFold (from the main body of the ms), as it is not central to the work here, although it nicely reflected all the results obtained

in our biochemical interaction analyses with the three constituents of the core-module (see **new Fig. 2E, F** and **new Suppl. Fig. 6**).

Moreover, the discussion underplays extensive prior literature on neurexin in order to present the ternary module as a paradigm shift, which it is not.

We have thoroughly revised the text of the ms to avoid misleading (over)statement(s) of results and conclusions (particularly in the Introduction and Discussion sections), as well as the impression of neglecting results from previous work which, in fact, was not intended at all. All changes are visualized by mark-ups in the text (tracking-mode and highlighting).

This work will only be suitable for publication after substantial new data and conceptual reframing. Most critically, the role of T178 must be established using genetic knockout rather than shRNA knockdown. Without KO models, the claim that T178 is required for the NRX-PTPR complex cannot stand, and the manuscript should not be considered further.

As stated above, we have newly acquired data on double KO animals and included them in the revised ms.

Functional analyses should be broadened to include direct measures of presynaptic release probability such as variance-mean analysis, vesicle pool labeling, or optical glutamate sensors rather than relying exclusively on paired-pulse ratio.

As pointed out above, we have extended the functional experiments using a double knockout model as described in **point (3)** above.
A detailed analysis of the impact of the newly identified T178 proteins is far beyond the scope of this study as detailed in the **general remarks** and, in fact, represents a major challenge as any alteration of individual constituents (or strings) in protein networks.

The authors should also assess whether the NRX–T178–PTPR module functions similarly across different synapse types, given the well-established circuit-specific roles of neurexins. Structural predictions must be validated experimentally, and the discussion should be revised to place the findings within the context of redundancy and prior adhesion molecule studies, rather than overstating novelty.

Again, we like to refer to the **general remarks** on the scope of this study and emphasize that the Discussion section has been revised, with focus on the extended set of proteomic data acquired here, the solid trans-synaptic protein networks (newly introduced here) and the implications of the results for future investigations.

Reviewer 2

We thank the reviewer for their very positive assessment of our work (This is a high-quality and rigorous study that provides compelling biochemical evidence for a widely distributed presynaptic protein assembly involving Neurexins, LAR-PTPRs, and tetraspanins. The experimental design and execution are exemplary, and the dataset will be a valuable resource for the field. While some of the broader interpretive claims could be toned down, the overall contribution is clear and important. I support publication of this work after minor revisions aimed at sharpening the conceptual framing and clarifying a few overstated points) and appreciate their suggestions for improvement that were incorporated into the revised manuscript as detailed below (in a point-by-point mode):

The authors propose that this ternary complex forms a “core module” for synapse formation and argue that its identification marks a “paradigm shift” in our understanding of synapse-organizing factors. While the study makes an important structural and molecular contribution, this interpretive framing may go somewhat beyond what the functional data support. The designation as a “core module for synapse formation” suggests an essential role in assembly, yet knockdown of T178B leads to relatively moderate changes in paired-pulse ratios and redistribution of Neurexins, without impairing synapse formation or causing overt transmission failure. Thus, although the ternary complex likely plays a widespread and conserved role in presynaptic architecture, its indispensability is not firmly established. A more restrained interpretation - as a scaffolding platform that coordinates trans-synaptic linkage and stabilizes molecular assemblies - might better reflect the data.

Along similar lines, the paper frames the coordination of multiple sCAM families as a major unresolved question and implies that the findings provide a new conceptual model. However, extensive prior work has shown that sCAMs are expressed in overlapping, combinatorial patterns and contribute to synapse-type diversity. The current study adds important structural and proteomic resolution to these observations but does not necessarily shift the conceptual ground. In this sense, the novelty is more technical and molecular than paradigmatic.

Finally, some statements could be clarified for precision. For example, the phrase “strong preference for (localization to) active zones” in the EM section should be more explicit: Neurexins are enriched at active zones relative to extra-synaptic membrane regions. These details are important to convey the findings accurately and avoid ambiguity.

We have carefully revised the text of the ms, in particular pointing out the role of the ternary core-module for *synapse organization* (as indicated in the ms title) and *organization of (trans)-synaptic networks* (as stated by the reviewer), and avoiding any misleading (over)statements.

Another point that could be reconsidered is the use of AlphaFold to propose a membrane-proximal structure of the complex. While such modeling is increasingly common, in the absence of experimental validation (e.g., crosslinking-MS, mutagenesis), the AlphaFold-based structural predictions remain speculative. Given the strength of the biochemical data, the model adds relatively little and might be better relegated to a supplementary figure with appropriate disclaimers.

We moved the structural predictions by AlphaFold into the supplement as it is not central to the work here, although it nicely reflected all the results obtained in our detailed biochemical interaction analyses with the three constituents of the core-module (see **new Fig. 2E, F** and **new Suppl. Fig. 6**).

One weaker aspect of the manuscript is the physiological readout. The increase in paired-pulse ratio upon presynaptic T178B knockdown is consistent with a presynaptic effect but remains difficult to interpret mechanistically. Without additional functional assays, it is hard to connect the observed biochemical reorganization to specific changes in neurotransmitter release, vesicle dynamics, or synapse function. This limits the physiological insight and makes the functional consequences of complex disruption somewhat abstract.

We have corroborated the functional effects on presynaptic function(s) observed upon knockdown of T178B (by sh-RNA) by experiments using knockout of T178A, B in a mouse model (T178A,B flox/flox, see **new Methods part**).
Accordingly, knockout of T178A,B in the entorhinal cortex led to alterations in presynaptic functions monitored in whole-cell recordings of AMPAR- and NMDAR-mediated EPSCs in dentate gyrus granule cells (GCs) in response to stimulation of the medial perforant path (MPP, see **new Suppl. Fig. 11**).
The results uncovered altered paired-pulse ratios and increased release probability (input-output relation, variance analysis, **new Suppl. Fig. 11**) at unaltered AMPAR-/NMDAR-EPSCs, very similar to the results obtained with the T178B-knockdown (Fig. 3E).

Reviewer 3

We appreciate the reviewers' positive comments
(Overall, this manuscript presents a conceptually significant idea that defines a novel presynaptic core module bridging two well-characterized adhesion systems in synapse organization),
as well as their criticism that prompted additional experiments included into the revised manuscript as detailed below in the responses to the reviewer's comments.

Major concerns:

1) AlphaFold docking & structural inference: The paper uses BN-PAGE, reverse-APs and AlphaFold predictions to infer the ternary structure, which provides valuable yet insufficient validation of the direct NRX-T178-PTPR contact.
Adding crosslinking-MS experiments the meAP of this complex would address the following:
a) Direct contact evidence to support the proposed 1:1:1 ternary structure and the AlphaFold docking.
b) The BN-PAGE result is consistent with the 1:1:1 stoichiometry, yet it's indirect in the fact that glycosylation could skew the results, so crosslinking MS would help solidify the results.
c) Prove T178-dependent interfaces for its function as the structural 'bracket'. Comparisons between T178B-present and T178B-absent states to prove T178-dependent stabilization of NRX-PTPR interaction.

In order to further corroborate the interactions between the three core-module constituents, we added AP-results of pairwise expressions in culture cells to the revised ms that show direct (and effective) binding of T178B to both PTPRS and NRX1G/3A, as well as weak interaction between NRX1A/G and PTPRS in the absence of T178B (**new Figure 2E**, **new Suppl. Fig. 6**).

Moreover, we documented the profound stabilization of the NRX1A-PTPRS interaction by T178B in the revised ms by quantitative MS-measurements demonstrating that T178 increases the stability of the NRX1A-PTPRS interaction by as much as a factor of 10 (**new Figure 2F**).

According to the reviewer's suggestion, we have also performed crosslink MS experiments:
We transiently expressed TwinStrep-tagged T178B together with PTPRS and NRX1G, purified the complex by StrepTactin Sepharose and eluted it natively with desthiobiotin. We then applied three MS cleavable, homo-bifunctional crosslinkers (DTSSP-D8/H8, DHSO, BMSO), with different concentrations and incubation times. Although crosslinking efficiently generated high-molecular weight protein assemblies (see **Addendum to Suppl. Fig. 6**), in-gel digestions (trypsin +- alpha-lytic protease) of respective band sections and subsequent LC-MS/MS analysis failed to identify any cross-linked peptide (MS analyses were performed on a HF-X mass spectrometer with adapted settings, MASCOT and xiSEARCH software were used for database search and intermolecular crosslink identification). This failure in MS-detection may well be explained by the hydrophobic nature and limited number of reactive side chains within the transmembrane domain of the complex.

2) T178A redundancy: The paper only interrogates the function of T178B through T178B knockdown, yet the role of T178A is unexplored. Does the expression level of T178A change in response to T178B knockdown? Is there any potential T178A redundancy that masks some stronger phenotypes? Dual perturbation of T178A and T178B could be a good way to address this possibility. Alternatively, if these experiments are not currently feasible, the authors should at least acknowledge the possibility that T178A could have certain functions and may mask some phenotypes. Additionally, the authors should discuss the expression level of T178A and T178B in a cell-type and brain-region specific manner based on existing datasets in the literature.

The expression pattern of T178A illustrated by the transcriptome data taken from the DropViz database (Saunders et al., 2018) in Suppl. Fig. 4, data on protein amounts determined by MS are given in Suppl. Fig. 9, together with the demonstration that the very low amounts of T178A are independent from the expression of the high amounts of T178B (**new Suppl. Fig. 9**).
Moreover, we have added functional recordings on dentate gyrus granule cells (GCs) demonstrating that the double knockout of T178A,B in their afferents originating from neurons in the entorhinal cortex leads to a similar presynaptic phenotype as obtained with the knockdown of T178B (see **new Suppl. Fig. 11**).

3) The paper does not dissect excitatory and inhibitory synapses when showing NRXs localization in the T178B knockdown condition in Figure 4E. This was done for the WT condition, but curiously missing from the knockdown data. The authors should also do analyses on excitatory versus inhibitory synapses in the T178B knockdown condition. Could the consequences of T178B differ between the synapse types?

As suggested by the reviewer, we have analyzed the distribution of NRXs (by immuno-EM on freeze-fracture replicas) before and after knockdown of T178B using double-labelling for NRX and either VGLU1 (for excitatory synapses) or VIAAT (for inhibitory synapses). While this procedure provided high-quality distribution data for excitatory synapses, we did not obtain this quality for inhibitory synapses (despite several trials). We, therefore, decided to include the data on profound re-distribution of NRXs upon T178B knockdown for excitatory synapses, together with the data obtained in single NRX-labelling experiments (**new Fig. 4C, E**).

4) There are several concerns need to be addressed in Figure 3A:
a) The data shown here seems to disagree with the data of ER-specific membrane analysis shown in Figure 3C, since none of NRXs, T178 or PTPRs appear in the ER cluster.
b) The authors should clarify the significance of Figure 3A in the general context. If it aims at showing the clustering of the ternary structure components, it makes more sense to perform covariation analysis of protein levels instead of organellar clustering. See the following literature for analysis of this type of data using clustering algorithms (PMCID: PMC6338729; PMC7984842)

The t-SNE plot (of an organellar proteomics approach) in Figure 3A was meant to illustrate that the preferred (subcellular) localization of the NRX-T178-PTPR complex is the plasma membrane (by co-appearance of these proteins with PM markers). As t-SNE places proteins according to their dominating features (i.e. abundance), ER-localization is not seen here. We have added the distribution profiles of the core-module constituents together with the averages of PM and ER markers across all 31 fractions to the revised ms (as **new Suppl. Fig. 7**).
Molecular appearance of the core-module constituents in the ER was investigated by csBN-MS on an ER-enriched fraction shown in Fig. 3C.

5) In Figure 3C, the authors should show that the ER membrane-enriched fraction is indeed ER-membrane enriched by using ER-specific markers (either MS or immunoblot).

This ER-enriched fraction has been established in our previous work (Schwenk et al., 2019; cited in this work) using ER-markers as mentioned by the reviewer (see main paper and Suppl. Figs. S1, S2 of Schwenk et al., 2019).

6) The authors should tone down and not use the word ‘universal’ to describe the complex given that they don’t show that the complex is universal at various types of synapses such as dopaminergic, serotonergic synapses, etc., or across even brain regions.

We have thoroughly revised the text of the ms and also replaced the word ‘universal’ which, in fact, was meant to refer to the well-established widespread and highly abundant expression of the NRXs and LAR-PTPRs.

Minor concerns

7) Please add replicate-level statistical significance analysis into the figures throughout the paper.

Done as suggested.

8) The authors should give more introduction to the tetraspanins before hypothesizing T178A/B to be part of the ternary complex.

Currently, there is no information on the two tetraspanins T178A, B neither in literature, nor in other public databases that we searched.

9) The paper claims that ‘these analyses showed that the major portion of NRXs is covalently linked to CAH10’ in Figure 1D. Please remove “covalently” or explain how the data supports a covalent interaction is shown, given that AP experiments don’t necessarily indicate covalent binding.

Covalent binding of CAH10 to NRX has been established in earlier collaborative work with TSüdhof (see Sterky et al., 2017).

10) Please provide the AlphaFold3 docking scores (pTM and ipTM) in Figure 2F. If these are low (pTM below 0.5 or ipTM below 0.6), then the crosslinking MS data may help by incorporating the data into AlphaLink using ColabFold:

The parameters of the AlphaFold predictions/modelings were added to the legend of the new Suppl. Fig. 6B.

11) Please edit for consistent hyphen use in terminology in figures and text. For example, presynaptic versus pre-synaptic.

Done as suggested.

12) Page 4 typo: ‘fast majority’ should be ‘vast majority’.

The typo was corrected.

Responses to the reviewers‘ comments

Reviewer 1

The authors have improved the manuscript somewhat; however, major conceptual and technical issues persist that cast significant doubt on their central claim regarding the purported Neurexin T178 PTPR core module.

In the revision, we provided analysis of TMEM178 conditional double KO mice, and our findings confirmed and extended the previously provided data. We are pleased that the reviewer did not raise any concerns about this new data addressing their major concern.

Overall, we note that Reviewer 1 did not engage with the new data and our responses to the original comments directly, but instead raised a new set of conceptual and technical objections. Several of these concerns reflect misunderstandings of the quantitative proteomics approach and related analyses, as detailed in our point-by-point response. Regardless, we added new data to the second revision of the manuscript to address these new points.
At the same time, we feel that these new comments extend beyond the scope of the major issues raised in the earlier round, such that the review process no longer appears fully focused on evaluating whether the key prior concerns have been satisfactorily addressed.

As is evident across all figures involving T178 knockdown (for example, Figure 3D), PTPR levels are dramatically reduced following T178 depletion, and the lysates clearly show a marked loss of PTPRs. In this context, the Neurexin1-3 affinity purifications still retrieve PTPRs in proportion to its abundance in the T178 knockdown lysates, indicating that the apparent loss of Neurexin-bound PTPR largely reflects reduced PTPR levels rather than a specific disruption of a Neurexin-PTPR interaction.

The reviewer’s point was carefully addressed:

Removal of T178B by either knock-down (KD) or knock-out (dKO) reduced the overall amounts of PTPRs by 30-40% (determined by quantitative MS, **Supplementary Figs. 9A, C**) resulting in a reduced retrieval of PTPRs in anti-NRX1-3 APs (**Fig. 3D**).

However, the actual decrease in amounts of co-purified PTPRs exceeds by far the amount expected from the reduced overall PTPRs levels. This profound over-proportionality strongly supports the proposed T178B-mediated stabilization of the interaction between NRX and PTPR (by bracketing).
The data on the over-proportional reduction in co-purification was added to the revised ms as **new Supplementary Fig. S9D**.

Furthermore, in the co-expression experiments (Figure 2E and all panels of supplementary Figure 6A), the PTPR subunit appears unstable and cleaved in the absence of T178, consistent with previously described γ-secretase-dependent cleavage or cleavage by Adam10 or 17 of related PTPR family members (see Haapasalo et al., 2007, JBC,).

We agree with the reviewer that only conditions with equivalent protein levels of the complex constituents can be evaluated. We note that the levels of full-length PTPRS are very similar among all the conditions used.

There is indeed a lower MW band of the P-subunit of PTPRS, likely a by-product of heterologous expression (not observed in brain), in the absence of T178B (i.e. in unphysiological conditions). However, although all forms of PTPRS are effectively purified in anti-V5 (PTPRS) APs, effective co-purification of NRX only occurred in the presence of T178B, while it was largely reduced in the absence of the tetraspanin (**Fig. 2E**, **Supplementary Fig. S6**).
Consequently, the potential differential susceptibility of PTPR to cleavage does not impact conclusions regarding the co-purification of the complex components.

We appreciate the comment and have now further clarified this in the legend of **Fig. 2E**.

Most concerningly, in their earlier biorXiv manuscript posted on July 19, 2024, the authors themselves reported that T178 knockdown in primary neurons reduced total PTPRS levels by approximately 78% and surface PTPRS levels by nearly 90%. This critical dataset, which speaks directly to how lack of T178 affects PTPR stability, has been omitted from both the current submission and the subsequent biorxiv version submitted on April 10, 2025.

The readout of PTPRS amounts in neuronal cultures was based on antibody surface-labelling and quantification of fluorescent staining signals. This method inherently displays a limited dynamic range and, therefore, has a marked tendency to overestimate differential effects.
After obtaining accurate MS-based quantification data for both KD and dKO cultures (**Supplementary Figs. 9A, C**) we had omitted these less quantitative data. In response to the reviewer's comment, we are happy to include the data from anti-PTPR staining in the final manuscript together with clarification on data obtained with MS vis-à-vis surface staining.

Taken together, the evidence strongly suggests that the primary role of T178 is to stabilize PTPRs and promote its surface expression, with any effects on protein-protein interactions such as with Neurexins being secondary. Indeed, the authors' own data (including their new Figure 2F) better support this interpretation than the speculative ternary complex model they continue to promote. The notion of a direct Neurexin T178 PTPR "core module" is therefore unsubstantiated and misleading; what the data actually reveal is a indirect or secondary effect driven by PTPR stability, a valid and interesting mechanism in its own right, but not the one claimed. Therefore, a reinterpretation of the results is warranted.

This point is largely addressed by the new data added and described above:

In the absence of the T178 proteins, the efficiency of co-purification of the NRXs (in *anti-PTPR* APs) and PTPRs (in *anti-NRX* APs) is substantially decreased. This decrease exceeds by far the degree of reduction expected by the decreased protein abundance of PTPRs (**new Supplementary Fig. S9D**).

Moreover, we like to emphasize here, that the APs from T178 knock-down are confirmatory of one of the **major results** indicating **assembly of NRXs, PTPRs and T178** into **ternary complexes**. These are:

(1) Assembly of the NRX-T178-PTPR complexes in the endoplasmic reticulum (ER, **Fig. 3C**, csBN-MS), from where they are transported to the plasma membrane, its final localization (**Figs. 3A**, **B**, organellar proteomics and anti-LRRT4-APs

(2) Common retrieval the NRX-T178-PTPR complex in series of APs targeting proteins in the pre- and post-synaptic membranes, and in the synaptic cleft (**Figs. 5**, **6**, tSNE-plots of meAP-MS experiments and summary of all meAP-MS data)

(3) Detail binding studies (**Figs. 2E**, **S6A**) demonstrating that T178 stabilizes the otherwise weak interaction between NRXs and PTPRs (**Fig. 2F**, MS-quantification of APs).

These findings unequivocally demonstrate that the T178 proteins promote the assembly of NRX and PTPR into **ternary complexes** (already early in biogenesis), that finally (in the plasma membrane) serve as **core-modules of stable trans-synaptic protein networks**. The order of assembly is not important here, nor can it be resolved.

The reported approximately 50% mislocalization of Neurexin under T178 knockdown is also problematic (Figure 4). Given the well-known challenges of reliable Neurexin immunostaining, immuno-EM quantification without knockout or knockdown validation lacks rigor and should not be considered definitive evidence.
Moreover, with that level of neurexin mislocalization, one would expect major deficits in basal synaptic function, which was absent in the author's own data, as well as in another work from Thomas Sudhof's lab (https://purl.stanford.edu/cp200pt0640). I would either remove this data or improve upon it with KO/KD control experiment for neurexin antibody validation in immuno-EM. The same can be said for Supplementary Figure 8.

We agree with the reviewer that the limited availability of reliable NRX antibodies for APs and immune-staining has been a major challenge for the field.

We used some commercial reagents (after rigorous MS-based validation) – but a major addition of our study has been the generation and validation of 10 new NRX antibodies (ABs).
A complete list of these new ABs is provided in the new **Supplementary Table 5**. The anti-NRX AB used for immuno-EM has been rigorously tested in MS-quantified APs indicating that this AB displays high target-efficiency combined with high target-specificity (very low background).

We note that the synaptic staining (with strong preference for active zones) obtained with this AB in wild-type tissue (**Fig. 4**) closely resembles the results reported in an independent peer-reviewed EM study (Szoboszlay, Kirizs and Nusser, 2017).

We have now further clarified this in the revised manuscript.

The reviewer's statements on expectations about the 'basal synaptic functions' neglects the observed alterations in synaptic transmission (**Figs. 3E**, **Supplementary Fig. S11**), and rather builds on a non peer-reviewed thesis from the Südhof-group. As correlations between EM-based analyses of NRX-distribution and function is currently not available in literature, the reviewer's expectations appear unsubstantial.
Moreover, it should be kept in mind that despite the re-distribution of NRX after T178 knockdown there is a considerable portion of roughly 50% remaining in the active zone (**Fig. 4E**). This point was added to the Discussion section of the revised ms.

The stainings illustrated in **Supplementary Fig. 8** used 'tagged' T178 proteins (T178-HA knock-in). We confirmed specificity of the HA-immune-reactivity by using WT sections as a rigorous control.

Finally, the manuscript fails to acknowledge extensive prior work demonstrating that the Neurexin-PTPR interaction is largely dependent on heparan sulfate modification of Neurexin (see for example, Han et al, J Neurosci, 2020 Oct 28;40(44):8438-8462 and Roppongi et al. Neuron, 2020 Apr 8;106(1):108-125.e12), a major omission that distorts the interpretive context. In Figure 2D, the authors assess heparinase effects on protein abundance by mass spectrometry, yet the assay buffer employed may contain detergents inhibitory to enzymatic activity. These experiments must be repeated under either detergent-free or heparinase-compatible conditions reported in previous reports, and validated by quantitative immunoblotting.

The reviewer raises an important point on the experimental details of heparinase treatment that may be overlooked.
The heparinase treatment was performed on native tissue using a protocol similar to that by Zhang et al., 2018 (without detergent contamination), and its efficiency was carefully assessed and documented (**Fig. 3D**, inset).

The impact of the heparinase treatment on synaptic complexes was quantified by MS-based analysis of subsequent anti-NRX APs. These analyses demonstrated substantial reduction in co-purification of LRRTs1-4 and GluA1,2, in line with published data (Zhang et al, 2018), but also indicated that co-purification of PTPRs D, S and T178 with NRXs was unaltered (**Fig.2D**).

Moreover, APs of GPI-anchored NRX3 that is heparan-sulfate (HS)-modified but lacks the transmembrane and cytoplasmic domains further support our conclusion: This NRX isoform no longer binds to both T178 and PTPRs (**Fig. 2C**).

Together, these results provide multiple lines of quantitative evidence that the NRX-T178B-PTPR complex assembles independently of HS and that HS-modification is neither required, nor sufficient for interaction.

The Roppongi et al study was cited in our manuscript.

We note that Roppongi et al and Han et al both used qualitative Western blotting approaches to assess the impact of HS-removal on PTPR-NRX complex formation and lack any form of quantification. Moreover, the experimental design and data quality raise questions about an essential role of HS in these interactions.
This is particularly true for the study by Han et al. (Fig. 11A-C). We invite the reviewer to re-evaluate their results – in particular for the interactions of endogenous proteins (panel C) to judge the quantitative impact of HS modification on PTPR interaction:

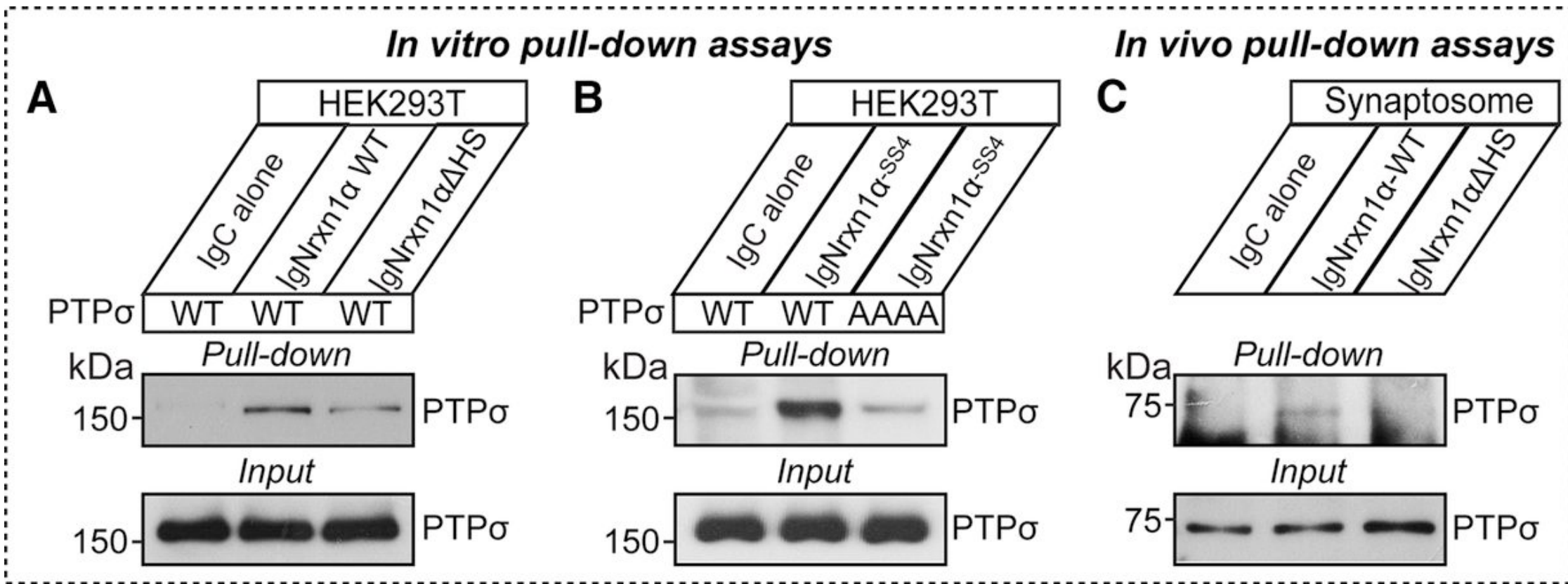

To address the reviewer's point, we clarified the experimental protocol for heparinase treatment in the revised ms, added the Han et al study to the references, and provided explicit explanation on the differences in the observations made in the Discussion section.

Likewise, the structural model in Figure 2G is not convincingly supported. Although the figure legend claims it is based on interaction assays (C–F), the model bears little resemblance to those data.

The structural working model depicted in **Fig. 2G**, in fact, accurately summarizes the results obtained in an extensive series of pairwise binding studies probing interactions between (i) T178B and NRXs, between (ii) T178B and PTPRs, and (iii) between NRX and PTPRS in the absence and presence of T178B, together with the lack of binding observed with NRX3-GPI (**Figs. 2C,E, F**, **Supplementary Fig. 6**). Accordingly, the model highlights independent interaction of T178B with both NRXs and PTPRs through their transmembrane domains and the T178B-mediated stabilization of the (otherwise weak) interaction between NRX and PTPRs.
We clarified this in revised figure legend.

Moreover, both UniProt and AlphaFold offer conflicting predictions for T178B topology (three versus four transmembrane domains, respectively), and the low confidence scores reported (pTM = 0.52; ipTM = 0.26) are well below accepted thresholds for a reliable multimeric model according to EMBL–EBI AlphaFold guidelines. ipTM values below 0.8 are considered failed predictions (https://www.ebi.ac.uk/training/online/courses/alphafold/inputs-and-outputs/evaluating-alphafolds-predicted-structures-using-confidence-scores/confidence-scores-in-alphafold-multimer/). Mutant-based domain mapping would be a considerably more convincing approach.

The statement of divergent topologies of T178B by AlphaFold (four α-helical domains of sufficient length to span the membrane) and UniProt (three predicted transmembrane domains) resulted from UniProt's uncertainty about the precise N-terminus of the T178 proteins.
We carefully analyzed this by MS and experimentally verified that the N-terminal domain is indeed translated *in-vivo*. This verifies the AlphaFold topology with a likely intracellular localization of the very N-terminus.

This new data from the MS-based sequence analysis has been added to the **revised Supplementary Figure S2**.

The indicated scores refer to the prediction confidence for the assembly of the TM-domains of NTXγ, T178B and PTPRS by AlphaFold (not the folding of the T178B protein). We have now clarified this in the figure legend.

Finally, we agree that the structural model depicted in **Fig. 2G** is a 'working model' – based on extensive biochemical evidence – and we have now explicitly stated this in the text.

In its current form, the manuscript overinterprets its results- one cannot draw conclusions of a protein interactions when the protein itself is unstable, omits critical contradictory evidence, and fails to adequately consider prior literature. The conclusions as stated are neither rigorously supported nor mechanistically credible.

In the second revision, we have made an additional effort to carefully review all statements in the text, added substantial new quantitative data, included additional detailed discussions of prior literature and provided further clarifications to address the sentiment voiced by this reviewer.
We note, that most of these comments made by the reviewer are related to data already included in the original manuscript and were newly introduced after we had satisfactorily addressed their previous set of 'major concerns'.

Other major concerns

1. It is erroneous to claim that the so-called Neurexin–T178–PTPR module "recruits stable and extended trans-synaptic protein networks with defined pre- and post-synaptic partners and secreted extracellular linkers." The authors provide no compelling evidence to support such an expansive statement other than co-isolation in mass spectrometry. The only other data presented are modest reductions in protein abundance detected by mass spectrometry, which cannot by itself establish recruitment, stabilization, or structural assembly of any trans-synaptic network.

This statement (in the abstract) is based on the outcome of the large series of APs that targeted proteins in all sub-compartments of the synapse, the pre- and post-synaptic membranes, as well as the synaptic cleft (total of 30 different constituents, **Fig. 6**, upper panel).

These APs specifically retrieved - at astonishingly high degrees of consistency (illustrated in the t-SNE plots of **Fig. 5**) and abundance - large numbers of proteins (see **Fig. 6**, and **Supplementary Table 4**) from all synaptic compartments, many of them previously unrecognized in relation to NRXs.
Both parameters, consistency and abundance, unequivocally indicated the existence of protein networks that are (i) sufficiently stable to enable their isolation by APs, that (ii) extend from the pre- to the post-synaptic membrane and that (iii) contain the NRX-T178-PTPR module as a common constituent or core-module.

We acknowledge that our proteomic approaches characterize complexes and larger protein assemblies in their 'steady-state' (i.e. across all stages of synaptic assembly present in the adult brain), and accordingly provide indirect insight into the dynamics (sequences) of the 'recruitment' processes. Strengths and limitations of our proteomic approach are detailed in the Discussion section of the revised ms.

There is no demonstration that synaptic networks are altered, destabilized, or "collapsed" in the absence of T178, nor is there biochemical, imaging, or functional evidence of complex assembly. This sweeping mechanistic claim is therefore unsupported by rigour and represents a serious overreach beyond the data shown. At best, the results suggest a change in steady-state protein levels, not the recruitment or maintenance of a defined trans-synaptic architecture.

We note that the quoted word "collapsed" is/was not used in our manuscript.

In fact, there are numerous evidences for the impact of the targeted removal of the T178 proteins, including biochemistry indicating changes in protein levels (**Supplementary Fig. S9**), proteomic (interaction) analysis showing substantial alterations of synaptic networks including major destabilization of the interactions with NRXs and PTPRs (**Fig. 3D**) and patch-clamp recordings demonstrating alterations in synaptic transmission (**Fig. 3E**, **Supplementary Fig. S11**).

Moreover, our quantitative immuno-EM in freeze-fracture replicas (**Fig. 4**) demonstrate profound re-localization of NRX in the pre-synapse, clearly going beyond "steady-state protein levels".

2. The authors state that "all three NRXs co-assemble with T178B and LAR-PTPRs into previously unrecognized stoichiometric complexes," yet this claim is not unique to PTPR-Neurexin interaction based on their own data. In Figure 1D and 1E, several additional proteins show comparably robust co-assembly with T178B and LAR-PTPRs, indicating that formation of such stoichiometric complexes is not specific to Neurexins and therefore cannot be taken as definitive evidence for a privileged Neurexin-T178-LAR-PTPR module.

The reviewer raises an essential point about the complexity of NRX-associated assemblies comprising several previously known and newly identified constituents (see t-SNE in **Fig. 1C**, **Figs. 1D, E** and **Table 1**).

It must be emphasized, however, that although a number of proteins are consistently co-purified in several of our anti-NRX APs (**Figs. 1D, E**) – indicative for the solid trans-synaptic networks – only the three constituents of the ternary complex NRXs, LAR-PTPRs and T178 are effectively and consistently retrieved in the APs with multiple ABs targeting proteins in all synaptic compartments (t-SNE plots **Fig. 5**, summary plot **Fig. 6**, lower panel).

The unique near stoichiometric recovery of NRXs, T178 and PTPRs in these 'synaptic' APs (**Figs. 5, 6**) highlights the particular importance of these three constituents in the trans-synaptic networks and, together with the clear-cut binding studies, led to the conclusion about the core-module. We have now further clarified this important point in the revised ms when discussing the t-SNE plots related to **Figs. 2**, **5**.

3. On page 6, the authors state: "In addition, these analyses showed that the major portion of NRXs is covalently linked to CAH10 and interacts with the secreted CBLN1 protein." However, if most neurexins are bound to CA10 and thereby lack HS, it remains unclear why endogenous neurexins still run as broad smear bands, which typically indicate heterogeneous HS modification. In addition, if CA10-bound, HS-deficient neurexins are indeed the predominant species, it is difficult to reconcile this with the strong synaptic and behavioral phenotypes reported in Nrxn1ΔHS mice, where only the HS attachment site on Nrxn1 is mutated (see Zhang et al, 2018, Cell). Please clarify how your model accounts for the apparent abundance of HS-modified neurexin species on SDS-PAGE and explain why disrupting HS at a single

neurexin locus (Nrxn1ΔHS) produces such pronounced phenotypes despite the proposed dominance of CA10-bound, non-HS neurexin complexes.

We appreciate the reviewer's comment, but we do not see any major discrepancy to published data.
We note that CAH10 is observed on more than 50%, but not all of the NRX-containing complexes; the wording "major portion" may have been misleading here. Association with CAH10 modifies, but does not prevent HS-modification of NRX. Profound alteration of HS modifications was only reported for non-physiological over-expression of CAH10 *in vitro* (Montoliu-Gaya et al, 2021).
In line with a previous report (Zhang et al., 2018) we find that enzymatic HS-removal markedly impairs interactions of NRX with trans-synaptic ligands (**Fig. 2D**). This explains the pronounced phenotypes in the Nrxn1ΔHS mice.
We modified the wording and explained this in the revised ms.

Minor concerns

1. The claim that PTPRD was identified as a new partner of neurexin is not correct. It was already shown before, see Han et al, J Neurosci, 2020 Oct 28;40(44):8438-8462. Please remove this claim.

See comments above. We added a clarification to the revised manuscript.

2. Another concern is how the authors ensured that the analyzed immune-EM synapses actually originated from T178 knockdown neurons, given that no infection reporter is visible in the EM images. In Fig. 4E, it is also unclear why the WT condition combines excitatory and inhibitory synapses, whereas the T178 KD condition is shown as "all synapses" and "excitatory synapses" separately; the rationale for this mismatch in sampling and presentation should be explained. Since T178 KO or tagged mice are available, the authors should instead (or additionally) validate and directly confirm synaptic localization of T178 in situ.

We note that the risk of having non-infected neurons/synapses included in our quantification would result in under-estimating the actual phenotype in this loss-of-function model.
The clarification (on the EM data) is provided in the revised ms.

We included data on the synaptic localization of tagged T178 (T178B-HA knock-in mouse model) in **Supplementary Figure S8**. Wild-type animals were used as negative controls to ensure the specificity of the reported immune-reactivity.

3. I recommend that the authors include a table summarizing all antibodies used for AP, WB, and IHC, indicating for each antibody the target, host species, source/catalog number, dilution, and the specific experiments/figures in which it was used, to facilitate interpretation and enhance reproducibility.

The requested information was already available through the r**eporting summary** of the journal AND through **Supplementary Table 1**. To further clarify this, we have now added an additional **Supplementary Table 5** summarizing all necessary data on the ABs used in this study.

Bottomline and recommendation
My main concern is that, in light of prior reports demonstrating heparan sulfate–dependent neurexin–PTPR interactions, together with the evidence in this study that T178 primarily affects PTPR levels (and surface expression in the original bioRxiv version), as well as data from the Südhof lab showing minimal synaptic phenotypes upon removal of T178A/B, the current conclusions proposing a functionally significant ternary complex are not adequately supported by the available evidence and appear substantially overstated.

As outlined in the point-by-point response above, we have now provided extensive evidence addressing not only the “major points” raised by this reviewer on the first submission, but also the newly raised points that were brought up when commenting on the revised manuscript.

As for the prior reports onto proposed direct heparan-sulfate-dependent neurexin-PTPR interactions, we hope that the reviewer can now directly evaluate the rigor and quantitative nature of our experiments vis-à-vis the two previous non-quantitative studies. We feel that it should be possible for science to advance and to correct the literature when improved reagents (in our case a series of new highly specific ABs) and methods (deep, quantitative MS-analysis) become available.

The newly included quantitative data on the decreased NRX-PTPR interaction upon T178 removal (**Supplementary Fig. 9D**) directly addresses the reviewer’s concern regarding PTPR levels influencing our conclusions.

Finally, we note that the data by the Südhof lab are non-peer-reviewed. Moreover, they were acquired using dissociated neuronal cultures – an experimental system that does not recapitulate neuronal circuitry and where synapses form between cell populations that do not preserve the selective connectivity of neural circuits. By contrast, we collected data from an acute slice preparation which largely preserves native connectivity and physiological properties.

Nevertheless, it is notable that the Südhof data (while not yet vetted through peer review) support a major impact of the constitutive double knock-out of T178A,B considering their suggestion that double knockout mice have substantially reduced survival and alterations in behavior.

As currently framed, the strong emphasis on a ternary complex risk giving a misleading impression to the field and could propagate an incorrect conceptual framework. I therefore recommend that the claims about T178 as the mediator of ternary complex formation be thoroughly reconsidered, instead the emphasis should be on how T178 is regulating stability of PTPRs and its lack thereof predictably affects its complexes as a secondary outcome. This should be emphasised throughout the manuscript, including in the Title, Abstract and Discussion. Given that the authors are now developing tagged mouse lines and additional tools, definitive statements about the existence and mode of interaction of a ternary complex should, in my view, be deferred to future studies employing rigorous structural biology and functional analyses. Provided that the authors substantially revise, moderate, and clearly qualify their claims about the ternary complex along these lines, I would be supportive of publication, as the proteomics data appear to be of high quality and will constitute a valuable resource for the field.

We carefully reviewed and considered the statements in the text. The stability of the NRX-T178-RPTP complexes observed across an array of affinity-purifications (**Fig. 2** and **Fig. 5**) and the clear dis-proportional loss of PTPR recovery in NRX

complexes isolated from T178 knockdown models (**new Supplementary Fig. S9D**) all indicate that T178 is firmly interlinking NRXs and PTPRs.

We thank the reviewer for further encouraging us to provide these additional data to further pressure test the conclusions of the work.

Reviewer 2

From my perspective, the authors have responded thoughtfully and constructively to the reviewer's comments, appropriately refining their claims and improving the clarity of the manuscript. In its revised form, I am pleased to support acceptance.

We very much appreciate the reviewer's statement and their strong support of our work.

Reviewer 3

We truly appreciate the reviewer's comments that improved and strengthened the ms and we welcome their clear statements (highlighted in red) on the changes/additions we provided in the (first) revision.
Moreover, we explicitly welcome their final statements on the value of our work.

We thank the authors for carefully addressing our comments and revising the manuscript. The manuscript has improved substantially and all of our concerns have been addressed, with just one remaining textual change request.

Concerns that were adequately addressed
Structural validation (original concern #1): We appreciate the authors' attempt to perform crosslinking-MS. While it did not yield crosslinked peptides, this is understandable. Importantly, the authors added new pairwise binding data from heterologous expression and quantitative MS measurements demonstrating that T178 increases the stability of the NRX1A–PTPRS interaction by approximately 10-fold. These data corroborate the role of T178 as a structural bracket that stabilizes the ternary complex. *Relocating the AlphaFold model to the supplement is appropriate given that it remains unvalidated and other reviewer's suggested this as well.*

T178A redundancy (original concern #2): The authors added functional recordings on dentate gyrus granule cells using a T178A,B double conditional knockout mouse, as suggested. This is a substantial experimental addition that goes beyond what we originally requested. The double KO in entorhinal cortex produces presynaptic phenotypes (altered paired-pulse ratios, increased release probability) consistent with the T178B knockdown data, which largely addresses the concern of T178A redundancy and also successfully addressed Reviewer 1's objection about reliance on shRNA (in my opinion).

Subcellular fractionation and other concerns (#4–6, #7–12): We appreciate that the authors added the distribution profiles of the core-module constituents together with averages of PM and

ER markers across subcellular fractions (new Suppl. Fig. 7), showing that the core-module constituents have high relative abundance at the same fractions. The replicate-level statistics, textual revisions (removal of "universal," toned-down framing), and all other minor concerns have been properly addressed in the revised manuscript.

One remaining minor issue

Excitatory vs. inhibitory synapse analysis (original concern #3): The authors attempted to analyze the distribution of NRXs after T178B knockdown at inhibitory synapses, as requested, but did not obtain distribution data of comparable quality to excitatory synapses. We accept this technical limitation. However, the revised manuscript does not explicitly acknowledge this gap. Given the well-established circuit- and synapse-type-specific roles of neurexins, the authors should mention their attempt to address this question in the manuscript text and acknowledge T178B's contribution to NRX localization and presynaptic function may differ at inhibitory versus excitatory synapses. A brief statement in the Results section (around Figure 4E) and/or in the Discussion would suffice.

We have added respective notes for clarification to the revised ms (Results, Discussion, and legend to **Fig. 4E**) as requested by the reviewer.

In summary, the revised manuscript has been significantly improved and represents a strong contribution to the field. The addition of the double conditional knockout mouse model was a substantial and well done addition and it greatly strengthens the conclusions.

Reply to Rev1

Despite the additional work and clearer presentation in the revised manuscript, I remain unconvinced that the new data resolve the main interpretive problems raised in my initial and follow-up review. The central conceptual issue remains unresolved: in my view, the data are fully compatible with T178 acting primarily to stabilize PTPR expression and surface delivery, with any changes in Neurexin-PTPR association arising as secondary consequences of altered PTPR availability, whereas the manuscript still presents a privileged ternary Neurexin-T178-PTPR “core module” as an established mechanistic entity, thereby overstating what can be rigorously inferred from the experiments. I outline below where I see persistent gaps between data and claims, and how a more conservative, quantitatively grounded re framing could better align the conclusions with the available evidence

We note that Reviewer 1 evaluates the T178B loss-of-function experiments in isolation and did no longer consider the extensive body of MS-data and biochemical/proteomic experiments provided in the original submissions.

As the reviewer’s comments appear to no longer focus on evaluating whether their prior concerns/questions have been satisfactorily addressed, **we will answer their newly raised points in summarizing manner below**.

The main corps of experimental data demonstrating the formation of ternary PTPR-T178-NRX complexes (with strong stabilizing effect of T178B) has been explicitly given in our last response.

These are:

(1) Assembly of the ternary NRX-T178-PTPR complexes in the endoplasmic reticulum (ER, **Fig. 3C**, csBN-MS), from where they are transported to the plasma membrane, its final localization (**Figs. 3A**, **B**, organellar proteomics and anti-LRRT4-APs

(2) Common retrieval of the NRX-T178-PTPR complex in series of APs targeting proteins in the pre- and post-synaptic membranes, and in the synaptic cleft (**Figs. 5**, **6**, tSNE-plots of meAP-MS experiments and summary of all meAP-MS data)
Moreover, T178B and LAR-PTPRs are the most abundantly co-purified proteins in anti-NRX1-3 APs (**Fig. 1D**).

(3) Detail binding studies (**Figs. 2E**, **S6A**) demonstrating that T178 stabilizes the otherwise weak interaction between NRXs and PTPRs (**Fig. 2F**, MS-quantification of APs). Explicitly, the NRX-PTPR interaction is increased by 10-fold, or in other words, 9 out of 10 NRX-PTPR interactions are lost/unstable in the absence of the T178.

These findings unequivocally demonstrate that the T178 proteins promote the assembly of NRX and PTPR into **ternary complexes** (already early in biogenesis), that finally (in the plasma membrane) serve as **core-modules of stable trans-synaptic protein networks**. The order of assembly is not important here, nor can it be resolved by currently available techniques.

Specific concerns:

1. T178, PTPR stability, and “over proportional” loss

(with points a-c, referring to formation of the ternary PTPR-T178-NRX complexes, where T178 stabilizes the otherwise weak interaction of PTPR and NRX)

In contrast to the reviewer’ s statement, the newly added **Suppl. Figure 9D** does provide all the data requested (on quantification of the over-proportional loss of PTPRs in anti-NRX1 APs after KD of T178B) – we assume that there has been some misunderstanding here.

2. Claims of a privileged ternary “core module”

and closely related

5. Structural model and topology of T178B

The reviewer’ s view (and interpretation) consistently neglects the large body of experimental data/findings listed above demonstrating (i) formation of the ternary NRX-T178-PTPR complexes, AND (ii) their action as a ‘core-module’ of the stable synaptic protein networks uncovered in this work.
The latter is reflected by the **unique retrieval of all three NRX, T178 and PTPRs** in a multitude of APs targeting a broad variety of network constituents, and in the organellar proteomics approach (**Supplementary Figure 7**).

The KD of T178 resulted in profound disturbance of the assembly of the core-module AND their interaction with selected partners (Figs. 3); comprehensive analysis of the synaptic networks introduced in this study (for WT) appears far beyond the scope of this paper.

The arrangement of the three constituents within the ternary complex is presented as a ‘working model’ (stated as such on p. 7) that reflects the results of the pairwise interactions unequivocally identified in heterologous expressions AND the stoichiometry that is derived from the apparent molecular mass in native gel separations (**Figs. 2A, 3C**).

Analysis by AlphaFold 3.0 is provided as a ‘structural model’ – it does not replace structural analyses, but represents an independent line of evidence.

3. Neurexin mislocalization and functional consequences.

As stated in our previous response, the AB used for immuno-EM was rigorously evaluated (in MS-quantified APs indicating that this AB displays high target-efficiency combined with high target-specificity (very low background)), and it provides essentially the same staining efficiency in both WT and T178-KD tissue thus perfectly mirroring the quantification of protein amounts by MS.

Unfortunately, the NRX AB cannot be tested on KO tissue, as it targets all NRX paralogues and there are (currently) no mice with a complete deletion of NRX1-3.

Notwithstanding, we carefully stated the results in the ms as a ‘suggestion’:

Together, these immuno-EM results demonstrated preferred localization of NRXs to the active zone of synaptic boutons as previously observed [52] and **suggested that this preference is due to co-assembly with T178B**.

4. Heparan sulfate dependence and prior literature.

All the reviewer's points have been explicitly addressed before:

The impact of the heparinase treatment on synaptic complexes was quantified by MS-based analysis of subsequent anti-NRX APs. These analyses demonstrated substantial reduction in co-purification of LRRTs1-4 and GluA1,2, in line with published data (Zhang et al, 2018), but also indicated that co-purification of PTPRs D, S and T178 with NRXs was unaltered (**Fig.2D**).

Moreover, APs of GPI-anchored NRX3 that is heparan-sulfate (HS)-modified but lacks the transmembrane and cytoplasmic domains further support our conclusion: This NRX isoform no longer binds to both T178 and PTPRs (**Fig. 2C**).

Together, these results provide multiple lines of quantitative evidence that the NRX-T178B-PTPR complex assembles independently of HS and that HS-modification is neither required, nor sufficient for interaction.

In contrast to the reviewer's statement:
Not a subset, but all NRX-T178-PTPR complexes persist after heparinase treatment, demonstrating that HS, different from the transmembrane domain of NRX, is required for complex formation.

Despite our clarifying response, the reviewer takes previous work on HS-dependent neurexin–PTPR interactions as ground truth and, suggests that our results and conclusions cannot be accurate. This previous work has substantial methodological weaknesses and our study clearly provides multiple lines of rigorous, quantitative measures that support a major function of T178A,B in forming a ternary complex with neurexins and PTPRs.
We feel that such a correction of the literature is a normal element of the scientific process.

6. Scope of claims and recommended re framing

The suggested changes are unfortunately not in line with our experimental data, but rather reflect the reviewer's interpretation.
We, therefore, respectfully decline to introduce the suggested changes.
